# The potential functions of *HvDJ* genes in regulating salt tolerance in barley

**DOI:** 10.3389/fpls.2025.1574097

**Published:** 2025-07-10

**Authors:** Yunfeng Xu, Haoran Sun, Ling Shen, Boyan Wan, Lijun Liu, Guoping Zhang, Qiufang Shen

**Affiliations:** ^1^ Institute of Crop Science, College of Agriculture and Biotechnology, Zhejiang University, Hangzhou, China; ^2^ Zhejiang Key Laboratory of Crop Germplasm Innovation and Utilization, Hangzhou, China; ^3^ Zhejiang University Zhongyuan Research Institute, Zhengzhou, China

**Keywords:** *HvDJ*, barley, cis-regulatory elements, phylogenetic analysis, salt response

## Abstract

The important roles of *JDP* members in regulating abiotic and biotic stress tolerance have been demonstrated in many plants. However, fewer studies have explored the *JDP* gene family and its role in the salt stress response in barley, a crop known for its superior salt tolerance compared to other major cereals. Here, we identified a total of 109 putative *JDP* genes (nine *HvDJAs*, eight *HvDJBs*, 92 *HvDJCs*) in barley. Promoter analysis of *HvDJs* suggested that HvDJs might be involved in the processes of hormone regulation and stress response. Tandem and segmental duplications appear to be the driving forces behind *JDP* gene family expansion. RNA-seq analysis showed that the expression of 37 *HvDJs* was salt-induced, and *HvDJB06*, *HvDJC58*, and *HvDJC59* were the most differentially expressed under salt stress. Protein–protein interaction analysis indicated that HvDJA09 and HvDJA05 play core roles in the complex regulatory network. Taken together, the current study provides valuable information for a deeper understanding of the function of *HvDJs* in regulating salt stress tolerance in barley.

## 1 Introduction

Soil salinity is a major abiotic stress, severely restricting crop growth and production. Salt stress can cause internal dehydration and disrupt metabolic processes (Munns, 2002). Correspondingly, plants have evolved multiple defense strategies to cope with salt stress, especially through diverse signal transduction pathways. It is well documented that transcription factors, protein kinases, and protein phosphatases are involved in a complex regulatory network in response to salt stress (Liang et al., 2024; Pieterse et al., 2012; Zhou et al., 2024). However, research exploring the impact of heat-shock proteins (Hsps) on salt tolerance remains quite limited.

J-domain proteins, also known as Hsp40s (Heat Shock Protein 40s), are characterized by the presence of an evolutionary conserved J-domain located near the N-terminus and composed of approximately 70 amino acid residues (Cyr et al., 1992). The invariant tripeptide (HPD) is the hallmark of the J-domain. The J-domain can interact with HSP70 and stimulate its ATPase activity to facilitate protein folding, unfolding, translocation, and degradation (Qiu et al., 2006; Wang et al., 2004). J-domain proteins consist of three domains: the highly conserved J-domain at the N-terminus, the CxxCxGxG zinc-finger domain (C, cysteine; G, glycine; X, other amino acid residues), and the C-terminal domain (Rosenzweig et al., 2019). JDP family proteins are usually classified into four categories based on their conserved domains: DJ-A, DJ-B, DJ-C, and DJ-D. DJ-A proteins are characterized by a J-domain, a CxxCxGxG zinc-finger domain, and a C-terminal domain. DJ-B proteins contain a J-domain plus either a zinc-finger domain or a C-terminal domain, whereas DJ-C proteins contain only a J-domain (Walsh et al., 2004). Additionally, DJ-D proteins contain a J-like domain that lacks the critical HPD tripeptide (Kampinga and Craig, 2010).

JDPs have been widely reported to be involved in resistance to biotic and abiotic stresses, such as pests, pathogens, drought, salt, low temperature, and heat. In *Arabidopsis*, DJA5 and DJA6 proteins are essential for chloroplast iron-sulfur cluster biogenesis (Zhang et al., 2021). In tomato, a chloroplast-targeted J-domain protein, LeCDJ1, can enhance heat tolerance and maintain the stability of photosystem II under chilling stress (Kong et al., 2014a, b). Overexpression of tobacco *MsDJLP* enhances chilling and heat tolerance (Lee et al., 2018). A putative J-domain protein in *Nicotiana tabacum* can facilitate drought tolerance by regulating the expression of drought-responsive genes (Xia et al., 2014). *AtJ3* can maintain pH homeostasis by directly interacting with PKS5, thereby enhancing salt and alkaline stress tolerance (Yang et al., 2010). Additionally, ERdjB has been reported to play a role in maintaining normal anther development in *Arabidopsis* under high temperatures (Yamamoto et al., 2020). In rice, OsDnaJ15 can facilitate the formation of the OsSUVH7–OsBAG4–OsMYB106 transcriptional complex to activate *OsHKT1;5* and enhance salt tolerance (Liu et al., 2023). These findings highlight the crucial roles of J-domain proteins in regulating tolerance to biotic and abiotic stresses.

Barley (*Hordeum vulgare*) is the fourth-largest cereal crop worldwide and is extensively used for human food, animal feed, and brewing material (Cai et al., 2020). Compared with other cereal crops (i.e., rice, wheat, maize), barley can withstand salt concentrations exceeding 200 mM, making it an ideal model crop for deciphering salt tolerant mechanisms (Fu et al., 2018; Munns and Tester, 2008). Previous studies have identified 129 JDP homologs in Arabidopsis (Zhang et al., 2018), 115 in rice (Luo et al., 2019), 76 in pepper (Fan et al., 2020), 236 in wheat (Liu et al., 2022), 86 in citrus (Tian et al., 2024), and 91 in maize (Li et al., 2024). However, limited studies has been conducted on the amount and functions of JDP genes in barley, despite its significantly higher salt tolerance compared to other cereal crops, including rice and wheat. Thus, it is imperative to determine the possible roles of *HvDJ* genes in response to salt stress.

In this study, we conducted a genome-wide analysis of the *JDP* gene family in barley and identified a total of 109 J-domain proteins. Phylogenetic relationships, gene structures, protein motifs, *cis*-regulatory elements, and chromosomal locations of these *HvDJs* were analyzed. We also found that tandem and segmental duplications extensively promoted the expansion of *HvDJs*. In addition, the expression profiles of *HvDJs* in response to salt stress were analyzed, identifying 37 salt-responsive *HvDJ* genes, including downregulated *HvDJC58* and *HvDJC59*, and upregulated *HvDJC46*. The protein structures of these three HvDJs were predicted using AlphaFold3. Finally, protein–protein interacting network identified hub *HvDJ* genes (*HvDJA09* and *HvDJA05*) within complex regulatory networks. These results highlight the biological functions of *HvDJ* in response to salt stress in barley.

## 2 Materials and methods

### 2.1 Genome-wide identification of *HvDJ* gene family in barley

The genomic sequences of barley were obtained from EnsemblPlants (http://plants.ensembl.org/index.html). The Hidden Markov Model (HMM) profile of the J-domain (PF00226), downloaded from the Pfam protein family database, was used as a query sequence to search for putative barley J-protein genes with an e-value < 1 × 10^−5^. The putative HvDJs were then verified using the NCBI Conserved Domain Database (https://www.ncbi.nlm.nih.gov/Structure/bwrpsb/bwrpsb.cgi), SMART (http://smart.embl-heidelberg.de/), and Pfam (https://pfam.xfam.org/). Finally, 109 genes were identified as members of the *JDP* gene family in barley. Furthermore, the amino acid lengths (aa), molecular weights (MW), and isoelectric points (pI) of the identified JDP proteins were analyzed using the ExPasy website (http://web.expasy.org/protparam/).

### 2.2 Phylogenetic analysis of HvDJ proteins

Multiple sequence alignment of the HvDJ protein sequences was conducted using MEGA7 software with the ClustalW algorithm (Kumar et al., 2016; Thompson et al., 1994). The aligned sequences were then subjected to phylogenetic analysis using the neighbor-joining (NJ) method through MEGA7 software with 1,000 bootstrap replicates.

### 2.3 Gene structure and conserved motif analysis of *HvDJ* genes

Gene structure features of the 109 HvDJs were extracted using TBtools software based on the barley gene feature format (GFF) files. In addition, conserved motifs were predicted and analyzed using the MEME online tool (http://meme-suite.org/tools/meme). The number of motifs was set to 10. TBtools was used to visualize the gene structure and MEME results (Chen et al., 2023).

### 2.4 *Cis*-elements analysis on the promoter region of *HvDJs*

Upstream 2-kb sequences of the 109 *HvDJ* genes were extracted from the barley genome database. The PlantCARE program (http://bioinformatics.psb.ugent.be/webtools/plantcare/html/) was used to analyze the sequences and identify putative *cis*-regulatory elements. TBtools was used to visualize the results (Chen et al., 2023).

### 2.5 Chromosomal distribution and gene duplication of *HvDJ* genes

*HvDJ* genes were mapped to barley chromosomes using TBtools based on barley genomic data (Chen et al., 2023). Tandem and segmental duplication events of the *HvDJ* genes, as well as genome collinearity between barley and other species (rice, maize, sorghum, wheat, and Arabidopsis), were analyzed using the Multiple Collinearity Scan ToolKit-X (MCScanX) with default parameters (Wang et al., 2012) and visualized using Circos and the Dual Synteny Plot in TBtools (Chen et al., 2023; Krzywinski et al., 2009). Nonsynonymous (Ka) and synonymous (Ks) substitution rates were calculated using the simple Ka/Ks calculator in TBtools (Chen et al., 2023).

### 2.6 Expression pattern analysis of *HvDJ* genes

Transcriptome data from salt-treated barley were obtained from published sources (Zhang et al., 2020). FastQC was used for quality control, and HISAT2 (v2.2.1) was then used to map clean reads to the reference barley genome (Morex) (Pertea et al., 2016). Transcriptome assembly was conducted using StringTie (v2.2.1). DESeq2 (v1.30.0) was used to identify differentially expressed genes (DEGs) based on a criteria two-fold change and an adjusted *p*-value <0.05. Heatmaps were generated using TBtools software (Chen et al., 2023).

### 2.7 qRT-PCR analysis

Four *JDP* genes identified as salt-induced in the RNA-seq data were selected for qRT-PCR validation. A barley cultivar “Golden Promise” was used as a plant material and treated with 100 mM NaCl at 0 h, 6 h, and 48 h from 14-day-old seedlings, following Shen et al. (2020). Total RNA waS extracted from barley roots using the Easy-Do Plant Total RNA Rapid Extraction Kit, and reverse transciption was performed using reverse transcriptase and universal oligo(dT) primers (9769 and RR037A, Takara). qRT-PCR reactions were prepared following the SYBR Green Supermix (RR820, Takara) protocol and run on a Roche LightCycler 480 II system. Primer sequences are listed in **Supplementary Table S6**, with the *α-tubulin* gene used as an internal reference. Relative expression levels were calculated using the 2^−ΔΔCT^ method.

### 2.8 Protein structure prediction and interaction network analysis of HvDJs

Protein structure prediction was performed using AlphaFold3 (Abramson et al., 2024). Protein–protein interactions (PPIs) of the HvDJ protein family were analyzed using STRING v12.0 (https://string-db.org). Cytoscape v3.10.0 (Shannon et al., 2003), was used to visualize the putative interaction network.

## 3 Results

### 3.1 Identification and characterization of *HvDJ*s

In total, 109 *JDP* genes were identified from the reference genome of the barley cultivar Morex (Mascher et al., 2017) (**Table 1**; **Supplementary Table S1**). Based on the J-domain and their chromosomal positions, they were classified into three types (DJA, DJB, and DJC), with each type harboring nine, eight, and 92 members, respectively (**Table 1**; **Supplementary Table S1**). Their protein lengths vary from 99 (HvDJC17) to 2,577 (HvDJC05) amino acids (aa), with molecular weights (MWs) ranging from 8.82 (HvDJC89) to 281.4 (HvDJC05) kDa (**Table 1**). In addition, the isoelectric points (pIs) of these HvDJ proteins ranged from 4.22 (HvDJC61) to 11.18 (HvDJC66) (**Table 1**). All HvDJs contained a J-domain consisting of an average of 61 aa, with HvDJC47 having the shortest J-domain (32 aa) and HvDJB07 the longest (105 aa) (**Supplementary Table S1**). The GRAVY values of all 109 J-domain proteins were below zero (except HvDJC60), indicating that these proteins are hydrophilic.

**Table 1:** *JDP* family genes identified in barley.

Gene name	Gene ID	Strand	Chr	Start	End	Length	MW	PI	GRAVY
						(aa)		(kDa)	
*HvDJA01*	*HORVU.MOREX.r3.4HG0395600.1*	−	4H	5.27E+08	527,149,468	536	58.88	9.63	−0.52
*HvDJA02*	*HORVU.MOREX.r3.5HG0454880.1*	+	5H	2.34E+08	234,509,793	426	45.94	8.39	−0.46
*HvDJA03*	*HORVU.MOREX.r3.5HG0456050.1*	+	5H	2.45E+08	245174,164	537	57.52	10.22	−0.46
*HvDJA04*	*HORVU.MOREX.r3.5HG0507940.1*	−	5H	5.2E+08	520,373,657	422	46.42	6.44	−0.75
*HvDJA05*	*HORVU.MOREX.r3.5HG0517090.1*	+	5H	5.43E+08	542,796,541	421	46.81	5.72	−0.77
*HvDJA06*	*HORVU.MOREX.r3.6HG0600860.1*	−	6H	4.12E+08	412,485,687	425	47.05	7.09	−0.80
*HvDJA07*	*HORVU.MOREX.r3.6HG0626860.1*	−	6H	5.45E+08	545,420,552	490	52.07	9.67	−0.36
*HvDJA08*	*HORVU.MOREX.r3.7HG0641550.1*	+	7H	13,514,729	13,520,110	444	47.87	9.37	−0.56
*HvDJA09*	*HORVU.MOREX.r3.7HG0664630.1*	−	7H	82,017,762	82,027,496	479	53.16	9.22	−0.44
*HvDJB01*	*HORVU.MOREX.r3.1HG0015480.1*	−	1H	43,297,593	43,302,516	344	36.68	9.04	−0.41
*HvDJB02*	*HORVU.MOREX.r3.1HG0066870.1*	+	1H	4.36E+08	436,047,355	329	36.51	10.25	−0.61
*HvDJB03*	*HORVU.MOREX.r3.1HG0087430.1*	−	1H	4.99E+08	499,320,445	369	39.37	9.42	−0.50
*HvDJB04*	*HORVU.MOREX.r3.3HG0250320.1*	+	3H	1.48E+08	148,013,275	346	37.65	9.56	−0.50
*HvDJB05*	*HORVU.MOREX.r3.3HG0298330.1*	−	3H	5.33E+08	533,221,347	324	35.70	9.74	−0.50
*HvDJB06*	*HORVU.MOREX.r3.3HG0327050.1*	+	3H	6.12E+08	611,772,084	322	35.86	5.56	−0.63
*HvDJB07*	*HORVU.MOREX.r3.6HG0553140.1*	+	6H	35,488,193	35,492,374	337	37.84	9.61	−0.79
*HvDJB08*	*HORVU.MOREX.r3.7HG0698270.1*	−	7H	3.68E+08	367,934,809	346	38.43	9.55	−0.78
*HvDJC01*	*HORVU.MOREX.r3.1HG0003150.1*	−	1H	6,145,972	6,148,385	235	26.18	10.03	−1.06
*HvDJC02*	*HORVU.MOREX.r3.1HG0009210.1*	+	1H	22,084,321	22,088,312	1198	132.80	7.42	−0.75
*HvDJC03*	*HORVU.MOREX.r3.1HG0024210.1*	+	1H	95,939,337	95,943,942	158	17.39	4.79	−0.55
*HvDJC04*	*HORVU.MOREX.r3.1HG0049180.1*	−	1H	3.25E+08	325,003,202	540	60.03	7.85	−0.43
*HvDJC05*	*HORVU.MOREX.r3.1HG0053190.1*	−	1H	3.55E+08	355,275,474	2577	281.36	6.18	−0.09
*HvDJC06*	*HORVU.MOREX.r3.1HG0059600.1*	−	1H	3.97E+08	396,901,467	1476	158.19	6.66	−0.60
*HvDJC07*	*HORVU.MOREX.r3.1HG0060530.1*	+	1H	4.03E+08	402,710,525	366	42.53	9.71	−0.90
*HvDJC08*	*HORVU.MOREX.r3.1HG0061870.1*	−	1H	4.1E+08	409,600,443	303	33.01	9.96	−0.76
*HvDJC09*	*HORVU.MOREX.r3.1HG0072560.1*	−	1H	4.59E+08	458,650,889	147	15.78	4.60	−0.68
*HvDJC10*	*HORVU.MOREX.r3.1HG0079660.1*	+	1H	4.84E+08	484,406,597	316	35.20	7.17	−0.44
*HvDJC11*	*HORVU.MOREX.r3.1HG0082120.1*	+	1H	4.9E+08	489,953,801	347	38.76	6.75	−0.51
*HvDJC12*	*HORVU.MOREX.r3.1HG0092010.1*	+	1H	5.08E+08	508,064,718	1504	166.07	5.27	−0.97
*HvDJC13*	*HORVU.MOREX.r3.1HG0092140.1*	+	1H	5.08E+08	508,326,136	667	74.82	8.94	−0.92
*HvDJC14*	*HORVU.MOREX.r3.2HG0121120.1*	−	2H	75,249,952	75,251,326	130	14.60	10.36	−0.42
*HvDJC15*	*HORVU.MOREX.r3.2HG0123170.1*	−	2H	86,237,470	86,240,937	269	30.96	9.36	−0.80
*HvDJC16*	*HORVU.MOREX.r3.2HG0124170.1*	−	2H	94,047,035	94,049,971	389	40.63	5.77	−0.33
*HvDJC17*	*HORVU.MOREX.r3.2HG0144020.1*	−	2H	2.45E+08	244,560,166	99	11.81	4.67	−0.37
*HvDJC18*	*HORVU.MOREX.r3.2HG0154370.1*	+	2H	3.66E+08	366,190,859	237	28.40	9.99	−1.33
*HvDJC19*	*HORVU.MOREX.r3.2HG0159340.1*	+	2H	4.07E+08	407,037,946	681	76.50	5.70	−0.23
*HvDJC20*	*HORVU.MOREX.r3.2HG0162940.1*	+	2H	4.35E+08	434,509,344	733	82.19	8.35	−0.68
*HvDJC21*	*HORVU.MOREX.r3.2HG0202070.1*	+	2H	6.28E+08	628,333,074	593	66.15	9.84	−1.08
*HvDJC22*	*HORVU.MOREX.r3.2HG0210480.1*	−	2H	6.48E+08	647,997,658	482	51.87	8.99	−0.34
*HvDJC23*	*HORVU.MOREX.r3.2HG0214910.1*	+	2H	6.57E+08	657,412,828	271	30.87	10.21	−0.98
*HvDJC24*	*HORVU.MOREX.r3.3HG0218100.1*	−	3H	71,149	76,309	297	34.43	9.33	−0.45
*HvDJC25*	*HORVU.MOREX.r3.3HG0232160.1*	−	3H	28,510,353	28,512,444	200	21.92	10.66	−0.84
*HvDJC26*	*HORVU.MOREX.r3.3HG0242780.1*	−	3H	85,733,732	85,737,465	112	12.06	10.95	−0.22
*HvDJC27*	*HORVU.MOREX.r3.3HG0256610.1*	+	3H	1.98E+08	198,244,290	1103	119.84	8.01	−0.43
*HvDJC28*	*HORVU.MOREX.r3.3HG0257630.1*	−	3H	2.1E+08	209,721,737	245	26.82	11.01	−0.57
*HvDJC29*	*HORVU.MOREX.r3.3HG0257660.1*	−	3H	2.1E+08	210,210,972	169	19.07	9.97	−0.81
*HvDJC30*	*HORVU.MOREX.r3.3HG0260160.1*	−	3H	2.4E+08	239,938,328	445	49.81	8.04	−0.61
*HvDJC31*	*HORVU.MOREX.r3.3HG0266820.1*	+	3H	3.2E+08	320,227,701	385	43.13	8.75	−0.73
*HvDJC32*	*HORVU.MOREX.r3.3HG0269430.1*	−	3H	3.45E+08	345,398,592	1131	124.66	8.23	−0.65
*HvDJC33*	*HORVU.MOREX.r3.3HG0270430.1*	+	3H	3.55E+08	355,403,286	190	20.90	5.56	−0.57
*HvDJC34*	*HORVU.MOREX.r3.3HG0274180.1*	−	3H	3.87E+08	387,233,923	1608	179.02	4.70	−0.91
*HvDJC35*	*HORVU.MOREX.r3.3HG0283210.1*	+	3H	4.53E+08	452,540,924	337	37.80	6.65	−0.57
*HvDJC36*	*HORVU.MOREX.r3.3HG0286640.1*	+	3H	4.72E+08	472,502,435	402	44.99	8.98	−0.68
*HvDJC37*	*HORVU.MOREX.r3.3HG0311380.1*	+	3H	5.77E+08	577,389,558	728	81.93	9.43	−0.83
*HvDJC38*	*HORVU.MOREX.r3.3HG0312260.1*	−	3H	5.8E+08	580,010,196	748	84.25	5.34	−0.41
*HvDJC39*	*HORVU.MOREX.r3.3HG0330450.1*	−	3H	6.19E+08	619,261,982	762	85.10	8.75	−0.71
*HvDJC40*	*HORVU.MOREX.r3.3HG0331030.1*	−	3H	6.21E+08	620,932,373	461	51.58	6.69	−0.49
*HvDJC41*	*HORVU.MOREX.r3.4HG0339210.1*	+	4H	25,446,071	25,449,997	237	27.46	9.86	−1.22
*HvDJC42*	*HORVU.MOREX.r3.4HG0345490.1*	+	4H	60,920,222	60,921,300	240	27.20	9.23	−0.65
*HvDJC43*	*HORVU.MOREX.r3.4HG0346750.1*	−	4H	70,260,778	70,263,456	892	100.79	6.42	−0.56
*HvDJC44*	*HORVU.MOREX.r3.4HG0363280.1*	+	4H	2.16E+08	216,479,866	931	102.21	5.48	−0.93
*HvDJC45*	*HORVU.MOREX.r3.4HG0370060.1*	−	4H	2.98E+08	298,224,914	398	42.12	6.40	−0.34
*HvDJC46*	*HORVU.MOREX.r3.4HG0380120.1*	+	4H	4.1E+08	40,960,0776	148	16.08	10.86	−0.52
*HvDJC47*	*HORVU.MOREX.r3.4HG0382910.1*	+	4H	4.34E+08	433,525,364	593	63.79	9.92	−0.11
*HvDJC48*	*HORVU.MOREX.r3.4HG0383510.1*	+	4H	4.4E+08	439,957,451	180	19.14	5.98	−0.70
*HvDJC49*	*HORVU.MOREX.r3.4HG0384590.1*	−	4H	4.5E+08	449,613,424	576	62.97	9.90	−0.46
*HvDJC50*	*HORVU.MOREX.r3.4HG0390430.1*	−	4H	4.91E+08	490,799,057	246	29.28	9.86	−1.26
*HvDJC51*	*HORVU.MOREX.r3.4HG0392440.1*	+	4H	5.05E+08	505,253,960	173	18.58	11.18	−0.35
*HvDJC52*	*HORVU.MOREX.r3.4HG0399320.1*	−	4H	5.48E+08	547,603,366	638	69.92	8.07	−0.90
*HvDJC53*	*HORVU.MOREX.r3.4HG0411040.1*	−	4H	5.9E+08	590,473,467	765	85.37	8.67	−0.70
*HvDJC54*	*HORVU.MOREX.r3.5HG0429600.1*	+	5H	25,201,948	25,208,042	545	61.38	8.65	−0.14
*HvDJC55*	*HORVU.MOREX.r3.5HG0444800.1*	+	5H	1.15E+08	115,451,938	906	100.18	6.29	−1.04
*HvDJC56*	*HORVU.MOREX.r3.5HG0448940.1*	−	5H	1.53E+08	152,840,255	603	68.09	5.11	−1.08
*HvDJC57*	*HORVU.MOREX.r3.5HG0457230.1*	−	5H	2.57E+08	257,375,893	228	24.64	5.17	−0.24
*HvDJC58*	*HORVU.MOREX.r3.5HG0476120.1*	−	5H	4.06E+08	405,974,150	154	17.18	10.58	−0.74
*HvDJC59*	*HORVU.MOREX.r3.5HG0476180.1*	−	5H	4.06E+08	406,109,820	167	18.11	10.28	−0.60
*HvDJC60*	*HORVU.MOREX.r3.5HG0478010.1*	+	5H	4.16E+08	415,847,057	268	29.84	9.93	0.10
*HvDJC61*	*HORVU.MOREX.r3.5HG0486810.1*	+	5H	4.62E+08	461,952,388	173	18.96	4.22	−0.20
*HvDJC62*	*HORVU.MOREX.r3.5HG0487540.1*	+	5H	4.65E+08	465,200,268	546	58.03	10.03	−0.48
*HvDJC63*	*HORVU.MOREX.r3.5HG0491660.1*	−	5H	4.81E+08	481,150,560	394	43.95	6.29	−0.48
*HvDJC64*	*HORVU.MOREX.r3.5HG0511130.1*	−	5H	5.28E+08	527,900,282	581	64.47	4.68	−0.97
*HvDJC65*	*HORVU.MOREX.r3.5HG0512520.1*	+	5H	5.32E+08	531,564,547	505	56.82	7.30	−0.37
*HvDJC66*	*HORVU.MOREX.r3.5HG0514290.1*	+	5H	5.37E+08	536,912,167	111	12.18	11.08	−0.17
*HvDJC67*	*HORVU.MOREX.r3.5HG0527190.1*	+	5H	5.65E+08	565,006,278	264	28.79	5.74	−0.73
*HvDJC68*	*HORVU.MOREX.r3.5HG0527200.1*	−	5H	5.65E+08	565,022,363	233	25.65	9.28	−0.17
*HvDJC69*	*HORVU.MOREX.r3.5HG0527220.1*	−	5H	5.65E+08	565,063,756	179	20.36	8.77	−0.48
*HvDJC70*	*HORVU.MOREX.r3.5HG0529410.1*	−	5H	5.7E+08	570,081,755	716	78.89	9.71	−0.48
*HvDJC71*	*HORVU.MOREX.r3.5HG0530640.1*	+	5H	5.73E+08	572,828,412	282	31.18	9.89	−0.77
*HvDJC72*	*HORVU.MOREX.r3.5HG0530650.1*	+	5H	5.73E+08	572,840,053	312	33.03	6.91	−0.66
*HvDJC73*	*HORVU.MOREX.r3.6HG0573240.1*	−	6H	1.49E+08	148,561,739	281	32.27	7.44	−1.01
*HvDJC74*	*HORVU.MOREX.r3.6HG0573670.1*	+	6H	1.52E+08	152,176,697	975	108.67	6.25	−0.64
*HvDJC75*	*HORVU.MOREX.r3.6HG0605620.1*	−	6H	4.52E+08	452,277,119	121	14.32	5.57	−0.90
*HvDJC76*	*HORVU.MOREX.r3.6HG0615820.1*	−	6H	5.1E+08	509,829,520	441	49.33	8.42	−0.72
*HvDJC77*	*HORVU.MOREX.r3.6HG0616590.1*	+	6H	5.13E+08	513,006,671	131	15.43	4.60	−0.81
*HvDJC78*	*HORVU.MOREX.r3.6HG0621190.1*	−	6H	5.3E+08	529,815,253	265	28.60	5.19	−0.85
*HvDJC79*	*HORVU.MOREX.r3.7HG0661570.1*	+	7H	67,224,068	67,225,954	237	25.90	6.88	−0.75
*HvDJC80*	*HORVU.MOREX.r3.7HG0667730.1*	−	7H	1.01E+08	100,792,110	437	49.44	8.18	−0.70
*HvDJC81*	*HORVU.MOREX.r3.7HG0672240.1*	+	7H	1.25E+08	125,447,097	259	29.07	7.99	−0.61
*HvDJC82*	*HORVU.MOREX.r3.7HG0674400.1*	−	7H	1.37E+08	136,729,981	111	11.87	9.91	−0.30
*HvDJC83*	*HORVU.MOREX.r3.7HG0677370.1*	−	7H	1.57E+08	157,347,245	303	33.78	8.12	−0.76
*HvDJC84*	*HORVU.MOREX.r3.7HG0679310.1*	+	7H	1.71E+08	170,965,584	1034	115.66	7.00	−0.49
*HvDJC85*	*HORVU.MOREX.r3.7HG0681040.1*	−	7H	1.84E+08	184,254,504	394	43.71	4.93	−0.48
*HvDJC86*	*HORVU.MOREX.r3.7HG0687460.1*	+	7H	2.36E+08	236,261,883	141	15.19	9.93	−0.33
*HvDJC87*	*HORVU.MOREX.r3.7HG0701210.1*	+	7H	3.95E+08	395,042,965	379	42.60	5.78	−0.59
*HvDJC88*	*HORVU.MOREX.r3.7HG0701620.1*	−	7H	4E+08	399,772,402	182	20.21	9.97	−0.63
*HvDJC89*	*HORVU.MOREX.r3.7HG0732770.1*	−	7H	5.85E+08	585,423,146	2577	8.82	10.47	−0.62
*HvDJC90*	*HORVU.MOREX.r3.7HG0742700.1*	+	7H	6.1E+08	609,897,473	159	17.84	6.68	−0.75
*HvDJC91*	*HORVU.MOREX.r3.7HG0742800.1*	−	7H	6.1E+08	610,256,200	159	17.83	6.68	−0.72
*HvDJC92*	*HORVU.MOREX.r3.7HG0746680.1*	+	7H	6.18E+08	6180,44,498	140	14.88	9.71	−0.31

GRAVY, grand average of hydrophobicity.

### 3.2 Structure and motif analysis of *HvDJs*

**Figure 1:**
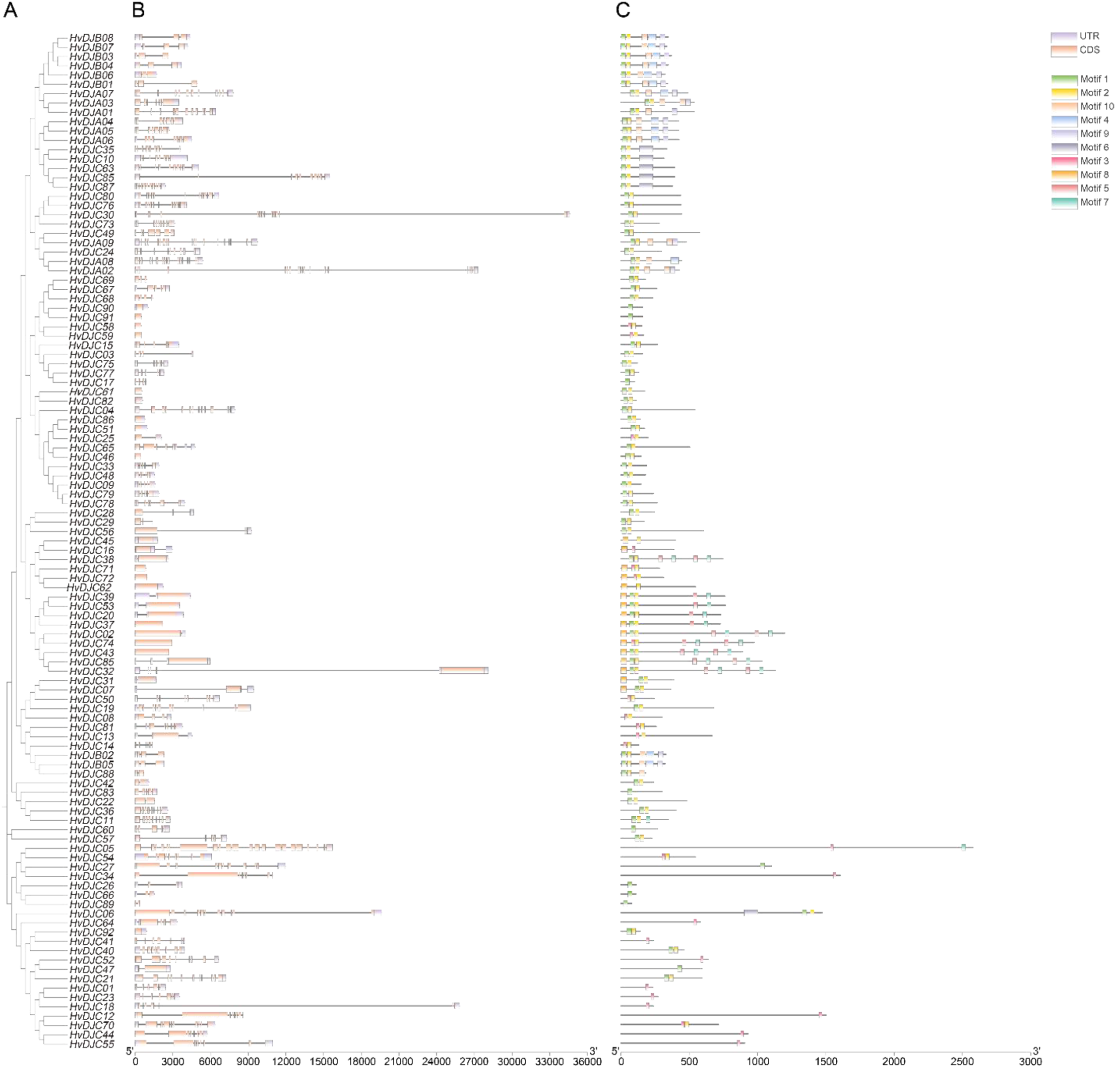
The phylogenetic relationship, gene structure, and motif compositions of *HvDJs*. **(A)** The phylogenetic tree was constructed using the full-length sequences of HvDJ proteins. **(B)** Purple rectangles, orange rectangles, and black lines indicate UTRs (untranslated region), CDSs (coding sequence or exons), and introns, respectively **(C)** Ten amino acid motifs in HvDJ proteins are shown in different colored boxes, and black lines indicate amino acid length.

We further analyzed the conserved motifs and gene structures of HvDJ protein sequences, followed by a phylogenetic tree analysis for HvDJs (**Figure 1A**). Gene structure analysis showed that *HvDJs* harbored 1–22 exons and 0–21 introns (**Figure 1B**). In details, among 109 *HvDJ* genes, 17 had no introns, and the others contained two to 22 exons (13 with two exons, 16 with three, five with four, 12 with five, six with six, six with seven, eight with eight, seven with nine, five with 10, seven with 11, two with 12, one each with 13, 17, 18, 19, and 22 exons). Ten conserved motifs were identified in 109 HvDJ protein sequences (**Figure 1B**; **Supplementary Table S2**). Among these motifs, motif1 and motif2 were the most frequently present, appearing 82 and 87 times, respectively, indicating that these two motifs were highly conserved in the core *JDP* genes (**Figure 1B**).

### 3.3 *Cis*-elements analysis of *HvDJs*

**Figure 2:**
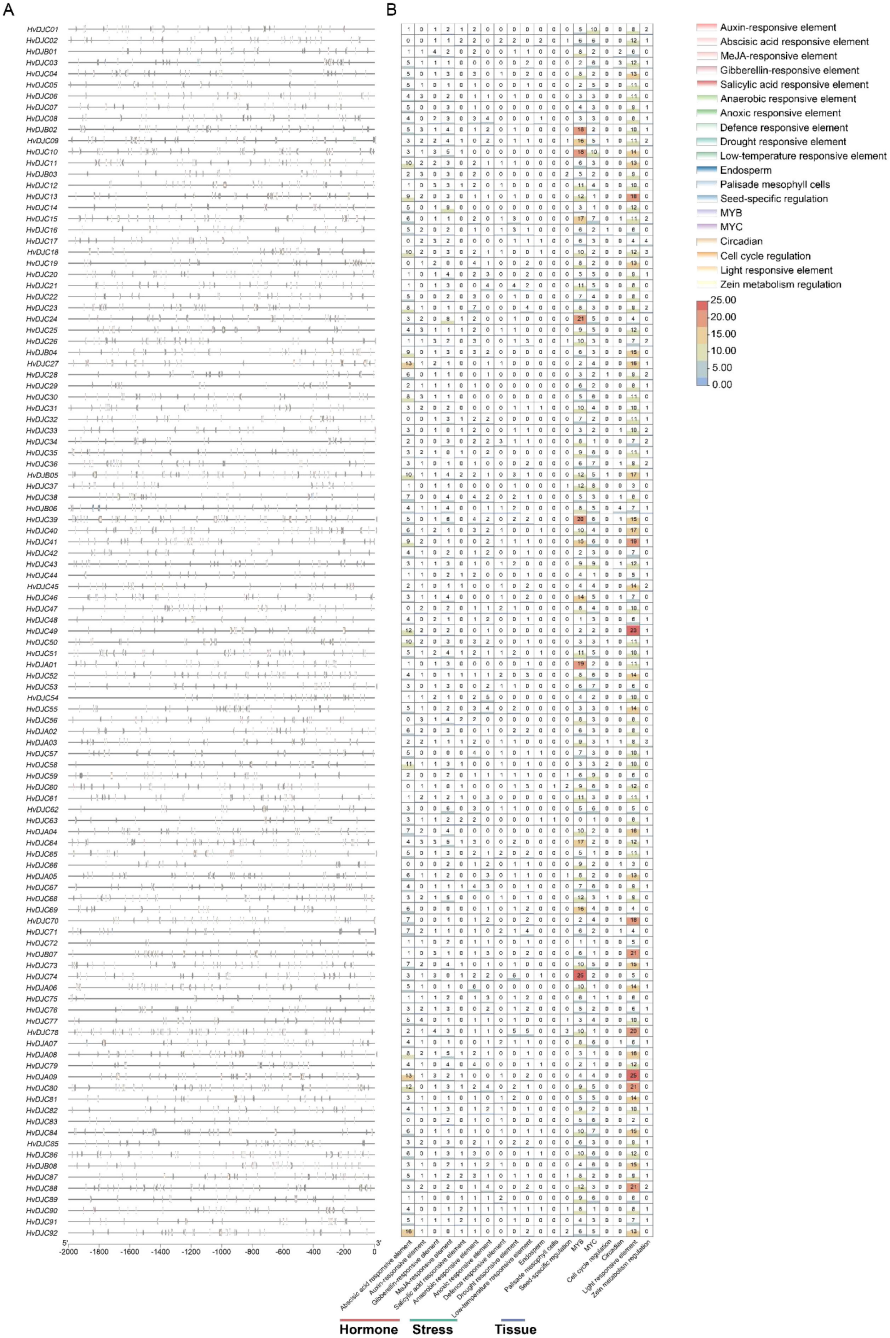
The *cis*-regulatory elements predicted in the promoter regions of *HvDJs*. **(A)** Distribution of predicted *cis*-regulatory elements in the *HvDJ* gene family. **(B)** The number of each *cis*-regulatory element in the *HvDJ* gene family.

To understand the transcriptional regulation of *HvDJ* genes, we analyzed the *cis*-elements in the promoter regions of the *HvDJs.* A total of 19 types of *cis*-regulatory elements were identified in the upstream 2,000-bp sequences of 109 *HvDJs* (**Figure 2**; **Supplementary Table S3**). These elements are involved in hormone (auxin, abscisic acid, methyl jasmonate, gibberellin, and salicylic acid), stress (anaerobic, anoxic, defense, drought, and low temperature), tissue (endosperm, palisade mesophyll cells, and seed), circadian rhythm, cell cycle, light, zein, as well as transcription factor binding sites (MYB, MYC) (**Figure 2**). Notably, most *HvDJs* harbored MYB- and light-responsive *cis*-elements, indicating that *HvDJs* may be regulated by MYB transcription factors and light signals.

### 3.4 Chromosomal distribution and gene duplication analysis of *HvDJs*

**Figure 3:**
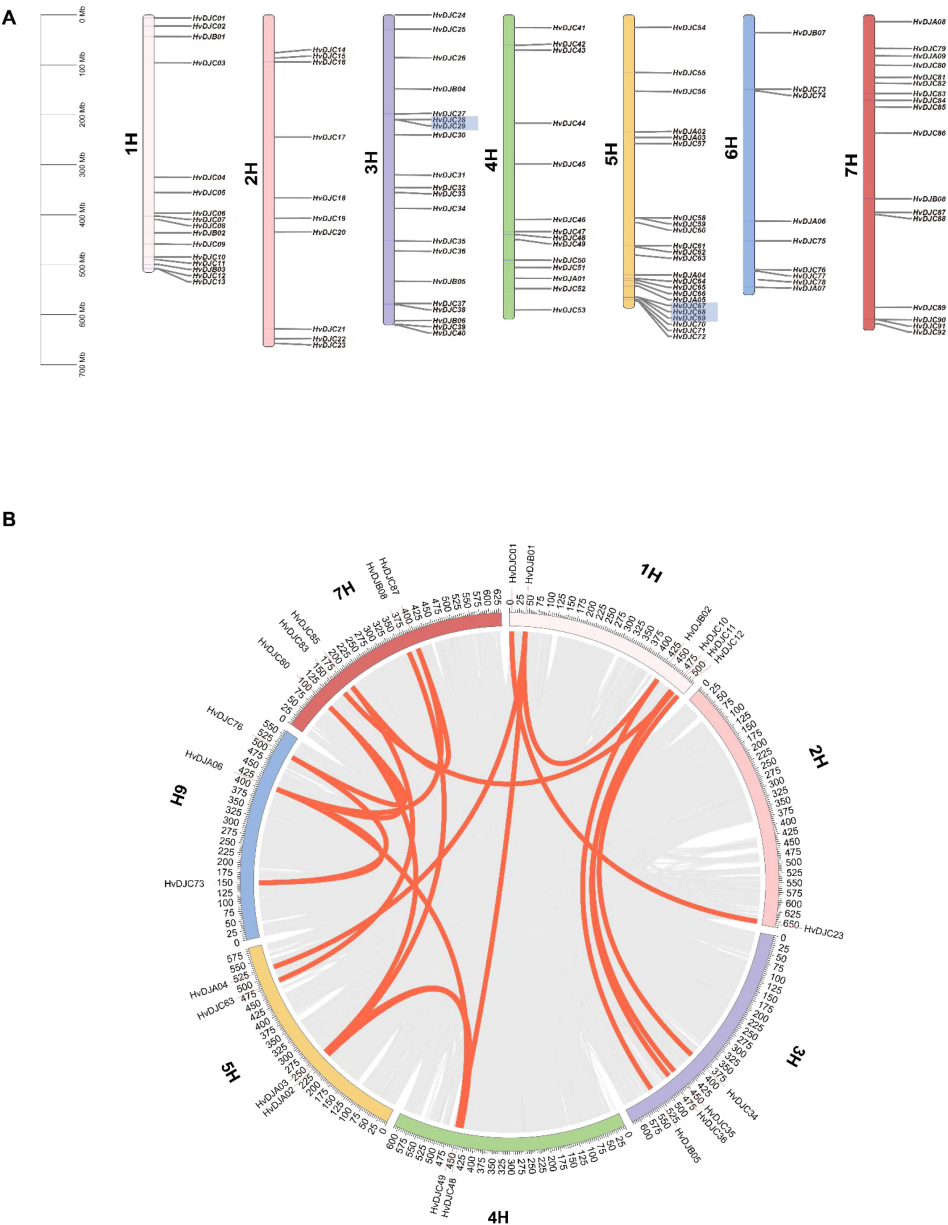
**(A)** The distribution of *HvDJ* genes across the chromosomes. 1–7H represent the seven barley chromosomes; shaded areas under gene names represent tandem duplications, and the 0 Mb–700 Mb scale indicates chromosome length. **(B)** Synteny analysis of *HvDJ* genes in the barley genome. Gray lines represent all synteny blocks in the barley genome. Orange lines represent duplicated *HvDJ* gene pairs.

The 109 *HvDJ* genes were unevenly distributed on the seven chromosomes (**Figure 3A**; **Table 1**), with 1H, 2H, 3H, 4H, 5H, 6H, and 7H containing 16, 10, 20, 14, 23, 9, and 17 *HvDJ* genes, respectively. Additionally, we identified three pairs of tandemly duplicated *HvDJ* genes—*HvDJC28* and *HvDJC29*, *HvDJC67* and *HvDJC68*, and *HvDJC68* and *HvDJC69* (**Figure 3A**). These genes were closely distributed on the chromosomes and formed clusters on the phylogenetic tree. Segmental duplication analysis of the 109 *HvDJ* genes identified 21 pairs of segmental duplication events (**Figure 3B**). The ratios of non-synonymous (Ka) to synonymous (Ks) substitutions (Ka/Ks) in these two tandem duplication and 10 segmental duplication gene pairs were less than 1 (**Supplementary Table S4**), indicating that purifying selection is likely stronger than positive selection in the evolution of the *HvDJ* genes.

### 3.5 Synteny analysis of *JDP* genes

**Figure 4:**
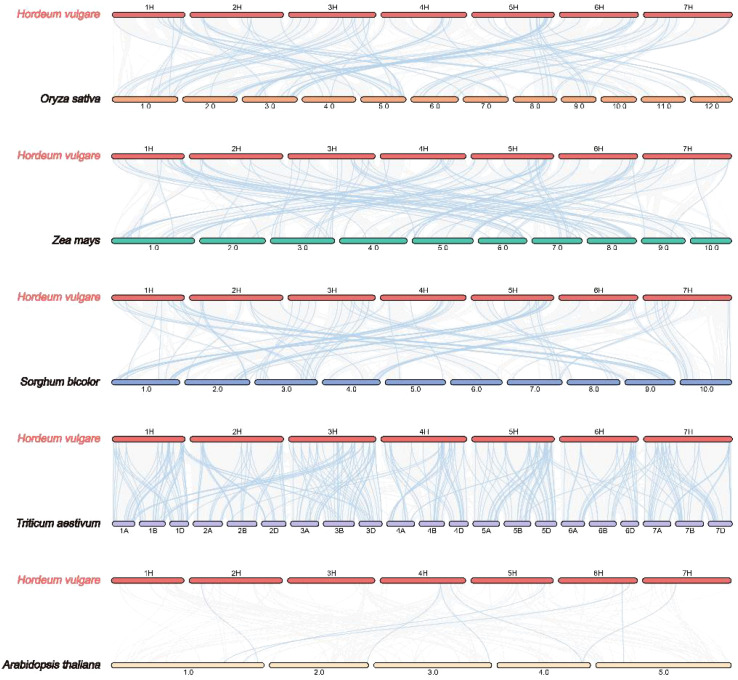
Synteny analysis of JDP genes between barley and six other plants species (*Oryza sativa*, *Zea mays*, *Sorghum bicolor*, *Triticum aestivum*, and *Arabidopsis thaliana*). Gray lines between barley and the other species represent collinear blocks across broad genomic regions, while colored lines indicate the synteny of *JDP* genes.

To determine the evolutionary trajectory of the *JDP* family in barley and other plant species, we performed an evolutionary relationship analysis of *JDP* genes. In detail, we compared four monocotyledonous species (rice, maize, sorghum, and wheat) and one dicotyledonous plant (*Arabidopsis*) (**Figure 4**). The results showed that barley shared 88, 101, 90, 280, and eight collinear genes with rice, maize, sorghum, wheat, and *Arabidopsis*, respectively, indicating that *JDPs* in barley are more closely related to these in wheat in terms of evolution relationship.

### 3.6 Expression profiles of *HvDJs* in response to salt stress

**Figure 5:**
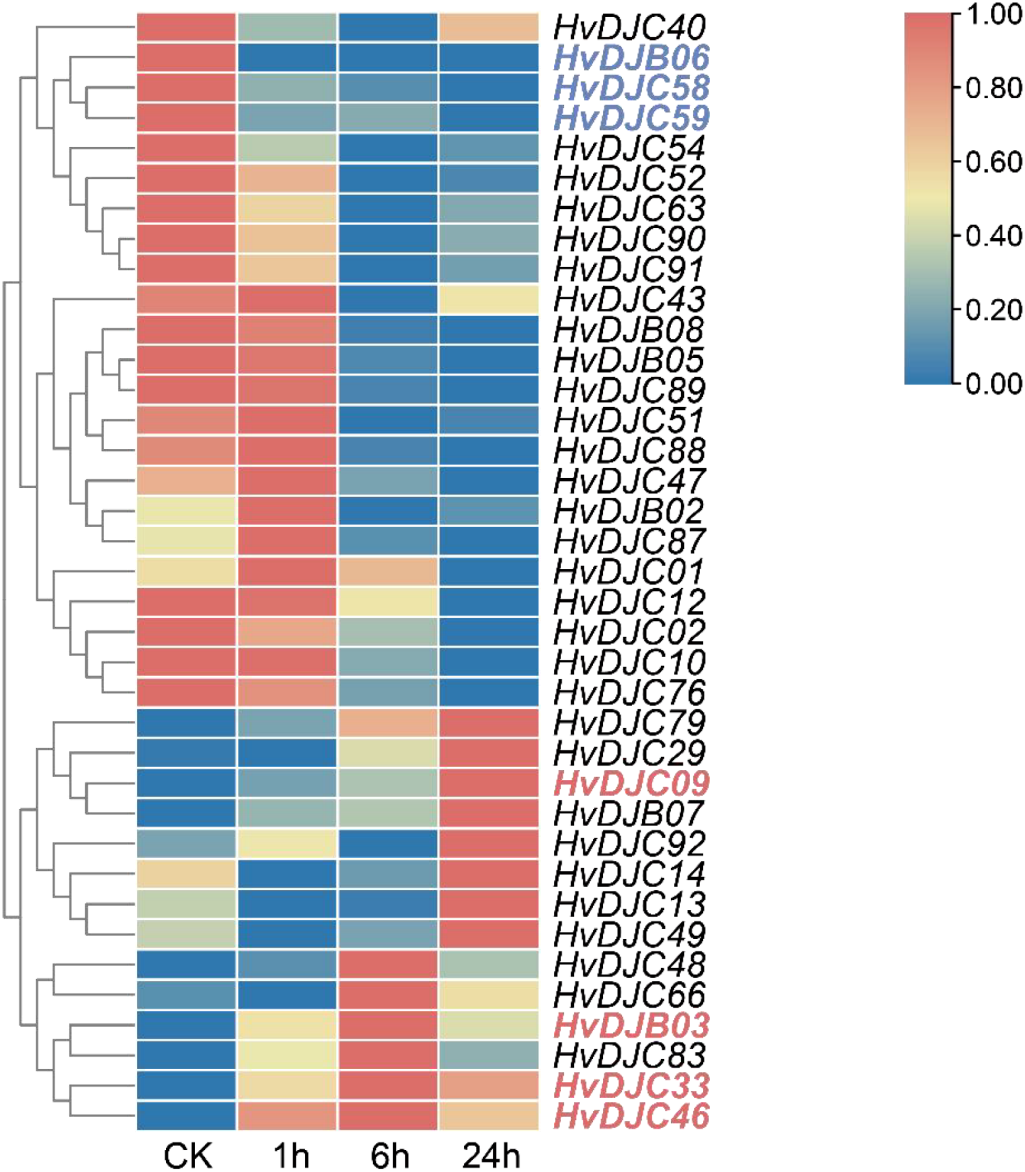
Expression profiles of *HvDJ* genes in response to salt stress. TPM values of *HvDJs* genes are scaled individually from 0 to 1. Blue and red represent lower and higher expression levels, respectively.

**Figure 6:**
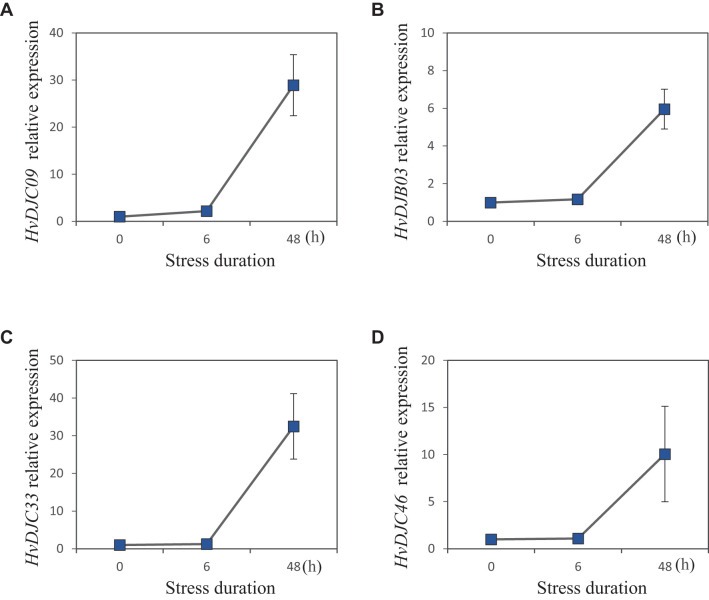
Expression patterns of four selected JDP genes in barley under 200 mM salt stress at 0h, 6h, and 48h. **(A)** HvDJC09, **(B)** HvDJB03, **(C)** HvDJC33, and **(D)** HvDJC46 (n =4,±SE).

To explore the response of *HvDJ* genes to salt stress, we investigated their expression using public data (Zhang et al., 2020). Barley seedlings were sampled at four time points (0 h, 1 h, 6 h, and 24 h) of salt exposure for RNA-seq analysis. Differentially expressed genes (DEGs) were identified by comparing salt-stressed samples to the control. In total, we identified 37 salt-responsive *HvDJs* (**Figure 5**; **Supplementary Table S5**). Among them, *HvDJC09*, *HvDJB03*, *HvDJC33*, *HvDJB06*, *HvDJC46*, *HvDJC58*, and *HvDJC59* were differentially expressed at 1 h, 6 h, and 24 h after salt stress. Four genes—*HvDJC09*, *HvDJB03*, *HvDJC33*, and *HvDJC46*—were significantly upregulated in response to salt stress, with *HvDJC46* being the most upregulated, while *HvDJB06*, *HvDJC58*, and *HvDJC59* were significantly downregulated, with *HvDJB06* exhibiting the greatest downregulation. To further validate their response patterns under short-term salt stress, four upregulated *HvDJ* genes (*HvDJC09*, *HvDJB03*, *HvDJC33*, and *HvDJC46*) were selected for detailed qRT-PCR analysis (**Figure 6**). The four HvDJs exhibited upregulated expression patterns under salt stress, with the highest expression at 48 h (**Figure 6**).

**Figure 7:**
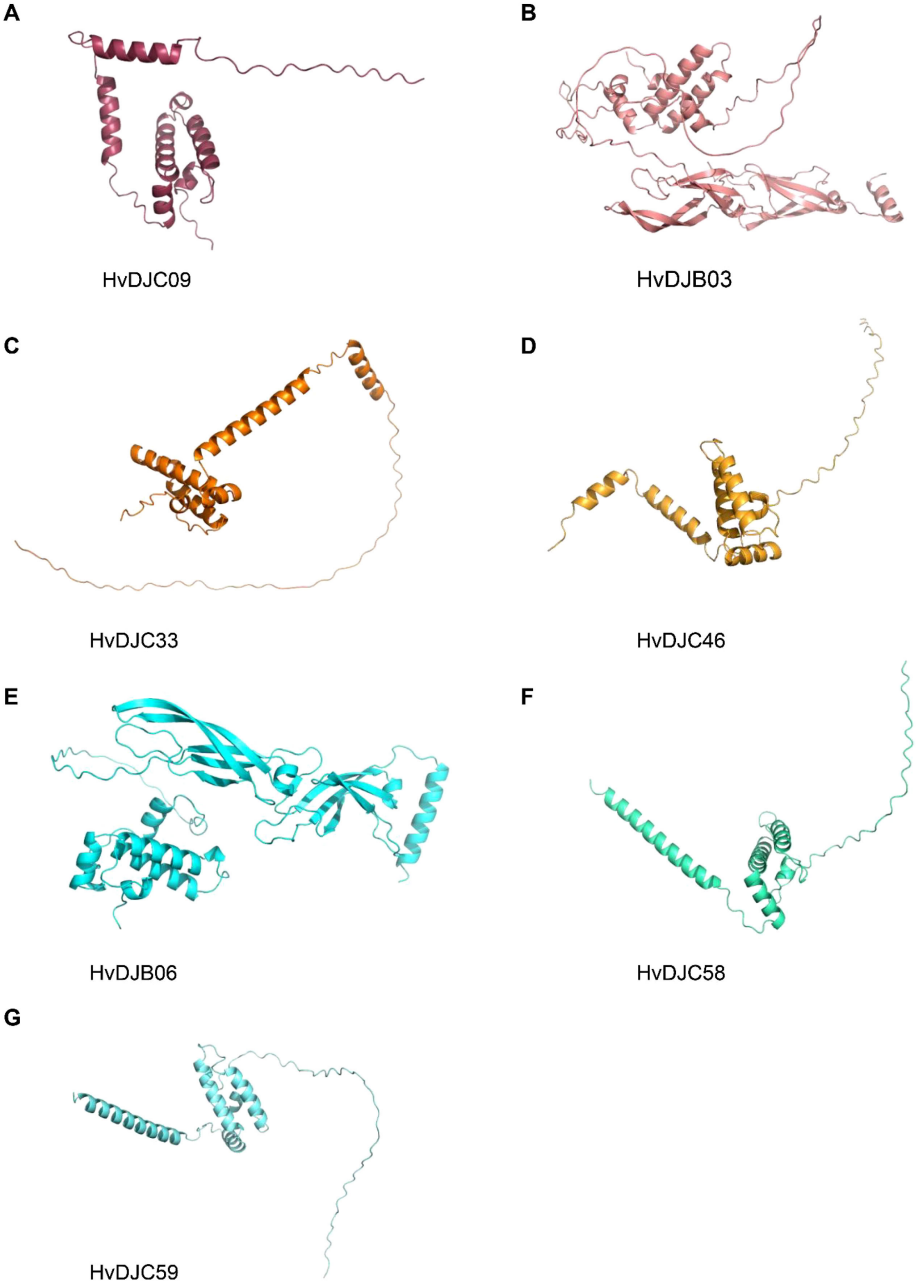
Schematic structures of HvDJC09, HvDJB03, HvDJC33, HvDJC46, HvDJB06, HvDJC58, and HvDJC59 predicted by AlphaFold3.

To structurally analyze these salt-responsive JDPs, we attempted to identify key similarities and differences in their three-dimensional conformations, aiming to provide a structural basis for their functional characterization. We analyzed the protein structure of HvDJC09, HvDJB03, HvDJC33, HvDJC46, HvDJB06, HvDJC58, and HvDJC59 using AlphaFold3 (**Figure 7**). Interestingly, we observed highly similar protein structures among the five proteins (HvDJC09, HvDJC33, HvDJC46, HvDJC58, and HvDJC59), all harboring at least four α-helices (**Figure 7**). Meanwhile, we found that the a-helices of HvDJC46, HvDJC58, and HvDJC59 were unevenly distributed at the C- terminal (**Figures 7D, F, G**), whereas those of HvDJC09 and HvDJC33 were localized at the N-terminus (**Figures 7A, C**). HvDJB03 and HvDJB06 were predicted to harbor similar protein structures, with both α-helices and β-sheets unevenly distributed (**Figures 7B, E**).

### 3.7 Regulatory network analysis of *HvDJ* genes

**Figure 8:**
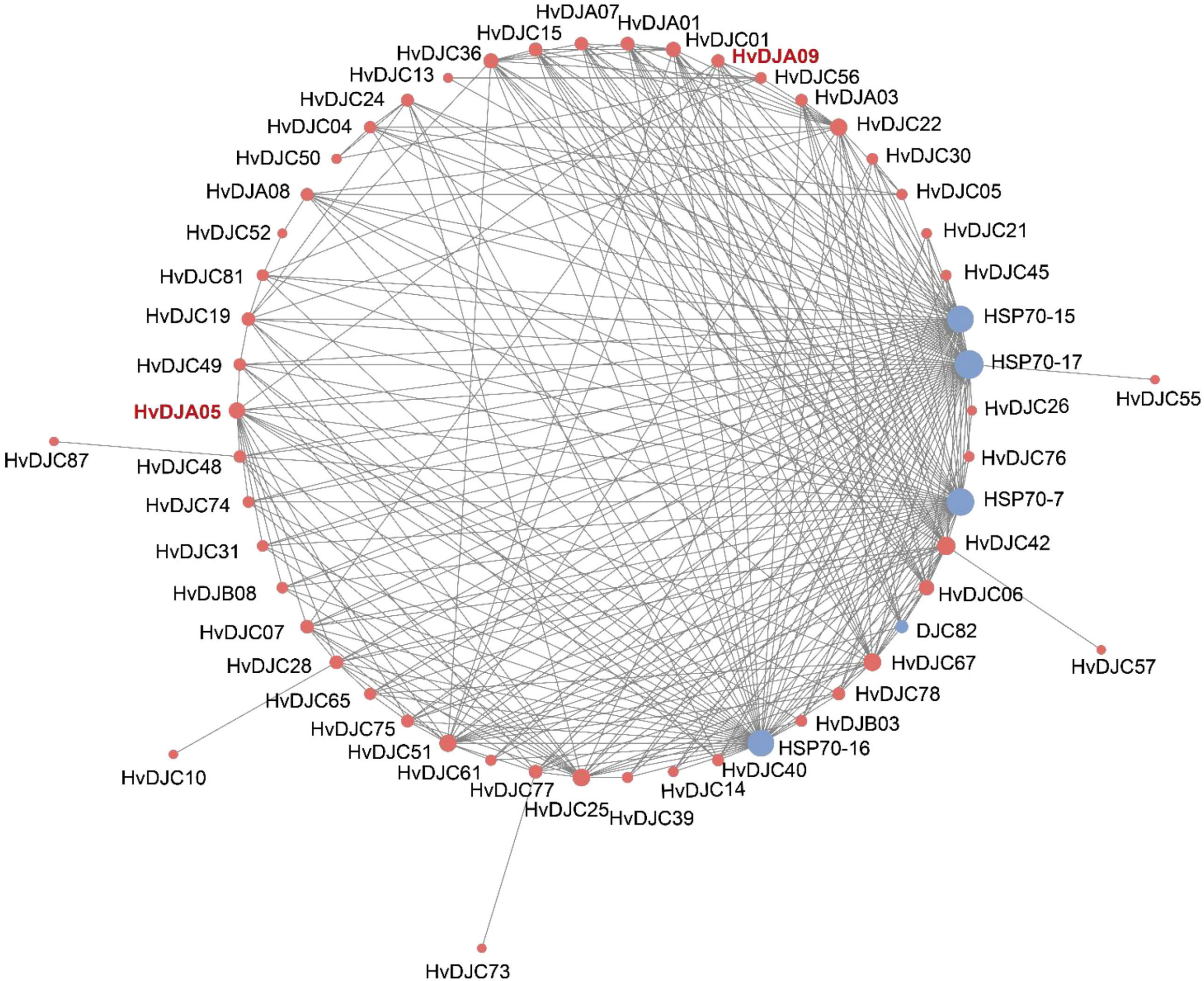
Protein–protein interaction networks of HvDJ proteins. Red indicates HvDJs and blue indicates other interacting proteins.

STRING integrates both experimental and computational evidence, including high-throughput experimental data (e.g., yeast two-hybrid, affinity purification-mass spectrometry), computational predictions (e.g., gene co-expression, conserved genomic context, phylogenetic profiling), text-mining of published literature, and database-curated interactions from known pathways. To decipher the molecular regulatory networks of HvDJs, we used the STRING database to predict potential interactions among the HvDJ proteins (**Figure 8**). There are 41 nodes in the HvDJ protein interaction network, each capable of interacting with the others. We also found several HvDJs exhibiting direct interactions, including HvDJA09 with HvDJC07, HvDJC22, HvDJC51 and HvDJA05, HvDJC25 with HvDJC30, HvDJA05 with HvDJC67, HvDJC74, HvDJC75, HvDJC77, HvDJC78, and HvDJB08, HvDJC73 with HvDJC77. Core genes function as central hubs that play pivotal roles in network modules. Among them, HvDJA09 and HvDJA05 played core and pivotal roles in the complex regulatory network. Additionally, other proteins such as HSP70-7, HSP70-15, HSP70-17, and DJC82 were also identified as targets in the core network of J-domain proteins.

## 4 Discussion

With the rapid development of gene sequencing technology, an increasing number of plant genomes have been published, facilitating the identification of the variable gene families. To date, 129 JDP homologs in *Arabidopsis* (Zhang et al., 2018), 115 in rice (Luo et al., 2019), 76 in pepper (Fan et al., 2017), 236 in wheat (Liu et al., 2022), 86 in citrus (Tian et al., 2024), and 91 in maize (Li et al., 2024) have been identified and characterized. In this study, 109 *HvDJ* genes were identified in the barley genome (**Table 1**), providing valuable genetic information for a deeper understanding of their functions. Barley *JDPs* exhibit a wide range of sequence lengths and significant differences in exon numbers (**Figure 1**). Moreover, there are dramatic differences in motif distribution and number among HvDJs (**Figure 1**). With respect to isoelectric point (pI), HvDJ proteins range from 4.22 (HvDJC61) to 11.18 (HvDJC66) (**Table 1**), with 65% exhibiting a pI >7, similar to those of TaDnaJs and CbuDnaJs (Liu et al., 2022; Yang et al., 2023).

Features in gene and protein structure can elucidate the characteristics of gene families and guide functional research (Rogozin et al., 2005). Here, gene structural analysis of *HvDJs* revealed considerable variation in the number and distribution of introns and exons (**Figure 1B**), suggesting functional divergence in their response to salt stress. It is well documented that *cis*-elements in gene promoters regulate gene expression during plant growth and development, as well as adaption to environmental stimuli (Guo et al., 2024; Li et al., 2018). In this study, *cis*-elements within the 2-kb upstream regions of *HvDJ* genes were analyzed using the PlantCARE program (Lescot et al., 2002). Four major groups of *cis*-elements were identified: hormone-responsive, stress-related, tissue-specific, and transcription factor-binding elements (**Figure 2**; **Supplementary Table S3**). These findings suggest that *HvDJs* may be involved in the responses to abiotic stress, hormone regulation, and transcriptional regulation.

Gene duplication serves not only as a primary source of evolutionary innovation but also as a major driving force for gene family expansion (Schmutz et al., 2010). In barley, 109 *JDP* genes were unevenly distributed across the seven chromosomes (**Figure 3A**). Thirty *HvDJ* genes have undergone gene duplication, including both tandem duplication and segmental duplication events (**Figure 3**). Three tandem and 21 segmental duplication events were observed. These results suggest that both tandem and segmental duplications have played vital roles in the expansion of the *HvDJ* gene family in barley. Collinearity analysis revealed that *HvDJ* genes are more closely related to monocotyledonous plants, particularly wheat (**Figure 4**). These findings highlight the evolutionary origins and genetic relationships of *JDPs* between barley and other plant species.

JDPS have been reported to be involved in responses to various biotic and abiotic stresses. Silencing of *NtMPIP1*, a DnaJ-like protein in tobacco, significantly inhibited infection by tobacco mosaic virus (TMV) (Shimizu et al., 2009). Overexpression of soybean HSP40 induced hypersensitive response (HR)-like cell death in tobacco leaves (Liu and Whitham, 2013). Cotton GhDNAJ1 positively regulates resistance to *V. dahlia* (Feng et al., 2021). In addition, *JDPs* have been reported to play important roles in regulating abiotic stress tolerance, including responses to heat, drought, chilling, and salt stress (Kong et al., 2014a; Lee et al., 2018; Liu et al., 2023; Wang et al., 2015; Yamamoto et al., 2020). To examine the expression profiles of *HvDJ* genes in response to salt stress, RNA-seq data were obtained from barley seedlings sampled at four time points of salt exposure (0 h, 1 h, 6 h, and 24 h) (Zhang et al., 2020). A total of 37 *HvDJ* genes were identified as salt-responsive, exhibiting distinct expression patterns across the time points. Eight genes (*HvDJC09*, *HvDJC11*, *HvDJB03*, *HvDJC33*, *HvDJB06*, *HvDJC46*, *HvDJC58*, and *HvDJC59*) exhibited differential expression at 1 h, 6 h, and 24 h after salt stress exposure. Among them, *HvDJC09*, *HvDJB03*, *HvDJC33*, and *HvDJC46* were upregulated, while *HvDJB06*, *HvDJC58*, and *HvDJC59* were downregulated (**Figure 5**). qRT-PCR further confirmed that *HvDJC09*, *HvDJB03*, *HvDJC33*, and *HvDJC46* were upregulated by salt stress, with peak expression observed after 48 h of exposure (**Figure 6**). Additionally, –12 genes (*HvDJB02*, *HvDJC29*, *HvDJB05*, *HvDJC40*, *HvDJC47*, *HvDJC66*, *HvDJC79*, *HvDJB08*, *HvDJC88*, *HvDJC89*, *HvDJC90*, and *HvDJC91*) were differentially expressed at 6 h and 24 h, with only *HvDJC40* showing downregulation at both 1 h and 6 h. Seventeen genes were differentially expressed at only one time point following salt stress (**Figure 5**). These findings suggest that *HvDJ* genes vary in their expression patterns and function in response to salt stress. Furthermore, we predicted the protein structures of seven differentially expressed genes (*HvDJC09*, *HvDJB03*, *HvDJC33*, *HvDJB06*, *HvDJC46*, *HvDJC58*, and *HvDJC59*) at three time points (1 h, 6 h. and 24 h) to explore their potential roles in salt stress response (**Figure 7**). Notably, several potential genes for salinity tolerance were identified on chromosome 4H, including *HvDJC53* (Fan et al., 2016). In addition, QTLs for grain yield relative to control conditions were found near the QTL for salinity tolerance score on chromosome 3H, where *HvDJC10*, *HvDJB04*, and *HvDJC28* are located (Liu et al., 2017). Additionally, 41 HvDJs were predicted to interact with one another, with the interaction of HvDJA09 and HvDJA05 serving as the central node in the complex regulatory network (**Figure 8**). Overall, the results of protein structure and interaction analysis provided new insight into the biological functions of HvDJs.

## 5 Conclusion

In this study, 109 *JDP* genes in barley were identified and characterized. Our results showed that tandem and segmental duplications are the driving forces behind *JDP* gene family expansion. A total of 37 *HvDJs* showed differential expression under salt stress, with *HvDJB06* and *HvDJC46* showing the highest expression levels. In total, 41 nodes were identified in the HvDJ protein interaction network, with HvDJA09 and HvDJA05 playing central roles.

## Data Availability

The original contributions presented in the study are included in the article/supplementary material, further inquiries can be directed to the corresponding author/s.
