# Supplementary material for: The potential functions of HvDJ genes in regulating salt tolerance in barley
Source: Front Plant Sci. 2025 Jul 10;16:1574097. doi: 10.3389/fpls.2025.1574097 (PMC12287002; doi:10.3389/fpls.2025.1574097)
Supplement: Supplementary file 1 [file SupplementaryFile1.docx]

| **Table S1. The conserved domain of HvDJs** | | | |
| --- | --- | --- | --- |
| **Gene name** | **From** | **To** | **domain** |
| HvDJA01 | 71 | 139 | DnaJ |
| HvDJA01 | 193 | 411 | DnaJ_C |
| HvDJA01 | 220 | 284 | DnaJ_CXXCXGXG |
| HvDJA02 | 69 | 142 | DnaJ |
| HvDJA02 | 183 | 396 | DnaJ_C |
| HvDJA02 | 210 | 269 | DnaJ_CXXCXGXG |
| HvDJA03 | 179 | 240 | DnaJ |
| HvDJA03 | 295 | 510 | DnaJ_C |
| HvDJA03 | 320 | 383 | DnaJ_CXXCXGXG |
| HvDJA04 | 14 | 80 | DnaJ |
| HvDJA04 | 121 | 348 | DnaJ_C |
| HvDJA04 | 150 | 216 | DnaJ_CXXCXGXG |
| HvDJA05 | 15 | 81 | DnaJ |
| HvDJA05 | 123 | 345 | DnaJ_C |
| HvDJA05 | 150 | 216 | DnaJ_CXXCXGXG |
| HvDJA06 | 15 | 81 | DnaJ |
| HvDJA06 | 125 | 347 | DnaJ_C |
| HvDJA06 | 152 | 218 | DnaJ_CXXCXGXG |
| HvDJA07 | 71 | 137 | DnaJ |
| HvDJA07 | 194 | 412 | DnaJ_C |
| HvDJA07 | 221 | 285 | DnaJ_CXXCXGXG |
| HvDJA08 | 75 | 149 | DnaJ |
| HvDJA08 | 197 | 410 | DnaJ_C |
| HvDJA08 | 224 | 284 | DnaJ_CXXCXGXG |
| HvDJA09 | 73 | 137 | DnaJ |
| HvDJA09 | 206 | 416 | DnaJ_C |
| HvDJA09 | 233 | 289 | DnaJ_CXXCXGXG |
| HvDJB01 | 4 | 70 | DnaJ |
| HvDJB01 | 170 | 331 | DnaJ_C |
| HvDJB02 | 8 | 104 | DnaJ |
| HvDJB02 | 148 | 313 | DnaJ_C |
| HvDJB03 | 4 | 74 | DnaJ |
| HvDJB03 | 192 | 355 | DnaJ_C |
| HvDJB04 | 4 | 74 | DnaJ |
| HvDJB04 | 169 | 332 | DnaJ_C |
| HvDJB05 | 8 | 69 | DnaJ |
| HvDJB05 | 144 | 308 | DnaJ_C |
| HvDJB06 | 5 | 84 | DnaJ |
| HvDJB06 | 131 | 300 | DnaJ_C |
| HvDJB07 | 2 | 107 | DnaJ |
| HvDJB07 | 160 | 323 | DnaJ_C |
| HvDJB08 | 4 | 75 | DnaJ |
| HvDJB08 | 163 | 326 | DnaJ_C |
| HvDJC01 | 174 | 226 | DnaJ |
| HvDJC02 | 66 | 127 | DnaJ |
| HvDJC03 | 29 | 91 | DnaJ |
| HvDJC04 | 12 | 77 | DnaJ |
| HvDJC05 | 1532 | 1573 | DnaJ |
| HvDJC06 | 1330 | 1412 | DnaJ |
| HvDJC07 | 106 | 167 | DnaJ |
| HvDJC08 | 12 | 79 | DnaJ |
| HvDJC09 | 10 | 74 | DnaJ |
| HvDJC10 | 7 | 68 | DnaJ |
| HvDJC11 | 81 | 143 | DnaJ |
| HvDJC12 | 1452 | 1497 | DnaJ |
| HvDJC13 | 98 | 177 | DnaJ |
| HvDJC14 | 5 | 66 | DnaJ |
| HvDJC15 | 72 | 140 | DnaJ |
| HvDJC16 | 72 | 124 | DnaJ |
| HvDJC16 | 72 | 124 | DnaJ |
| HvDJC17 | 33 | 83 | DnaJ |
| HvDJC18 | 172 | 229 | DnaJ |
| HvDJC19 | 99 | 161 | DnaJ |
| HvDJC20 | 68 | 129 | DnaJ |
| HvDJC21 | 321 | 377 | DnaJ |
| HvDJC22 | 53 | 121 | DnaJ |
| HvDJC23 | 213 | 269 | DnaJ |
| HvDJC24 | 30 | 91 | DnaJ |
| HvDJC25 | 62 | 127 | DnaJ |
| HvDJC26 | 57 | 101 | DnaJ |
| HvDJC27 | 1025 | 1098 | DnaJ |
| HvDJC28 | 64 | 132 | DnaJ |
| HvDJC29 | 5 | 72 | DnaJ |
| HvDJC30 | 55 | 117 | DnaJ |
| HvDJC31 | 134 | 195 | DnaJ |
| HvDJC32 | 66 | 127 | DnaJ |
| HvDJC33 | 10 | 79 | DnaJ |
| HvDJC34 | 1550 | 1607 | DnaJ |
| HvDJC35 | 7 | 68 | DnaJ |
| HvDJC36 | 139 | 201 | DnaJ |
| HvDJC37 | 65 | 126 | DnaJ |
| HvDJC38 | 64 | 128 | DnaJ |
| HvDJC39 | 66 | 127 | DnaJ |
| HvDJC40 | 352 | 415 | DnaJ |
| HvDJC41 | 172 | 233 | DnaJ |
| HvDJC42 | 98 | 161 | DnaJ |
| HvDJC43 | 66 | 127 | DnaJ |
| HvDJC44 | 865 | 930 | DnaJ |
| HvDJC45 | 81 | 138 | DnaJ |
| HvDJC46 | 38 | 95 | DnaJ |
| HvDJC47 | 417 | 449 | DnaJ |
| HvDJC48 | 21 | 87 | DnaJ |
| HvDJC49 | 28 | 89 | DnaJ |
| HvDJC50 | 38 | 95 | DnaJ |
| HvDJC51 | 76 | 135 | DnaJ |
| HvDJC52 | 589 | 630 | DnaJ |
| HvDJC53 | 66 | 127 | DnaJ |
| HvDJC54 | 288 | 352 | DnaJ |
| HvDJC55 | 846 | 904 | DnaJ |
| HvDJC56 | 9 | 74 | DnaJ |
| HvDJC57 | 106 | 167 | DnaJ |
| HvDJC58 | 40 | 106 | DnaJ |
| HvDJC59 | 54 | 120 | DnaJ |
| HvDJC60 | 78 | 120 | DnaJ |
| HvDJC61 | 12 | 80 | DnaJ |
| HvDJC62 | 72 | 132 | DnaJ |
| HvDJC63 | 6 | 68 | DnaJ |
| HvDJC64 | 529 | 580 | DnaJ |
| HvDJC65 | 44 | 104 | DnaJ |
| HvDJC66 | 57 | 101 | DnaJ |
| HvDJC67 | 73 | 126 | DnaJ |
| HvDJC68 | 67 | 121 | DnaJ |
| HvDJC69 | 63 | 120 | DnaJ |
| HvDJC70 | 439 | 494 | DnaJ |
| HvDJC71 | 66 | 120 | DnaJ |
| HvDJC72 | 69 | 134 | DnaJ |
| HvDJC73 | 31 | 93 | DnaJ |
| HvDJC74 | 66 | 128 | DnaJ |
| HvDJC75 | 12 | 74 | DnaJ |
| HvDJC76 | 27 | 89 | DnaJ |
| HvDJC77 | 33 | 95 | DnaJ |
| HvDJC78 | 16 | 84 | DnaJ |
| HvDJC79 | 15 | 83 | DnaJ |
| HvDJC80 | 28 | 90 | DnaJ |
| HvDJC81 | 100 | 168 | DnaJ |
| HvDJC82 | 21 | 85 | DnaJ |
| HvDJC83 | 52 | 110 | DnaJ |
| HvDJC84 | 67 | 128 | DnaJ |
| HvDJC85 | 7 | 68 | DnaJ |
| HvDJC86 | 49 | 108 | DnaJ |
| HvDJC87 | 6 | 68 | DnaJ |
| HvDJC88 | 8 | 69 | DnaJ |
| HvDJC89 | 24 | 73 | DnaJ |
| HvDJC90 | 55 | 114 | DnaJ |
| HvDJC91 | 55 | 114 | DnaJ |
| HvDJC92 | 46 | 106 | DnaJ |

| **Table S2. Analysis and distribution of conserved motifs in HvDJ proteins** | | |
| --- | --- | --- |
| **Motif** | **Width** | **Best possible match** |
| 1 | 80 | GVRVQEAITEPFTVKDHRGELYKKHYPPALKDEVWRLEKIGKDGAFHKRLNESGIYTVEDFLRLLVKDPQKLRKJLGMGM |
| 2 | 49 | SGPLSSAKVEILVLEGDFNNEDEEEWTEEEFNSHIVKEREGKRPLLTGD |
| 3 | 40 | VVVTLKDGVATIGDLAFTDNSSWIRSRKFRLGARVASGFY |
| 4 | 41 | PNLQLQFLDRLSLPJFTGGKVEGENGAAIKVVLLDTNNGVV |
| 5 | 29 | WESLIEHAKTCVLGGKJYIYYVEEHNVEL |
| 6 | 80 | AIFNDJYEFCGLIAGDQFYSSENLDDGQKLFADGLVKKAYDDWMYVIEYDGKALLNPKPKKKAVLTRQPEARAPAAYVQR |
| 7 | 56 | PYMPSPLPTFGFEGDPSRPSGKAVVGWLKIKAAMRWGIFVRKKAAERRAQJVELDD |
| 8 | 56 | PKRPRPPALASVIVEALKADSJQKLCSSLEPIJRRVVSEEIZRALAKHGPAAIRSR |
| 9 | 60 | GQEDFTKEEFNKQIYJYKGKESVLTTVNLVNGEAYIGSFFFTESSQRKRLRLTARVKKQD |
| 10 | 30 | SLNDLDQAQKAKVKQLKKSAYEQFENLEES |

| **Table S3. Details of the cis-elements identified in *JDP* gene family** | | | |
| --- | --- | --- | --- |
| **Gene** | **start position** | **stop position** | **putative cis-elements** |
| *HvDJC01* | 1284 | 1279 | Abscisic acid responsive element |
| *HvDJC01* | 1650 | 1656 | Anaerobic responsive element |
| *HvDJC01* | 1706 | 1712 | Anaerobic responsive element |
| *HvDJC01* | 107 | 100 | Gibberellin-responsive element |
| *HvDJC01* | 48 | 54 | Light responsive element |
| *HvDJC01* | 1068 | 1062 | Light responsive element |
| *HvDJC01* | 629 | 620 | Light responsive element |
| *HvDJC01* | 1126 | 1132 | Light responsive element |
| *HvDJC01* | 1284 | 1290 | Light responsive element |
| *HvDJC01* | 1632 | 1638 | Light responsive element |
| *HvDJC01* | 350 | 357 | Light responsive element |
| *HvDJC01* | 535 | 542 | Light responsive element |
| *HvDJC01* | 550 | 555 | MeJA-responsive element |
| *HvDJC01* | 717 | 712 | MeJA-responsive element |
| *HvDJC01* | 107 | 113 | MYB |
| *HvDJC01* | 257 | 251 | MYB |
| *HvDJC01* | 1318 | 1312 | MYB |
| *HvDJC01* | 107 | 113 | MYB |
| *HvDJC01* | 257 | 251 | MYB |
| *HvDJC01* | 234 | 228 | MYC |
| *HvDJC01* | 374 | 380 | MYC |
| *HvDJC01* | 389 | 395 | MYC |
| *HvDJC01* | 617 | 611 | MYC |
| *HvDJC01* | 862 | 856 | MYC |
| *HvDJC01* | 917 | 911 | MYC |
| *HvDJC01* | 1036 | 1042 | MYC |
| *HvDJC01* | 1177 | 1183 | MYC |
| *HvDJC01* | 1348 | 1342 | MYC |
| *HvDJC01* | 1548 | 1554 | MYC |
| *HvDJC01* | 1808 | 1799 | Salicylic acid responsive element |
| *HvDJC01* | 325 | 316 | Zein metabolism regulation |
| *HvDJC01* | 1283 | 1274 | Zein metabolism regulation |
| *HvDJC02* | 269 | 263 | Anaerobic responsive element |
| *HvDJC02* | 1070 | 1064 | Anaerobic responsive element |
| *HvDJC02* | 1020 | 1029 | Defence responsive element |
| *HvDJC02* | 1903 | 1894 | Defence responsive element |
| *HvDJC02* | 21 | 15 | Drought responsive element |
| *HvDJC02* | 829 | 823 | Drought responsive element |
| *HvDJC02* | 472 | 479 | Endosperm |
| *HvDJC02* | 1417 | 1410 | Endosperm |
| *HvDJC02* | 382 | 389 | Gibberellin-responsive element |
| *HvDJC02* | 111 | 103 | Light responsive element |
| *HvDJC02* | 155 | 147 | Light responsive element |
| *HvDJC02* | 963 | 955 | Light responsive element |
| *HvDJC02* | 1223 | 1215 | Light responsive element |
| *HvDJC02* | 1845 | 1853 | Light responsive element |
| *HvDJC02* | 1456 | 1450 | Light responsive element |
| *HvDJC02* | 1518 | 1512 | Light responsive element |
| *HvDJC02* | 1559 | 1553 | Light responsive element |
| *HvDJC02* | 1901 | 1910 | Light responsive element |
| *HvDJC02* | 1302 | 1309 | Light responsive element |
| *HvDJC02* | 619 | 609 | Light responsive element |
| *HvDJC02* | 124 | 118 | Light responsive element |
| *HvDJC02* | 1507 | 1502 | MeJA-responsive element |
| *HvDJC02* | 21 | 15 | MYB |
| *HvDJC02* | 829 | 823 | MYB |
| *HvDJC02* | 1408 | 1414 | MYB |
| *HvDJC02* | 613 | 619 | MYB |
| *HvDJC02* | 1468 | 1462 | MYB |
| *HvDJC02* | 1468 | 1462 | MYB |
| *HvDJC02* | 960 | 966 | MYC |
| *HvDJC02* | 1241 | 1235 | MYC |
| *HvDJC02* | 1392 | 1398 | MYC |
| *HvDJC02* | 1587 | 1581 | MYC |
| *HvDJC02* | 1613 | 1619 | MYC |
| *HvDJC02* | 1656 | 1650 | MYC |
| *HvDJC02* | 552 | 561 | Salicylic acid responsive element |
| *HvDJC02* | 1197 | 1188 | Salicylic acid responsive element |
| *HvDJC02* | 800 | 808 | Seed-specific regulation |
| *HvDJC02* | 1655 | 1646 | Zein metabolism regulation |
| *HvDJB01* | 388 | 383 | Abscisic acid responsive element |
| *HvDJB01* | 794 | 800 | Anaerobic responsive element |
| *HvDJB01* | 1875 | 1881 | Anaerobic responsive element |
| *HvDJB01* | 1699 | 1705 | Auxin-responsive element |
| *HvDJB01* | 731 | 722 | Circadian |
| *HvDJB01* | 782 | 773 | Circadian |
| *HvDJB01* | 1072 | 1061 | Drought responsive element |
| *HvDJB01* | 476 | 469 | Gibberellin-responsive element |
| *HvDJB01* | 1241 | 1234 | Gibberellin-responsive element |
| *HvDJB01* | 1818 | 1811 | Gibberellin-responsive element |
| *HvDJB01* | 1223 | 1216 | Gibberellin-responsive element |
| *HvDJB01* | 1754 | 1746 | Light responsive element |
| *HvDJB01* | 1151 | 1145 | Light responsive element |
| *HvDJB01* | 1645 | 1639 | Light responsive element |
| *HvDJB01* | 1529 | 1536 | Light responsive element |
| *HvDJB01* | 388 | 394 | Light responsive element |
| *HvDJB01* | 873 | 867 | Light responsive element |
| *HvDJB01* | 613 | 619 | Low-temperature responsive element |
| *HvDJB01* | 358 | 353 | MeJA-responsive element |
| *HvDJB01* | 1694 | 1699 | MeJA-responsive element |
| *HvDJB01* | 1072 | 1078 | MYB |
| *HvDJB01* | 1241 | 1247 | MYB |
| *HvDJB01* | 1541 | 1547 | MYB |
| *HvDJB01* | 1818 | 1824 | MYB |
| *HvDJB01* | 1954 | 1960 | MYB |
| *HvDJB01* | 1241 | 1247 | MYB |
| *HvDJB01* | 1541 | 1547 | MYB |
| *HvDJB01* | 1818 | 1824 | MYB |
| *HvDJB01* | 992 | 999 | MYC |
| *HvDJB01* | 971 | 965 | MYC |
| *HvDJC03* | 192 | 187 | Abscisic acid responsive element |
| *HvDJC03* | 1714 | 1708 | Abscisic acid responsive element |
| *HvDJC03* | 1715 | 1720 | Abscisic acid responsive element |
| *HvDJC03* | 1783 | 1778 | Abscisic acid responsive element |
| *HvDJC03* | 1713 | 1705 | Abscisic acid responsive element |
| *HvDJC03* | 1703 | 1697 | Auxin-responsive element |
| *HvDJC03* | 237 | 246 | Circadian |
| *HvDJC03* | 616 | 625 | Circadian |
| *HvDJC03* | 844 | 853 | Circadian |
| *HvDJC03* | 717 | 710 | Gibberellin-responsive element |
| *HvDJC03* | 1003 | 995 | Light responsive element |
| *HvDJC03* | 415 | 421 | Light responsive element |
| *HvDJC03* | 658 | 664 | Light responsive element |
| *HvDJC03* | 192 | 198 | Light responsive element |
| *HvDJC03* | 1712 | 1703 | Light responsive element |
| *HvDJC03* | 1714 | 1708 | Light responsive element |
| *HvDJC03* | 1783 | 1789 | Light responsive element |
| *HvDJC03* | 1714 | 1708 | Light responsive element |
| *HvDJC03* | 990 | 996 | Light responsive element |
| *HvDJC03* | 1838 | 1832 | Light responsive element |
| *HvDJC03* | 1684 | 1690 | Light responsive element |
| *HvDJC03* | 641 | 648 | Light responsive element |
| *HvDJC03* | 862 | 856 | Low-temperature responsive element |
| *HvDJC03* | 1792 | 1798 | Low-temperature responsive element |
| *HvDJC03* | 1670 | 1675 | MeJA-responsive element |
| *HvDJC03* | 321 | 327 | MYB |
| *HvDJC03* | 1680 | 1674 | MYB |
| *HvDJC03* | 62 | 68 | MYC |
| *HvDJC03* | 574 | 568 | MYC |
| *HvDJC03* | 1146 | 1152 | MYC |
| *HvDJC03* | 1217 | 1211 | MYC |
| *HvDJC03* | 1315 | 1309 | MYC |
| *HvDJC03* | 1729 | 1735 | MYC |
| *HvDJC03* | 1767 | 1758 | Zein metabolism regulation |
| *HvDJC04* | 166 | 161 | Abscisic acid responsive element |
| *HvDJC04* | 378 | 383 | Abscisic acid responsive element |
| *HvDJC04* | 523 | 529 | Abscisic acid responsive element |
| *HvDJC04* | 524 | 529 | Abscisic acid responsive element |
| *HvDJC04* | 1704 | 1697 | Abscisic acid responsive element |
| *HvDJC04* | 560 | 566 | Anaerobic responsive element |
| *HvDJC04* | 967 | 961 | Anaerobic responsive element |
| *HvDJC04* | 1005 | 999 | Anaerobic responsive element |
| *HvDJC04* | 1797 | 1788 | Defence responsive element |
| *HvDJC04* | 763 | 770 | Gibberellin-responsive element |
| *HvDJC04* | 495 | 503 | Light responsive element |
| *HvDJC04* | 995 | 987 | Light responsive element |
| *HvDJC04* | 786 | 792 | Light responsive element |
| *HvDJC04* | 1145 | 1139 | Light responsive element |
| *HvDJC04* | 1882 | 1876 | Light responsive element |
| *HvDJC04* | 166 | 172 | Light responsive element |
| *HvDJC04* | 314 | 320 | Light responsive element |
| *HvDJC04* | 377 | 371 | Light responsive element |
| *HvDJC04* | 523 | 529 | Light responsive element |
| *HvDJC04* | 523 | 529 | Light responsive element |
| *HvDJC04* | 1453 | 1459 | Light responsive element |
| *HvDJC04* | 924 | 930 | Light responsive element |
| *HvDJC04* | 1717 | 1723 | Light responsive element |
| *HvDJC04* | 290 | 284 | Low-temperature responsive element |
| *HvDJC04* | 1940 | 1946 | Low-temperature responsive element |
| *HvDJC04* | 342 | 337 | MeJA-responsive element |
| *HvDJC04* | 1697 | 1692 | MeJA-responsive element |
| *HvDJC04* | 1747 | 1742 | MeJA-responsive element |
| *HvDJC04* | 1673 | 1666 | MYB |
| *HvDJC04* | 332 | 338 | MYB |
| *HvDJC04* | 734 | 728 | MYB |
| *HvDJC04* | 1452 | 1446 | MYB |
| *HvDJC04* | 68 | 62 | MYB |
| *HvDJC04* | 392 | 386 | MYB |
| *HvDJC04* | 332 | 338 | MYB |
| *HvDJC04* | 1452 | 1446 | MYB |
| *HvDJC04* | 115 | 109 | MYC |
| *HvDJC04* | 307 | 313 | MYC |
| *HvDJC05* | 430 | 425 | Abscisic acid responsive element |
| *HvDJC05* | 755 | 760 | Abscisic acid responsive element |
| *HvDJC05* | 866 | 861 | Abscisic acid responsive element |
| *HvDJC05* | 866 | 860 | Abscisic acid responsive element |
| *HvDJC05* | 866 | 872 | Abscisic acid responsive element |
| *HvDJC05* | 1449 | 1455 | Anaerobic responsive element |
| *HvDJC05* | 923 | 916 | Auxin-responsive element |
| *HvDJC05* | 333 | 324 | Light responsive element |
| *HvDJC05* | 646 | 655.5 | Light responsive element |
| *HvDJC05* | 430 | 436 | Light responsive element |
| *HvDJC05* | 754 | 748 | Light responsive element |
| *HvDJC05* | 863 | 872 | Light responsive element |
| *HvDJC05* | 866 | 860 | Light responsive element |
| *HvDJC05* | 459 | 453 | Light responsive element |
| *HvDJC05* | 548 | 554 | Light responsive element |
| *HvDJC05* | 19 | 11 | Light responsive element |
| *HvDJC05* | 1608 | 1614 | Light responsive element |
| *HvDJC05* | 1070 | 1064 | Light responsive element |
| *HvDJC05* | 737 | 743 | Low-temperature responsive element |
| *HvDJC05* | 432 | 427 | MeJA-responsive element |
| *HvDJC05* | 1166 | 1173 | MYB |
| *HvDJC05* | 1761 | 1755 | MYB |
| *HvDJC05* | 113 | 119 | MYC |
| *HvDJC05* | 337 | 343 | MYC |
| *HvDJC05* | 646 | 640 | MYC |
| *HvDJC05* | 1199 | 1193 | MYC |
| *HvDJC05* | 1443 | 1449 | MYC |
| *HvDJC06* | 974 | 983 | Abscisic acid responsive element |
| *HvDJC06* | 976 | 971 | Abscisic acid responsive element |
| *HvDJC06* | 1089 | 1094 | Abscisic acid responsive element |
| *HvDJC06* | 1458 | 1449 | Abscisic acid responsive element |
| *HvDJC06* | 1768 | 1774 | Anaerobic responsive element |
| *HvDJC06* | 566 | 572 | Anoxic responsive element |
| *HvDJC06* | 1386 | 1393 | Auxin-responsive element |
| *HvDJC06* | 1412 | 1405 | Auxin-responsive element |
| *HvDJC06* | 509 | 515 | Auxin-responsive element |
| *HvDJC06* | 757 | 751 | Drought responsive element |
| *HvDJC06* | 1113 | 1123 | Light responsive element |
| *HvDJC06* | 976 | 982 | Light responsive element |
| *HvDJC06* | 1448 | 1454 | Light responsive element |
| *HvDJC06* | 1088 | 1082 | Light responsive element |
| *HvDJC06* | 1780 | 1789 | Light responsive element |
| *HvDJC06* | 563 | 557 | Light responsive element |
| *HvDJC06* | 1313 | 1307 | Light responsive element |
| *HvDJC06* | 1920 | 1914 | Light responsive element |
| *HvDJC06* | 1930 | 1924 | Light responsive element |
| *HvDJC06* | 1957 | 1963 | Light responsive element |
| *HvDJC06* | 1035 | 1042 | Light responsive element |
| *HvDJC06* | 658 | 653 | MeJA-responsive element |
| *HvDJC06* | 1100 | 1095 | MeJA-responsive element |
| *HvDJC06* | 1046 | 1039 | MYB |
| *HvDJC06* | 757 | 751 | MYB |
| *HvDJC06* | 806 | 800 | MYB |
| *HvDJC06* | 119 | 113 | MYC |
| *HvDJC06* | 1578 | 1572 | MYC |
| *HvDJC06* | 1616 | 1610 | MYC |
| *HvDJC07* | 246 | 251 | Abscisic acid responsive element |
| *HvDJC07* | 362 | 355 | Abscisic acid responsive element |
| *HvDJC07* | 1171 | 1176 | Abscisic acid responsive element |
| *HvDJC07* | 1170 | 1176 | Abscisic acid responsive element |
| *HvDJC07* | 1170 | 1164 | Abscisic acid responsive element |
| *HvDJC07* | 1754 | 1748 | Anaerobic responsive element |
| *HvDJC07* | 650 | 661 | Light responsive element |
| *HvDJC07* | 1787 | 1778 | Light responsive element |
| *HvDJC07* | 245 | 239 | Light responsive element |
| *HvDJC07* | 1170 | 1176 | Light responsive element |
| *HvDJC07* | 1697 | 1703 | Light responsive element |
| *HvDJC07* | 1479 | 1485 | Light responsive element |
| *HvDJC07* | 1529 | 1535 | Light responsive element |
| *HvDJC07* | 761 | 752 | Light responsive element |
| *HvDJC07* | 1176 | 1169 | Light responsive element |
| *HvDJC07* | 509 | 514 | MeJA-responsive element |
| *HvDJC07* | 1111 | 1106 | MeJA-responsive element |
| *HvDJC07* | 1437 | 1442 | MeJA-responsive element |
| *HvDJC07* | 80 | 74 | MYB |
| *HvDJC07* | 1335 | 1341 | MYB |
| *HvDJC07* | 298 | 304 | MYB |
| *HvDJC07* | 1549 | 1555 | MYB |
| *HvDJC07* | 949 | 942 | MYC |
| *HvDJC07* | 1289 | 1282 | MYC |
| *HvDJC07* | 588 | 594 | MYC |
| *HvDJC07* | 135 | 126 | Zein metabolism regulation |
| *HvDJC08* | 343 | 348 | Abscisic acid responsive element |
| *HvDJC08* | 1146 | 1141 | Abscisic acid responsive element |
| *HvDJC08* | 1146 | 1140 | Abscisic acid responsive element |
| *HvDJC08* | 1146 | 1152 | Abscisic acid responsive element |
| *HvDJC08* | 974 | 968 | Anaerobic responsive element |
| *HvDJC08* | 1169 | 1175 | Anaerobic responsive element |
| *HvDJC08* | 1630 | 1636 | Anaerobic responsive element |
| *HvDJC08* | 743 | 749 | Anoxic responsive element |
| *HvDJC08* | 1810 | 1816 | Anoxic responsive element |
| *HvDJC08* | 1872 | 1878 | Anoxic responsive element |
| *HvDJC08* | 1902 | 1908 | Anoxic responsive element |
| *HvDJC08* | 1196 | 1189 | Endosperm |
| *HvDJC08* | 90 | 97 | Gibberellin-responsive element |
| *HvDJC08* | 892 | 885 | Gibberellin-responsive element |
| *HvDJC08* | 1339 | 1349 | Light responsive element |
| *HvDJC08* | 998 | 1005 | Light responsive element |
| *HvDJC08* | 1335 | 1345 | Light responsive element |
| *HvDJC08* | 1543 | 1550 | Light responsive element |
| *HvDJC08* | 1146 | 1140 | Light responsive element |
| *HvDJC08* | 342 | 336 | Light responsive element |
| *HvDJC08* | 1438 | 1448 | Light responsive element |
| *HvDJC08* | 1899 | 1893 | Light responsive element |
| *HvDJC08* | 786 | 791 | MeJA-responsive element |
| *HvDJC08* | 1773 | 1768 | MeJA-responsive element |
| *HvDJC08* | 1790 | 1795 | MeJA-responsive element |
| *HvDJC08* | 992 | 998 | MYB |
| *HvDJC08* | 117 | 111 | MYB |
| *HvDJC08* | 117 | 111 | MYB |
| *HvDJC08* | 476 | 482 | MYC |
| *HvDJC08* | 504 | 510 | MYC |
| *HvDJC08* | 816 | 822 | MYC |
| *HvDJC08* | 1317 | 1326 | Zein metabolism regulation |
| *HvDJB02* | 105 | 100 | Abscisic acid responsive element |
| *HvDJB02* | 907 | 912 | Abscisic acid responsive element |
| *HvDJB02* | 1395 | 1402 | Abscisic acid responsive element |
| *HvDJB02* | 1513 | 1518 | Abscisic acid responsive element |
| *HvDJB02* | 1959 | 1954 | Abscisic acid responsive element |
| *HvDJB02* | 85 | 91 | Anaerobic responsive element |
| *HvDJB02* | 513 | 519 | Anoxic responsive element |
| *HvDJB02* | 1919 | 1925 | Anoxic responsive element |
| *HvDJB02* | 506 | 500 | Auxin-responsive element |
| *HvDJB02* | 780 | 786 | Auxin-responsive element |
| *HvDJB02* | 1259 | 1265 | Auxin-responsive element |
| *HvDJB02* | 1440 | 1446 | Drought responsive element |
| *HvDJB02* | 839 | 832 | Gibberellin-responsive element |
| *HvDJB02* | 1188 | 1196 | Light responsive element |
| *HvDJB02* | 105 | 111 | Light responsive element |
| *HvDJB02* | 1512 | 1506 | Light responsive element |
| *HvDJB02* | 529 | 535 | Light responsive element |
| *HvDJB02* | 786 | 792 | Light responsive element |
| *HvDJB02* | 906 | 900 | Light responsive element |
| *HvDJB02* | 927 | 933 | Light responsive element |
| *HvDJB02* | 1959 | 1965 | Light responsive element |
| *HvDJB02* | 243 | 234 | Light responsive element |
| *HvDJB02* | 290 | 297 | Light responsive element |
| *HvDJB02* | 92 | 87 | MeJA-responsive element |
| *HvDJB02* | 905 | 910 | MeJA-responsive element |
| *HvDJB02* | 1603 | 1598 | MeJA-responsive element |
| *HvDJB02* | 1804 | 1799 | MeJA-responsive element |
| *HvDJB02* | 74 | 80 | MYB |
| *HvDJB02* | 463 | 469 | MYB |
| *HvDJB02* | 839 | 845 | MYB |
| *HvDJB02* | 876 | 882 | MYB |
| *HvDJB02* | 1056 | 1062 | MYB |
| *HvDJB02* | 1231 | 1225 | MYB |
| *HvDJB02* | 1443 | 1437 | MYB |
| *HvDJB02* | 1564 | 1570 | MYB |
| *HvDJB02* | 1794 | 1800 | MYB |
| *HvDJB02* | 569 | 575 | MYB |
| *HvDJB02* | 1440 | 1446 | MYB |
| *HvDJB02* | 920 | 914 | MYB |
| *HvDJB02* | 976 | 982 | MYB |
| *HvDJB02* | 1099 | 1093 | MYB |
| *HvDJB02* | 839 | 845 | MYB |
| *HvDJB02* | 1231 | 1225 | MYB |
| *HvDJB02* | 1443 | 1437 | MYB |
| *HvDJB02* | 1564 | 1570 | MYB |
| *HvDJB02* | 710 | 704 | MYC |
| *HvDJB02* | 1728 | 1734 | MYC |
| *HvDJB02* | 903 | 912 | Zein metabolism regulation |
| *HvDJC09* | 442 | 437 | Abscisic acid responsive element |
| *HvDJC09* | 629 | 635 | Abscisic acid responsive element |
| *HvDJC09* | 630 | 635 | Abscisic acid responsive element |
| *HvDJC09* | 1863 | 1869 | Anoxic responsive element |
| *HvDJC09* | 1871 | 1877 | Anoxic responsive element |
| *HvDJC09* | 465 | 459 | Auxin-responsive element |
| *HvDJC09* | 1029 | 1035 | Auxin-responsive element |
| *HvDJC09* | 715 | 723.5 | Cell cycle regulation |
| *HvDJC09* | 2011 | 2005 | Drought responsive element |
| *HvDJC09* | 156 | 149 | Gibberellin-responsive element |
| *HvDJC09* | 412 | 419 | Gibberellin-responsive element |
| *HvDJC09* | 1366 | 1357 | Light responsive element |
| *HvDJC09* | 819 | 825 | Light responsive element |
| *HvDJC09* | 1144 | 1151 | Light responsive element |
| *HvDJC09* | 629 | 635 | Light responsive element |
| *HvDJC09* | 418 | 412 | Light responsive element |
| *HvDJC09* | 442 | 448 | Light responsive element |
| *HvDJC09* | 539 | 545 | Light responsive element |
| *HvDJC09* | 629 | 635 | Light responsive element |
| *HvDJC09* | 1227 | 1220 | Light responsive element |
| *HvDJC09* | 1228 | 1222 | Light responsive element |
| *HvDJC09* | 1681 | 1671 | Light responsive element |
| *HvDJC09* | 1746 | 1752 | Low-temperature responsive element |
| *HvDJC09* | 153 | 148 | MeJA-responsive element |
| *HvDJC09* | 444 | 439 | MeJA-responsive element |
| *HvDJC09* | 700 | 695 | MeJA-responsive element |
| *HvDJC09* | 1592 | 1597 | MeJA-responsive element |
| *HvDJC09* | 26 | 20 | MYB |
| *HvDJC09* | 156 | 162 | MYB |
| *HvDJC09* | 236 | 230 | MYB |
| *HvDJC09* | 413 | 407 | MYB |
| *HvDJC09* | 713 | 719 | MYB |
| *HvDJC09* | 1997 | 1991 | MYB |
| *HvDJC09* | 2001 | 1995 | MYB |
| *HvDJC09* | 2005 | 1999 | MYB |
| *HvDJC09* | 135 | 129 | MYB |
| *HvDJC09* | 2011 | 2005 | MYB |
| *HvDJC09* | 175 | 181 | MYB |
| *HvDJC09* | 717 | 711 | MYB |
| *HvDJC09* | 1156 | 1150 | MYB |
| *HvDJC09* | 26 | 20 | MYB |
| *HvDJC09* | 156 | 162 | MYB |
| *HvDJC09* | 413 | 407 | MYB |
| *HvDJC09* | 374 | 380 | MYC |
| *HvDJC09* | 502 | 496 | MYC |
| *HvDJC09* | 1356 | 1350 | MYC |
| *HvDJC09* | 1621 | 1627 | MYC |
| *HvDJC09* | 1683 | 1677 | MYC |
| *HvDJC09* | 1109 | 1100 | Salicylic acid responsive element |
| *HvDJC09* | 1358 | 1350 | Seed-specific regulation |
| *HvDJC09* | 415 | 424 | Zein metabolism regulation |
| *HvDJC09* | 441 | 432 | Zein metabolism regulation |
| *HvDJC10* | 1492 | 1501 | Abscisic acid responsive element |
| *HvDJC10* | 1692 | 1697 | Abscisic acid responsive element |
| *HvDJC10* | 1724 | 1719 | Abscisic acid responsive element |
| *HvDJC10* | 1380 | 1386 | Auxin-responsive element |
| *HvDJC10* | 1147 | 1140 | Gibberellin-responsive element |
| *HvDJC10* | 1401 | 1394 | Gibberellin-responsive element |
| *HvDJC10* | 1976 | 1983 | Gibberellin-responsive element |
| *HvDJC10* | 196 | 188 | Light responsive element |
| *HvDJC10* | 1216 | 1208 | Light responsive element |
| *HvDJC10* | 397 | 406.5 | Light responsive element |
| *HvDJC10* | 1724 | 1730 | Light responsive element |
| *HvDJC10* | 1691 | 1685 | Light responsive element |
| *HvDJC10* | 1445 | 1451 | Light responsive element |
| *HvDJC10* | 1146 | 1136 | Light responsive element |
| *HvDJC10* | 504 | 514 | Light responsive element |
| *HvDJC10* | 505 | 513 | Light responsive element |
| *HvDJC10* | 545 | 555 | Light responsive element |
| *HvDJC10* | 546 | 554 | Light responsive element |
| *HvDJC10* | 1991 | 1997 | Light responsive element |
| *HvDJC10* | 1220 | 1214 | Light responsive element |
| *HvDJC10* | 1906 | 1912 | Light responsive element |
| *HvDJC10* | 206 | 201 | MeJA-responsive element |
| *HvDJC10* | 1429 | 1424 | MeJA-responsive element |
| *HvDJC10* | 1504 | 1499 | MeJA-responsive element |
| *HvDJC10* | 1690 | 1695 | MeJA-responsive element |
| *HvDJC10* | 1704 | 1709 | MeJA-responsive element |
| *HvDJC10* | 29 | 23 | MYB |
| *HvDJC10* | 478 | 472 | MYB |
| *HvDJC10* | 741 | 735 | MYB |
| *HvDJC10* | 784 | 790 | MYB |
| *HvDJC10* | 977 | 971 | MYB |
| *HvDJC10* | 988 | 994 | MYB |
| *HvDJC10* | 1147 | 1153 | MYB |
| *HvDJC10* | 1401 | 1407 | MYB |
| *HvDJC10* | 1977 | 1971 | MYB |
| *HvDJC10* | 71 | 77 | MYB |
| *HvDJC10* | 1452 | 1446 | MYB |
| *HvDJC10* | 478 | 472 | MYB |
| *HvDJC10* | 1147 | 1153 | MYB |
| *HvDJC10* | 1401 | 1407 | MYB |
| *HvDJC10* | 1977 | 1971 | MYB |
| *HvDJC10* | 29 | 23 | MYB |
| *HvDJC10* | 741 | 735 | MYB |
| *HvDJC10* | 977 | 971 | MYB |
| *HvDJC10* | 83 | 77 | MYC |
| *HvDJC10* | 218 | 224 | MYC |
| *HvDJC10* | 322 | 328 | MYC |
| *HvDJC10* | 397 | 391 | MYC |
| *HvDJC10* | 410 | 416 | MYC |
| *HvDJC10* | 515 | 521 | MYC |
| *HvDJC10* | 556 | 562 | MYC |
| *HvDJC10* | 573 | 579 | MYC |
| *HvDJC10* | 1127 | 1121 | MYC |
| *HvDJC10* | 1904 | 1911 | MYC |
| *HvDJC10* | 665 | 674 | Salicylic acid responsive element |
| *HvDJC11* | 179 | 184 | Abscisic acid responsive element |
| *HvDJC11* | 202 | 197 | Abscisic acid responsive element |
| *HvDJC11* | 247 | 242 | Abscisic acid responsive element |
| *HvDJC11* | 358 | 363 | Abscisic acid responsive element |
| *HvDJC11* | 830 | 835 | Abscisic acid responsive element |
| *HvDJC11* | 1677 | 1682 | Abscisic acid responsive element |
| *HvDJC11* | 1710 | 1704 | Abscisic acid responsive element |
| *HvDJC11* | 1711 | 1716 | Abscisic acid responsive element |
| *HvDJC11* | 202 | 196 | Abscisic acid responsive element |
| *HvDJC11* | 202 | 208 | Abscisic acid responsive element |
| *HvDJC11* | 1381 | 1375 | Anaerobic responsive element |
| *HvDJC11* | 1428 | 1422 | Anaerobic responsive element |
| *HvDJC11* | 1048 | 1042 | Anoxic responsive element |
| *HvDJC11* | 274 | 280 | Auxin-responsive element |
| *HvDJC11* | 1189 | 1195 | Auxin-responsive element |
| *HvDJC11* | 1927 | 1918 | Defence responsive element |
| *HvDJC11* | 336 | 342 | Drought responsive element |
| *HvDJC11* | 1310 | 1317 | Gibberellin-responsive element |
| *HvDJC11* | 1816 | 1809 | Gibberellin-responsive element |
| *HvDJC11* | 1404 | 1398 | Light responsive element |
| *HvDJC11* | 936 | 928 | Light responsive element |
| *HvDJC11* | 508 | 517.5 | Light responsive element |
| *HvDJC11* | 178 | 172 | Light responsive element |
| *HvDJC11* | 1710 | 1704 | Light responsive element |
| *HvDJC11* | 202 | 196 | Light responsive element |
| *HvDJC11* | 247 | 253 | Light responsive element |
| *HvDJC11* | 357 | 351 | Light responsive element |
| *HvDJC11* | 829 | 823 | Light responsive element |
| *HvDJC11* | 1676 | 1670 | Light responsive element |
| *HvDJC11* | 1710 | 1704 | Light responsive element |
| *HvDJC11* | 1179 | 1188 | Light responsive element |
| *HvDJC11* | 812 | 818 | Light responsive element |
| *HvDJC11* | 356 | 361 | MeJA-responsive element |
| *HvDJC11* | 708 | 713 | MeJA-responsive element |
| *HvDJC11* | 1500 | 1495 | MeJA-responsive element |
| *HvDJC11* | 336 | 342 | MYB |
| *HvDJC11* | 747 | 741 | MYB |
| *HvDJC11* | 1583 | 1589 | MYB |
| *HvDJC11* | 1706 | 1712 | MYB |
| *HvDJC11* | 1283 | 1289 | MYB |
| *HvDJC11* | 1706 | 1712 | MYB |
| *HvDJC11* | 322 | 316 | MYC |
| *HvDJC11* | 508 | 502 | MYC |
| *HvDJC11* | 1455 | 1449 | MYC |
| *HvDJB03* | 1781 | 1775 | Abscisic acid responsive element |
| *HvDJB03* | 1782 | 1787 | Abscisic acid responsive element |
| *HvDJB03* | 797 | 791 | Anaerobic responsive element |
| *HvDJB03* | 1529 | 1523 | Anaerobic responsive element |
| *HvDJB03* | 762 | 769 | Auxin-responsive element |
| *HvDJB03* | 878 | 884 | Auxin-responsive element |
| *HvDJB03* | 1617 | 1623 | Auxin-responsive element |
| *HvDJB03* | 1710 | 1718 | Light responsive element |
| *HvDJB03* | 792 | 798 | Light responsive element |
| *HvDJB03* | 1524 | 1518 | Light responsive element |
| *HvDJB03* | 1781 | 1775 | Light responsive element |
| *HvDJB03* | 460 | 454 | Light responsive element |
| *HvDJB03* | 1781 | 1775 | Light responsive element |
| *HvDJB03* | 980 | 973 | Light responsive element |
| *HvDJB03* | 981 | 975 | Light responsive element |
| *HvDJB03* | 1881 | 1892 | Light responsive element |
| *HvDJB03* | 127 | 122 | MeJA-responsive element |
| *HvDJB03* | 1606 | 1601 | MeJA-responsive element |
| *HvDJB03* | 1990 | 1995 | MeJA-responsive element |
| *HvDJB03* | 982 | 988 | MYB |
| *HvDJB03* | 1053 | 1059 | MYB |
| *HvDJB03* | 574 | 568 | MYB |
| *HvDJB03* | 1053 | 1059 | MYB |
| *HvDJB03* | 982 | 988 | MYB |
| *HvDJB03* | 802 | 795 | MYC |
| *HvDJB03* | 1534 | 1527 | MYC |
| *HvDJB03* | 833 | 841 | Seed-specific regulation |
| *HvDJB03* | 1565 | 1557 | Seed-specific regulation |
| *HvDJC12* | 1026 | 1021 | Abscisic acid responsive element |
| *HvDJC12* | 966 | 972 | Anaerobic responsive element |
| *HvDJC12* | 1447 | 1441 | Anaerobic responsive element |
| *HvDJC12* | 638 | 647 | Defence responsive element |
| *HvDJC12* | 275 | 268 | Gibberellin-responsive element |
| *HvDJC12* | 1209 | 1216 | Gibberellin-responsive element |
| *HvDJC12* | 1267 | 1260 | Gibberellin-responsive element |
| *HvDJC12* | 1686 | 1680 | Light responsive element |
| *HvDJC12* | 1690 | 1684 | Light responsive element |
| *HvDJC12* | 1023 | 1013 | Light responsive element |
| *HvDJC12* | 1026 | 1032 | Light responsive element |
| *HvDJC12* | 752 | 746 | Light responsive element |
| *HvDJC12* | 962 | 968 | Light responsive element |
| *HvDJC12* | 1671 | 1665 | Light responsive element |
| *HvDJC12* | 1937 | 1931 | Light responsive element |
| *HvDJC12* | 1989 | 1995 | Light responsive element |
| *HvDJC12* | 1993 | 1999 | Light responsive element |
| *HvDJC12* | 1028 | 1023 | MeJA-responsive element |
| *HvDJC12* | 1506 | 1511 | MeJA-responsive element |
| *HvDJC12* | 1785 | 1780 | MeJA-responsive element |
| *HvDJC12* | 275 | 281 | MYB |
| *HvDJC12* | 473 | 479 | MYB |
| *HvDJC12* | 679 | 685 | MYB |
| *HvDJC12* | 753 | 759 | MYB |
| *HvDJC12* | 1210 | 1204 | MYB |
| *HvDJC12* | 1500 | 1494 | MYB |
| *HvDJC12* | 1241 | 1247 | MYB |
| *HvDJC12* | 275 | 281 | MYB |
| *HvDJC12* | 473 | 479 | MYB |
| *HvDJC12* | 1210 | 1204 | MYB |
| *HvDJC12* | 753 | 759 | MYB |
| *HvDJC12* | 719 | 713 | MYC |
| *HvDJC12* | 794 | 800 | MYC |
| *HvDJC12* | 946 | 952 | MYC |
| *HvDJC12* | 1394 | 1400 | MYC |
| *HvDJC12* | 953 | 944 | Salicylic acid responsive element |
| *HvDJC13* | 46 | 52 | Abscisic acid responsive element |
| *HvDJC13* | 47 | 52 | Abscisic acid responsive element |
| *HvDJC13* | 1092 | 1097 | Abscisic acid responsive element |
| *HvDJC13* | 1305 | 1300 | Abscisic acid responsive element |
| *HvDJC13* | 1328 | 1322 | Abscisic acid responsive element |
| *HvDJC13* | 1329 | 1334 | Abscisic acid responsive element |
| *HvDJC13* | 1362 | 1356 | Abscisic acid responsive element |
| *HvDJC13* | 1363 | 1368 | Abscisic acid responsive element |
| *HvDJC13* | 1579 | 1584 | Abscisic acid responsive element |
| *HvDJC13* | 1742 | 1748 | Anaerobic responsive element |
| *HvDJC13* | 760 | 766 | Anoxic responsive element |
| *HvDJC13* | 170 | 176 | Auxin-responsive element |
| *HvDJC13* | 201 | 207 | Auxin-responsive element |
| *HvDJC13* | 1960 | 1954 | Drought responsive element |
| *HvDJC13* | 1698 | 1690 | Light responsive element |
| *HvDJC13* | 1345 | 1339 | Light responsive element |
| *HvDJC13* | 1831 | 1825 | Light responsive element |
| *HvDJC13* | 46 | 52 | Light responsive element |
| *HvDJC13* | 987 | 981 | Light responsive element |
| *HvDJC13* | 997 | 988 | Light responsive element |
| *HvDJC13* | 1091 | 1085 | Light responsive element |
| *HvDJC13* | 1328 | 1322 | Light responsive element |
| *HvDJC13* | 1362 | 1356 | Light responsive element |
| *HvDJC13* | 46 | 52 | Light responsive element |
| *HvDJC13* | 1305 | 1311 | Light responsive element |
| *HvDJC13* | 1328 | 1322 | Light responsive element |
| *HvDJC13* | 1362 | 1356 | Light responsive element |
| *HvDJC13* | 1578 | 1572 | Light responsive element |
| *HvDJC13* | 1577 | 1587 | Light responsive element |
| *HvDJC13* | 1320 | 1329 | Light responsive element |
| *HvDJC13* | 1841 | 1832 | Light responsive element |
| *HvDJC13* | 829 | 817 | Light responsive element |
| *HvDJC13* | 308 | 313 | MeJA-responsive element |
| *HvDJC13* | 1170 | 1165 | MeJA-responsive element |
| *HvDJC13* | 1520 | 1525 | MeJA-responsive element |
| *HvDJC13* | 164 | 170 | MYB |
| *HvDJC13* | 514 | 508 | MYB |
| *HvDJC13* | 943 | 937 | MYB |
| *HvDJC13* | 1402 | 1396 | MYB |
| *HvDJC13* | 1711 | 1705 | MYB |
| *HvDJC13* | 1960 | 1954 | MYB |
| *HvDJC13* | 957 | 963 | MYB |
| *HvDJC13* | 1438 | 1444 | MYB |
| *HvDJC13* | 164 | 170 | MYB |
| *HvDJC13* | 514 | 508 | MYB |
| *HvDJC13* | 1402 | 1396 | MYB |
| *HvDJC13* | 1711 | 1705 | MYB |
| *HvDJC13* | 267 | 261 | MYC |
| *HvDJC14* | 804 | 799 | Abscisic acid responsive element |
| *HvDJC14* | 1293 | 1288 | Abscisic acid responsive element |
| *HvDJC14* | 1867 | 1872 | Abscisic acid responsive element |
| *HvDJC14* | 1866 | 1872 | Abscisic acid responsive element |
| *HvDJC14* | 1866 | 1860 | Abscisic acid responsive element |
| *HvDJC14* | 912 | 919 | Gibberellin-responsive element |
| *HvDJC14* | 517 | 528 | Light responsive element |
| *HvDJC14* | 804 | 810 | Light responsive element |
| *HvDJC14* | 1293 | 1299 | Light responsive element |
| *HvDJC14* | 1866 | 1872 | Light responsive element |
| *HvDJC14* | 172 | 165 | Light responsive element |
| *HvDJC14* | 173 | 167 | Light responsive element |
| *HvDJC14* | 1946 | 1940 | Light responsive element |
| *HvDJC14* | 305 | 312 | Light responsive element |
| *HvDJC14* | 1031 | 1038 | Light responsive element |
| *HvDJC14* | 1136 | 1143 | Light responsive element |
| *HvDJC14* | 1495 | 1488 | Light responsive element |
| *HvDJC14* | 1501 | 1494 | Light responsive element |
| *HvDJC14* | 614 | 609 | MeJA-responsive element |
| *HvDJC14* | 1277 | 1282 | MeJA-responsive element |
| *HvDJC14* | 1601 | 1606 | MeJA-responsive element |
| *HvDJC14* | 1610 | 1615 | MeJA-responsive element |
| *HvDJC14* | 1622 | 1617 | MeJA-responsive element |
| *HvDJC14* | 1640 | 1645 | MeJA-responsive element |
| *HvDJC14* | 1744 | 1749 | MeJA-responsive element |
| *HvDJC14* | 1772 | 1777 | MeJA-responsive element |
| *HvDJC14* | 1822 | 1827 | MeJA-responsive element |
| *HvDJC14* | 705 | 699 | MYB |
| *HvDJC14* | 913 | 907 | MYB |
| *HvDJC14* | 913 | 907 | MYB |
| *HvDJC14* | 701 | 707 | MYC |
| *HvDJC15* | 327 | 332 | Abscisic acid responsive element |
| *HvDJC15* | 1812 | 1817 | Abscisic acid responsive element |
| *HvDJC15* | 1883 | 1892 | Abscisic acid responsive element |
| *HvDJC15* | 1886 | 1891 | Abscisic acid responsive element |
| *HvDJC15* | 1811 | 1817 | Abscisic acid responsive element |
| *HvDJC15* | 1811 | 1805 | Abscisic acid responsive element |
| *HvDJC15* | 1433 | 1427 | Anaerobic responsive element |
| *HvDJC15* | 1440 | 1434 | Anaerobic responsive element |
| *HvDJC15* | 561 | 552 | Circadian |
| *HvDJC15* | 744 | 735 | Defence responsive element |
| *HvDJC15* | 823 | 829 | Drought responsive element |
| *HvDJC15* | 1670 | 1676 | Drought responsive element |
| *HvDJC15* | 905 | 915.5 | Drought responsive element |
| *HvDJC15* | 394 | 401 | Gibberellin-responsive element |
| *HvDJC15* | 1809 | 1800 | Light responsive element |
| *HvDJC15* | 279 | 288 | Light responsive element |
| *HvDJC15* | 1885 | 1879 | Light responsive element |
| *HvDJC15* | 253 | 259 | Light responsive element |
| *HvDJC15* | 326 | 320 | Light responsive element |
| *HvDJC15* | 1811 | 1817 | Light responsive element |
| *HvDJC15* | 912 | 918 | Light responsive element |
| *HvDJC15* | 1123 | 1117 | Light responsive element |
| *HvDJC15* | 448 | 458 | Light responsive element |
| *HvDJC15* | 1141 | 1150 | Light responsive element |
| *HvDJC15* | 1936 | 1930 | Light responsive element |
| *HvDJC15* | 325 | 330 | MeJA-responsive element |
| *HvDJC15* | 114 | 108 | MYB |
| *HvDJC15* | 158 | 152 | MYB |
| *HvDJC15* | 395 | 389 | MYB |
| *HvDJC15* | 911 | 905 | MYB |
| *HvDJC15* | 958 | 964 | MYB |
| *HvDJC15* | 1124 | 1130 | MYB |
| *HvDJC15* | 1462 | 1468 | MYB |
| *HvDJC15* | 823 | 829 | MYB |
| *HvDJC15* | 1670 | 1676 | MYB |
| *HvDJC15* | 1825 | 1831 | MYB |
| *HvDJC15* | 395 | 389 | MYB |
| *HvDJC15* | 958 | 964 | MYB |
| *HvDJC15* | 114 | 108 | MYB |
| *HvDJC15* | 158 | 152 | MYB |
| *HvDJC15* | 911 | 905 | MYB |
| *HvDJC15* | 1124 | 1130 | MYB |
| *HvDJC15* | 1462 | 1468 | MYB |
| *HvDJC15* | 66 | 72 | MYC |
| *HvDJC15* | 73 | 79 | MYC |
| *HvDJC15* | 154 | 160 | MYC |
| *HvDJC15* | 530 | 536 | MYC |
| *HvDJC15* | 656 | 662 | MYC |
| *HvDJC15* | 896 | 890 | MYC |
| *HvDJC15* | 1790 | 1796 | MYC |
| *HvDJC15* | 323 | 332 | Zein metabolism regulation |
| *HvDJC15* | 995 | 986 | Zein metabolism regulation |
| *HvDJC16* | 532 | 537 | Abscisic acid responsive element |
| *HvDJC16* | 1376 | 1369 | Abscisic acid responsive element |
| *HvDJC16* | 1613 | 1608 | Abscisic acid responsive element |
| *HvDJC16* | 1613 | 1607 | Abscisic acid responsive element |
| *HvDJC16* | 1613 | 1619 | Abscisic acid responsive element |
| *HvDJC16* | 1124 | 1118 | Anaerobic responsive element |
| *HvDJC16* | 1353 | 1359 | Auxin-responsive element |
| *HvDJC16* | 1685 | 1679 | Auxin-responsive element |
| *HvDJC16* | 1699 | 1690 | Cell cycle regulation |
| *HvDJC16* | 850 | 841 | Defence responsive element |
| *HvDJC16* | 974 | 980 | Drought responsive element |
| *HvDJC16* | 1915 | 1921 | Drought responsive element |
| *HvDJC16* | 1756 | 1745 | Drought responsive element |
| *HvDJC16* | 1540 | 1530 | Light responsive element |
| *HvDJC16* | 531 | 525 | Light responsive element |
| *HvDJC16* | 1613 | 1607 | Light responsive element |
| *HvDJC16* | 1074 | 1067 | Light responsive element |
| *HvDJC16* | 1075 | 1069 | Light responsive element |
| *HvDJC16* | 725 | 731 | Light responsive element |
| *HvDJC16* | 1926 | 1932 | Low-temperature responsive element |
| *HvDJC16* | 92 | 87 | MeJA-responsive element |
| *HvDJC16* | 530 | 535 | MeJA-responsive element |
| *HvDJC16* | 974 | 980 | MYB |
| *HvDJC16* | 1915 | 1921 | MYB |
| *HvDJC16* | 357 | 351 | MYB |
| *HvDJC16* | 953 | 947 | MYB |
| *HvDJC16* | 357 | 351 | MYB |
| *HvDJC16* | 953 | 947 | MYB |
| *HvDJC16* | 574 | 580 | MYC |
| *HvDJC16* | 1019 | 1025 | MYC |
| *HvDJC17* | 1004 | 1011 | Auxin-responsive element |
| *HvDJC17* | 804 | 798 | Auxin-responsive element |
| *HvDJC17* | 130 | 136 | Drought responsive element |
| *HvDJC17* | 900 | 907 | Endosperm |
| *HvDJC17* | 1013 | 1020 | Gibberellin-responsive element |
| *HvDJC17* | 1172 | 1179 | Gibberellin-responsive element |
| *HvDJC17* | 1403 | 1410 | Gibberellin-responsive element |
| *HvDJC17* | 81 | 73 | Light responsive element |
| *HvDJC17* | 452 | 443 | Light responsive element |
| *HvDJC17* | 1793 | 1787 | Light responsive element |
| *HvDJC17* | 720 | 714 | Light responsive element |
| *HvDJC17* | 417 | 411 | Low-temperature responsive element |
| *HvDJC17* | 788 | 793 | MeJA-responsive element |
| *HvDJC17* | 818 | 813 | MeJA-responsive element |
| *HvDJC17* | 297 | 303 | MYB |
| *HvDJC17* | 483 | 477 | MYB |
| *HvDJC17* | 1173 | 1167 | MYB |
| *HvDJC17* | 130 | 136 | MYB |
| *HvDJC17* | 297 | 303 | MYB |
| *HvDJC17* | 1173 | 1167 | MYB |
| *HvDJC17* | 49 | 43 | MYC |
| *HvDJC17* | 185 | 179 | MYC |
| *HvDJC17* | 860 | 866 | MYC |
| *HvDJC17* | 677 | 686 | Zein metabolism regulation |
| *HvDJC17* | 893 | 902 | Zein metabolism regulation |
| *HvDJC17* | 1017 | 1008 | Zein metabolism regulation |
| *HvDJC17* | 1515 | 1525 | Zein metabolism regulation |
| *HvDJC18* | 140 | 135 | Abscisic acid responsive element |
| *HvDJC18* | 1386 | 1381 | Abscisic acid responsive element |
| *HvDJC18* | 1404 | 1409 | Abscisic acid responsive element |
| *HvDJC18* | 1633 | 1638 | Abscisic acid responsive element |
| *HvDJC18* | 1638 | 1643 | Abscisic acid responsive element |
| *HvDJC18* | 1792 | 1787 | Abscisic acid responsive element |
| *HvDJC18* | 1386 | 1380 | Abscisic acid responsive element |
| *HvDJC18* | 1632 | 1638 | Abscisic acid responsive element |
| *HvDJC18* | 1386 | 1392 | Abscisic acid responsive element |
| *HvDJC18* | 1632 | 1626 | Abscisic acid responsive element |
| *HvDJC18* | 543 | 537 | Anaerobic responsive element |
| *HvDJC18* | 1543 | 1549 | Anaerobic responsive element |
| *HvDJC18* | 1282 | 1276 | Anoxic responsive element |
| *HvDJC18* | 1425 | 1431 | Auxin-responsive element |
| *HvDJC18* | 1450 | 1456 | Auxin-responsive element |
| *HvDJC18* | 199 | 190 | Defence responsive element |
| *HvDJC18* | 1336 | 1343 | Endosperm |
| *HvDJC18* | 1489 | 1498 | Light responsive element |
| *HvDJC18* | 369 | 360 | Light responsive element |
| *HvDJC18* | 140 | 132 | Light responsive element |
| *HvDJC18* | 1386 | 1380 | Light responsive element |
| *HvDJC18* | 1403 | 1397 | Light responsive element |
| *HvDJC18* | 1632 | 1638 | Light responsive element |
| *HvDJC18* | 1637 | 1631 | Light responsive element |
| *HvDJC18* | 1792 | 1798 | Light responsive element |
| *HvDJC18* | 140 | 146 | Light responsive element |
| *HvDJC18* | 291 | 298 | Light responsive element |
| *HvDJC18* | 1926 | 1938 | Light responsive element |
| *HvDJC18* | 199 | 193 | Light responsive element |
| *HvDJC18* | 1082 | 1077 | MeJA-responsive element |
| *HvDJC18* | 1402 | 1407 | MeJA-responsive element |
| *HvDJC18* | 1636 | 1641 | MeJA-responsive element |
| *HvDJC18* | 668 | 674 | MYB |
| *HvDJC18* | 1254 | 1260 | MYB |
| *HvDJC18* | 290 | 284 | MYB |
| *HvDJC18* | 1108 | 1102 | MYB |
| *HvDJC18* | 1352 | 1358 | MYB |
| *HvDJC18* | 1926 | 1932 | MYB |
| *HvDJC18* | 1498 | 1504 | MYB |
| *HvDJC18* | 1352 | 1358 | MYB |
| *HvDJC18* | 290 | 284 | MYB |
| *HvDJC18* | 1108 | 1102 | MYB |
| *HvDJC18* | 828 | 834 | MYC |
| *HvDJC18* | 916 | 910 | MYC |
| *HvDJC18* | 1329 | 1337 | Zein metabolism regulation |
| *HvDJC18* | 1400 | 1409 | Zein metabolism regulation |
| *HvDJC18* | 1706 | 1714 | Zein metabolism regulation |
| *HvDJC19* | 373 | 379 | Anaerobic responsive element |
| *HvDJC19* | 1554 | 1560 | Anaerobic responsive element |
| *HvDJC19* | 1654 | 1660 | Anaerobic responsive element |
| *HvDJC19* | 1950 | 1944 | Anaerobic responsive element |
| *HvDJC19* | 693 | 699 | Anoxic responsive element |
| *HvDJC19* | 730 | 737 | Auxin-responsive element |
| *HvDJC19* | 505 | 498 | Gibberellin-responsive element |
| *HvDJC19* | 1488 | 1495 | Gibberellin-responsive element |
| *HvDJC19* | 124 | 130 | Light responsive element |
| *HvDJC19* | 142 | 148 | Light responsive element |
| *HvDJC19* | 1374 | 1368 | Light responsive element |
| *HvDJC19* | 1985 | 1977 | Light responsive element |
| *HvDJC19* | 1873 | 1879 | Light responsive element |
| *HvDJC19* | 1893 | 1899 | Light responsive element |
| *HvDJC19* | 1913 | 1919 | Light responsive element |
| *HvDJC19* | 1869 | 1859 | Light responsive element |
| *HvDJC19* | 1889 | 1879 | Light responsive element |
| *HvDJC19* | 1909 | 1899 | Light responsive element |
| *HvDJC19* | 1840 | 1834 | Light responsive element |
| *HvDJC19* | 1206 | 1200 | Light responsive element |
| *HvDJC19* | 1515 | 1509 | Light responsive element |
| *HvDJC19* | 699 | 693 | Low-temperature responsive element |
| *HvDJC19* | 856 | 850 | Low-temperature responsive element |
| *HvDJC19* | 788 | 794 | MYB |
| *HvDJC19* | 797 | 791 | MYB |
| *HvDJC19* | 1807 | 1813 | MYB |
| *HvDJC19* | 1658 | 1652 | MYB |
| *HvDJC19* | 841 | 847 | MYB |
| *HvDJC19* | 1470 | 1476 | MYB |
| *HvDJC19* | 797 | 791 | MYB |
| *HvDJC19* | 1807 | 1813 | MYB |
| *HvDJC19* | 110 | 104 | MYC |
| *HvDJC19* | 1258 | 1264 | MYC |
| *HvDJC20* | 1243 | 1236 | Abscisic acid responsive element |
| *HvDJC20* | 423 | 417 | Anaerobic responsive element |
| *HvDJC20* | 1755 | 1749 | Anaerobic responsive element |
| *HvDJC20* | 742 | 736 | Anoxic responsive element |
| *HvDJC20* | 762 | 756 | Anoxic responsive element |
| *HvDJC20* | 1067 | 1061 | Anoxic responsive element |
| *HvDJC20* | 1993 | 2000 | Gibberellin-responsive element |
| *HvDJC20* | 602 | 608 | Light responsive element |
| *HvDJC20* | 1482 | 1476 | Light responsive element |
| *HvDJC20* | 1700 | 1694 | Light responsive element |
| *HvDJC20* | 307 | 301 | Light responsive element |
| *HvDJC20* | 642 | 636 | Light responsive element |
| *HvDJC20* | 562 | 556 | Light responsive element |
| *HvDJC20* | 1090 | 1084 | Light responsive element |
| *HvDJC20* | 1497 | 1503 | Light responsive element |
| *HvDJC20* | 163 | 156 | Light responsive element |
| *HvDJC20* | 1032 | 1038 | Low-temperature responsive element |
| *HvDJC20* | 1723 | 1729 | Low-temperature responsive element |
| *HvDJC20* | 576 | 571 | MeJA-responsive element |
| *HvDJC20* | 1204 | 1209 | MeJA-responsive element |
| *HvDJC20* | 1321 | 1326 | MeJA-responsive element |
| *HvDJC20* | 1645 | 1650 | MeJA-responsive element |
| *HvDJC20* | 288 | 282 | MYB |
| *HvDJC20* | 1920 | 1926 | MYB |
| *HvDJC20* | 1994 | 1988 | MYB |
| *HvDJC20* | 1920 | 1926 | MYB |
| *HvDJC20* | 1994 | 1988 | MYB |
| *HvDJC20* | 178 | 172 | MYC |
| *HvDJC20* | 554 | 560 | MYC |
| *HvDJC20* | 785 | 779 | MYC |
| *HvDJC20* | 944 | 950 | MYC |
| *HvDJC20* | 1864 | 1870 | MYC |
| *HvDJC20* | 120 | 129 | Zein metabolism regulation |
| *HvDJC21* | 1454 | 1449 | Abscisic acid responsive element |
| *HvDJC21* | 176 | 182 | Anoxic responsive element |
| *HvDJC21* | 190 | 196 | Anoxic responsive element |
| *HvDJC21* | 468 | 474 | Anoxic responsive element |
| *HvDJC21* | 1847 | 1853 | Anoxic responsive element |
| *HvDJC21* | 462 | 468 | Drought responsive element |
| *HvDJC21* | 519 | 525 | Drought responsive element |
| *HvDJC21* | 580 | 586 | Drought responsive element |
| *HvDJC21* | 888 | 882 | Drought responsive element |
| *HvDJC21* | 1297 | 1290 | Gibberellin-responsive element |
| *HvDJC21* | 952 | 960 | Light responsive element |
| *HvDJC21* | 927 | 936.5 | Light responsive element |
| *HvDJC21* | 356 | 350 | Light responsive element |
| *HvDJC21* | 1454 | 1460 | Light responsive element |
| *HvDJC21* | 636 | 626 | Light responsive element |
| *HvDJC21* | 236 | 230 | Light responsive element |
| *HvDJC21* | 1800 | 1794 | Light responsive element |
| *HvDJC21* | 1926 | 1920 | Light responsive element |
| *HvDJC21* | 179 | 185 | Low-temperature responsive element |
| *HvDJC21* | 1491 | 1497 | Low-temperature responsive element |
| *HvDJC21* | 322 | 317 | MeJA-responsive element |
| *HvDJC21* | 857 | 852 | MeJA-responsive element |
| *HvDJC21* | 1644 | 1639 | MeJA-responsive element |
| *HvDJC21* | 1239 | 1232 | MYB |
| *HvDJC21* | 462 | 468 | MYB |
| *HvDJC21* | 519 | 525 | MYB |
| *HvDJC21* | 580 | 586 | MYB |
| *HvDJC21* | 888 | 882 | MYB |
| *HvDJC21* | 882 | 888 | MYB |
| *HvDJC21* | 1297 | 1303 | MYB |
| *HvDJC21* | 1613 | 1619 | MYB |
| *HvDJC21* | 1191 | 1185 | MYB |
| *HvDJC21* | 1297 | 1303 | MYB |
| *HvDJC21* | 1613 | 1619 | MYB |
| *HvDJC21* | 1650 | 1657 | MYC |
| *HvDJC21* | 927 | 921 | MYC |
| *HvDJC21* | 1119 | 1125 | MYC |
| *HvDJC21* | 1287 | 1281 | MYC |
| *HvDJC21* | 1429 | 1423 | MYC |
| *HvDJC22* | 237 | 232 | Abscisic acid responsive element |
| *HvDJC22* | 1848 | 1853 | Abscisic acid responsive element |
| *HvDJC22* | 1925 | 1930 | Abscisic acid responsive element |
| *HvDJC22* | 1847 | 1853 | Abscisic acid responsive element |
| *HvDJC22* | 1847 | 1841 | Abscisic acid responsive element |
| *HvDJC22* | 856 | 850 | Anaerobic responsive element |
| *HvDJC22* | 988 | 982 | Anaerobic responsive element |
| *HvDJC22* | 1306 | 1300 | Anaerobic responsive element |
| *HvDJC22* | 458 | 464 | Drought responsive element |
| *HvDJC22* | 1581 | 1575 | Light responsive element |
| *HvDJC22* | 1643 | 1650 | Light responsive element |
| *HvDJC22* | 237 | 243 | Light responsive element |
| *HvDJC22* | 1847 | 1853 | Light responsive element |
| *HvDJC22* | 1924 | 1918 | Light responsive element |
| *HvDJC22* | 1129 | 1123 | Light responsive element |
| *HvDJC22* | 343 | 333 | Light responsive element |
| *HvDJC22* | 1526 | 1516 | Light responsive element |
| *HvDJC22* | 179 | 174 | MeJA-responsive element |
| *HvDJC22* | 242 | 237 | MeJA-responsive element |
| *HvDJC22* | 413 | 419 | MYB |
| *HvDJC22* | 799 | 793 | MYB |
| *HvDJC22* | 1240 | 1246 | MYB |
| *HvDJC22* | 1248 | 1254 | MYB |
| *HvDJC22* | 1328 | 1334 | MYB |
| *HvDJC22* | 458 | 464 | MYB |
| *HvDJC22* | 682 | 688 | MYB |
| *HvDJC22* | 843 | 849 | MYC |
| *HvDJC22* | 981 | 975 | MYC |
| *HvDJC22* | 1370 | 1364 | MYC |
| *HvDJC22* | 1463 | 1457 | MYC |
| *HvDJC23* | 165 | 170 | Abscisic acid responsive element |
| *HvDJC23* | 732 | 727 | Abscisic acid responsive element |
| *HvDJC23* | 1216 | 1211 | Abscisic acid responsive element |
| *HvDJC23* | 1234 | 1229 | Abscisic acid responsive element |
| *HvDJC23* | 1312 | 1306 | Abscisic acid responsive element |
| *HvDJC23* | 1313 | 1318 | Abscisic acid responsive element |
| *HvDJC23* | 732 | 726 | Abscisic acid responsive element |
| *HvDJC23* | 732 | 738 | Abscisic acid responsive element |
| *HvDJC23* | 105 | 99 | Anaerobic responsive element |
| *HvDJC23* | 338 | 332 | Anaerobic responsive element |
| *HvDJC23* | 728 | 734 | Anaerobic responsive element |
| *HvDJC23* | 890 | 896 | Anaerobic responsive element |
| *HvDJC23* | 1143 | 1137 | Anaerobic responsive element |
| *HvDJC23* | 1230 | 1236 | Anaerobic responsive element |
| *HvDJC23* | 1337 | 1343 | Anaerobic responsive element |
| *HvDJC23* | 1957 | 1951 | Auxin-responsive element |
| *HvDJC23* | 164 | 158 | Light responsive element |
| *HvDJC23* | 1234 | 1240 | Light responsive element |
| *HvDJC23* | 1312 | 1306 | Light responsive element |
| *HvDJC23* | 732 | 726 | Light responsive element |
| *HvDJC23* | 1216 | 1222 | Light responsive element |
| *HvDJC23* | 1312 | 1306 | Light responsive element |
| *HvDJC23* | 240 | 234 | Light responsive element |
| *HvDJC23* | 222 | 232 | Light responsive element |
| *HvDJC23* | 1442 | 1451 | Light responsive element |
| *HvDJC23* | 704 | 710 | Low-temperature responsive element |
| *HvDJC23* | 857 | 851 | Low-temperature responsive element |
| *HvDJC23* | 877 | 871 | Low-temperature responsive element |
| *HvDJC23* | 1359 | 1353 | Low-temperature responsive element |
| *HvDJC23* | 830 | 835 | MeJA-responsive element |
| *HvDJC23* | 1375 | 1382 | MYB |
| *HvDJC23* | 241 | 247 | MYB |
| *HvDJC23* | 360 | 354 | MYB |
| *HvDJC23* | 526 | 532 | MYB |
| *HvDJC23* | 676 | 670 | MYB |
| *HvDJC23* | 1513 | 1507 | MYB |
| *HvDJC23* | 241 | 247 | MYB |
| *HvDJC23* | 360 | 354 | MYB |
| *HvDJC23* | 213 | 219 | MYC |
| *HvDJC23* | 626 | 620 | MYC |
| *HvDJC23* | 826 | 820 | MYC |
| *HvDJC23* | 212 | 203 | Zein metabolism regulation |
| *HvDJC23* | 1569 | 1578 | Zein metabolism regulation |
| *HvDJC24* | 955 | 948 | Abscisic acid responsive element |
| *HvDJC24* | 1329 | 1323 | Abscisic acid responsive element |
| *HvDJC24* | 1330 | 1335 | Abscisic acid responsive element |
| *HvDJC24* | 275 | 281 | Anaerobic responsive element |
| *HvDJC24* | 992 | 998 | Anaerobic responsive element |
| *HvDJC24* | 522 | 515 | Auxin-responsive element |
| *HvDJC24* | 1339 | 1333 | Auxin-responsive element |
| *HvDJC24* | 1411 | 1405 | Drought responsive element |
| *HvDJC24* | 707 | 701 | Light responsive element |
| *HvDJC24* | 1329 | 1323 | Light responsive element |
| *HvDJC24* | 1329 | 1323 | Light responsive element |
| *HvDJC24* | 1532 | 1522 | Light responsive element |
| *HvDJC24* | 1175 | 1180 | MeJA-responsive element |
| *HvDJC24* | 1351 | 1346 | MeJA-responsive element |
| *HvDJC24* | 1465 | 1470 | MeJA-responsive element |
| *HvDJC24* | 1711 | 1716 | MeJA-responsive element |
| *HvDJC24* | 1714 | 1709 | MeJA-responsive element |
| *HvDJC24* | 1743 | 1748 | MeJA-responsive element |
| *HvDJC24* | 1746 | 1741 | MeJA-responsive element |
| *HvDJC24* | 1810 | 1805 | MeJA-responsive element |
| *HvDJC24* | 1702 | 1695 | MYB |
| *HvDJC24* | 132 | 138 | MYB |
| *HvDJC24* | 530 | 524 | MYB |
| *HvDJC24* | 548 | 554 | MYB |
| *HvDJC24* | 597 | 591 | MYB |
| *HvDJC24* | 711 | 705 | MYB |
| *HvDJC24* | 778 | 772 | MYB |
| *HvDJC24* | 910 | 916 | MYB |
| *HvDJC24* | 1070 | 1076 | MYB |
| *HvDJC24* | 1684 | 1678 | MYB |
| *HvDJC24* | 594 | 600 | MYB |
| *HvDJC24* | 775 | 781 | MYB |
| *HvDJC24* | 1411 | 1405 | MYB |
| *HvDJC24* | 889 | 895 | MYB |
| *HvDJC24* | 132 | 138 | MYB |
| *HvDJC24* | 597 | 591 | MYB |
| *HvDJC24* | 778 | 772 | MYB |
| *HvDJC24* | 1070 | 1076 | MYB |
| *HvDJC24* | 1684 | 1678 | MYB |
| *HvDJC24* | 530 | 524 | MYB |
| *HvDJC24* | 711 | 705 | MYB |
| *HvDJC24* | 422 | 416 | MYC |
| *HvDJC24* | 603 | 597 | MYC |
| *HvDJC24* | 784 | 778 | MYC |
| *HvDJC24* | 1171 | 1177 | MYC |
| *HvDJC24* | 1759 | 1765 | MYC |
| *HvDJC24* | 198 | 188 | Salicylic acid responsive element |
| *HvDJC25* | 274 | 267 | Abscisic acid responsive element |
| *HvDJC25* | 1017 | 1011 | Abscisic acid responsive element |
| *HvDJC25* | 1018 | 1023 | Abscisic acid responsive element |
| *HvDJC25* | 1577 | 1582 | Abscisic acid responsive element |
| *HvDJC25* | 1023 | 1017 | Anaerobic responsive element |
| *HvDJC25* | 1754 | 1748 | Anaerobic responsive element |
| *HvDJC25* | 943 | 937 | Auxin-responsive element |
| *HvDJC25* | 1239 | 1233 | Auxin-responsive element |
| *HvDJC25* | 1388 | 1382 | Auxin-responsive element |
| *HvDJC25* | 1242 | 1251 | Defence responsive element |
| *HvDJC25* | 302 | 296 | Drought responsive element |
| *HvDJC25* | 1007 | 1014 | Gibberellin-responsive element |
| *HvDJC25* | 295 | 286 | Light responsive element |
| *HvDJC25* | 1014 | 1005 | Light responsive element |
| *HvDJC25* | 1017 | 1011 | Light responsive element |
| *HvDJC25* | 589 | 583 | Light responsive element |
| *HvDJC25* | 1017 | 1011 | Light responsive element |
| *HvDJC25* | 1105 | 1099 | Light responsive element |
| *HvDJC25* | 1576 | 1570 | Light responsive element |
| *HvDJC25* | 952 | 958 | Light responsive element |
| *HvDJC25* | 1096 | 1087 | Light responsive element |
| *HvDJC25* | 865 | 872 | Light responsive element |
| *HvDJC25* | 1894 | 1901 | Light responsive element |
| *HvDJC25* | 327 | 333 | Light responsive element |
| *HvDJC25* | 1781 | 1786 | MeJA-responsive element |
| *HvDJC25* | 302 | 296 | MYB |
| *HvDJC25* | 680 | 674 | MYB |
| *HvDJC25* | 1737 | 1743 | MYB |
| *HvDJC25* | 416 | 410 | MYB |
| *HvDJC25* | 951 | 945 | MYB |
| *HvDJC25* | 1377 | 1371 | MYB |
| *HvDJC25* | 775 | 781 | MYB |
| *HvDJC25* | 416 | 410 | MYB |
| *HvDJC25* | 951 | 945 | MYB |
| *HvDJC25* | 725 | 718 | MYC |
| *HvDJC25* | 192 | 198 | MYC |
| *HvDJC25* | 563 | 569 | MYC |
| *HvDJC25* | 786 | 792 | MYC |
| *HvDJC25* | 1760 | 1754 | MYC |
| *HvDJC25* | 234 | 225 | Salicylic acid responsive element |
| *HvDJC26* | 1930 | 1925 | Abscisic acid responsive element |
| *HvDJC26* | 507 | 513 | Anaerobic responsive element |
| *HvDJC26* | 883 | 877 | Anaerobic responsive element |
| *HvDJC26* | 917 | 911 | Anaerobic responsive element |
| *HvDJC26* | 481 | 487 | Auxin-responsive element |
| *HvDJC26* | 1725 | 1731 | Drought responsive element |
| *HvDJC26* | 245 | 238 | Gibberellin-responsive element |
| *HvDJC26* | 695 | 688 | Gibberellin-responsive element |
| *HvDJC26* | 729 | 736 | Gibberellin-responsive element |
| *HvDJC26* | 1505 | 1499 | Light responsive element |
| *HvDJC26* | 800 | 808 | Light responsive element |
| *HvDJC26* | 764 | 770 | Light responsive element |
| *HvDJC26* | 855 | 861 | Light responsive element |
| *HvDJC26* | 1930 | 1936 | Light responsive element |
| *HvDJC26* | 2010 | 2004 | Light responsive element |
| *HvDJC26* | 602 | 609 | Light responsive element |
| *HvDJC26* | 255 | 249 | Low-temperature responsive element |
| *HvDJC26* | 598 | 592 | Low-temperature responsive element |
| *HvDJC26* | 1695 | 1689 | Low-temperature responsive element |
| *HvDJC26* | 202 | 197 | MeJA-responsive element |
| *HvDJC26* | 302 | 307 | MeJA-responsive element |
| *HvDJC26* | 1127 | 1134 | MYB |
| *HvDJC26* | 291 | 285 | MYB |
| *HvDJC26* | 632 | 626 | MYB |
| *HvDJC26* | 1725 | 1731 | MYB |
| *HvDJC26* | 245 | 251 | MYB |
| *HvDJC26* | 695 | 701 | MYB |
| *HvDJC26* | 730 | 724 | MYB |
| *HvDJC26* | 245 | 251 | MYB |
| *HvDJC26* | 695 | 701 | MYB |
| *HvDJC26* | 730 | 724 | MYB |
| *HvDJC26* | 517 | 523 | MYC |
| *HvDJC26* | 583 | 577 | MYC |
| *HvDJC26* | 1732 | 1726 | MYC |
| *HvDJC26* | 1473 | 1465 | Seed-specific regulation |
| *HvDJC26* | 119 | 110 | Zein metabolism regulation |
| *HvDJC26* | 165 | 174 | Zein metabolism regulation |
| *HvDJB04* | 431 | 436 | Abscisic acid responsive element |
| *HvDJB04* | 922 | 927 | Abscisic acid responsive element |
| *HvDJB04* | 1725 | 1720 | Abscisic acid responsive element |
| *HvDJB04* | 1775 | 1782 | Abscisic acid responsive element |
| *HvDJB04* | 1903 | 1908 | Abscisic acid responsive element |
| *HvDJB04* | 921 | 927 | Abscisic acid responsive element |
| *HvDJB04* | 1725 | 1719 | Abscisic acid responsive element |
| *HvDJB04* | 921 | 915 | Abscisic acid responsive element |
| *HvDJB04* | 1725 | 1731 | Abscisic acid responsive element |
| *HvDJB04* | 78 | 72 | Anaerobic responsive element |
| *HvDJB04* | 747 | 741 | Anaerobic responsive element |
| *HvDJB04* | 1521 | 1527 | Anaerobic responsive element |
| *HvDJB04* | 692 | 698 | Anoxic responsive element |
| *HvDJB04* | 1551 | 1545 | Anoxic responsive element |
| *HvDJB04* | 1619 | 1612 | Gibberellin-responsive element |
| *HvDJB04* | 1723 | 1732 | Light responsive element |
| *HvDJB04* | 706 | 698 | Light responsive element |
| *HvDJB04* | 992 | 1000 | Light responsive element |
| *HvDJB04* | 1424 | 1418 | Light responsive element |
| *HvDJB04* | 212 | 218 | Light responsive element |
| *HvDJB04* | 430 | 424 | Light responsive element |
| *HvDJB04* | 639 | 633 | Light responsive element |
| *HvDJB04* | 921 | 927 | Light responsive element |
| *HvDJB04* | 1725 | 1719 | Light responsive element |
| *HvDJB04* | 1902 | 1896 | Light responsive element |
| *HvDJB04* | 823 | 829 | Light responsive element |
| *HvDJB04* | 1402 | 1396 | Light responsive element |
| *HvDJB04* | 1973 | 1979 | Light responsive element |
| *HvDJB04* | 1864 | 1857 | Light responsive element |
| *HvDJB04* | 1891 | 1884 | Light responsive element |
| *HvDJB04* | 507 | 502 | MeJA-responsive element |
| *HvDJB04* | 1314 | 1319 | MeJA-responsive element |
| *HvDJB04* | 1798 | 1793 | MeJA-responsive element |
| *HvDJB04* | 289 | 283 | MYB |
| *HvDJB04* | 743 | 737 | MYB |
| *HvDJB04* | 1437 | 1443 | MYB |
| *HvDJB04* | 743 | 737 | MYB |
| *HvDJB04* | 289 | 283 | MYB |
| *HvDJB04* | 1437 | 1443 | MYB |
| *HvDJB04* | 330 | 324 | MYC |
| *HvDJB04* | 489 | 495 | MYC |
| *HvDJB04* | 632 | 626 | MYC |
| *HvDJC27* | 127 | 122 | Abscisic acid responsive element |
| *HvDJC27* | 285 | 280 | Abscisic acid responsive element |
| *HvDJC27* | 439 | 444 | Abscisic acid responsive element |
| *HvDJC27* | 628 | 637 | Abscisic acid responsive element |
| *HvDJC27* | 705 | 714 | Abscisic acid responsive element |
| *HvDJC27* | 1474 | 1469 | Abscisic acid responsive element |
| *HvDJC27* | 1780 | 1789 | Abscisic acid responsive element |
| *HvDJC27* | 1782 | 1776 | Abscisic acid responsive element |
| *HvDJC27* | 1783 | 1788 | Abscisic acid responsive element |
| *HvDJC27* | 1792 | 1787 | Abscisic acid responsive element |
| *HvDJC27* | 1941 | 1936 | Abscisic acid responsive element |
| *HvDJC27* | 438 | 444 | Abscisic acid responsive element |
| *HvDJC27* | 438 | 432 | Abscisic acid responsive element |
| *HvDJC27* | 1948 | 1954 | Anoxic responsive element |
| *HvDJC27* | 491 | 485 | Auxin-responsive element |
| *HvDJC27* | 294 | 285 | Defence responsive element |
| *HvDJC27* | 1723 | 1716 | Gibberellin-responsive element |
| *HvDJC27* | 1599 | 1606 | Gibberellin-responsive element |
| *HvDJC27* | 1868 | 1877 | Light responsive element |
| *HvDJC27* | 435 | 447 | Light responsive element |
| *HvDJC27* | 376 | 384 | Light responsive element |
| *HvDJC27* | 256 | 246 | Light responsive element |
| *HvDJC27* | 127 | 133 | Light responsive element |
| *HvDJC27* | 438 | 444 | Light responsive element |
| *HvDJC27* | 1782 | 1776 | Light responsive element |
| *HvDJC27* | 1939 | 1930 | Light responsive element |
| *HvDJC27* | 1941 | 1947 | Light responsive element |
| *HvDJC27* | 285 | 291 | Light responsive element |
| *HvDJC27* | 1474 | 1480 | Light responsive element |
| *HvDJC27* | 1782 | 1776 | Light responsive element |
| *HvDJC27* | 1792 | 1798 | Light responsive element |
| *HvDJC27* | 113 | 119 | Light responsive element |
| *HvDJC27* | 1973 | 1967 | Light responsive element |
| *HvDJC27* | 2007 | 2001 | Light responsive element |
| *HvDJC27* | 129 | 124 | MeJA-responsive element |
| *HvDJC27* | 1510 | 1517 | MYB |
| *HvDJC27* | 1687 | 1681 | MYB |
| *HvDJC27* | 37 | 31 | MYC |
| *HvDJC27* | 663 | 657 | MYC |
| *HvDJC27* | 740 | 734 | MYC |
| *HvDJC27* | 1141 | 1147 | MYC |
| *HvDJC27* | 288 | 297 | Zein metabolism regulation |
| *HvDJC28* | 523 | 528 | Abscisic acid responsive element |
| *HvDJC28* | 810 | 815 | Abscisic acid responsive element |
| *HvDJC28* | 924 | 919 | Abscisic acid responsive element |
| *HvDJC28* | 994 | 985 | Abscisic acid responsive element |
| *HvDJC28* | 1908 | 1913 | Abscisic acid responsive element |
| *HvDJC28* | 2001 | 1992 | Abscisic acid responsive element |
| *HvDJC28* | 437 | 446 | Anoxic responsive element |
| *HvDJC28* | 1732 | 1740.5 | Cell cycle regulation |
| *HvDJC28* | 988 | 981 | Gibberellin-responsive element |
| *HvDJC28* | 192 | 198 | Light responsive element |
| *HvDJC28* | 924 | 930 | Light responsive element |
| *HvDJC28* | 1907 | 1901 | Light responsive element |
| *HvDJC28* | 522 | 516 | Light responsive element |
| *HvDJC28* | 809 | 803 | Light responsive element |
| *HvDJC28* | 2001 | 1992 | Light responsive element |
| *HvDJC28* | 1691 | 1700 | Light responsive element |
| *HvDJC28* | 815 | 821 | Light responsive element |
| *HvDJC28* | 1912 | 1918 | Light responsive element |
| *HvDJC28* | 904 | 899 | MeJA-responsive element |
| *HvDJC28* | 680 | 686 | MYB |
| *HvDJC28* | 1007 | 1001 | MYB |
| *HvDJC28* | 1734 | 1728 | MYB |
| *HvDJC28* | 1121 | 1115 | MYC |
| *HvDJC28* | 1766 | 1772 | MYC |
| *HvDJC28* | 670 | 661 | Zein metabolism regulation |
| *HvDJC28* | 1822 | 1831 | Zein metabolism regulation |
| *HvDJC29* | 860 | 865 | Abscisic acid responsive element |
| *HvDJC29* | 1835 | 1830 | Abscisic acid responsive element |
| *HvDJC29* | 1667 | 1661 | Anaerobic responsive element |
| *HvDJC29* | 344 | 350 | Auxin-responsive element |
| *HvDJC29* | 1648 | 1641 | Gibberellin-responsive element |
| *HvDJC29* | 1296 | 1290 | Light responsive element |
| *HvDJC29* | 1584 | 1593 | Light responsive element |
| *HvDJC29* | 313 | 306 | Light responsive element |
| *HvDJC29* | 320 | 327 | Light responsive element |
| *HvDJC29* | 1849 | 1840 | Light responsive element |
| *HvDJC29* | 859 | 853 | Light responsive element |
| *HvDJC29* | 1835 | 1841 | Light responsive element |
| *HvDJC29* | 1502 | 1508 | Light responsive element |
| *HvDJC29* | 890 | 885 | MeJA-responsive element |
| *HvDJC29* | 1683 | 1690 | MYB |
| *HvDJC29* | 154 | 160 | MYB |
| *HvDJC29* | 280 | 286 | MYB |
| *HvDJC29* | 1687 | 1693 | MYB |
| *HvDJC29* | 154 | 160 | MYB |
| *HvDJC29* | 1687 | 1693 | MYB |
| *HvDJC29* | 327 | 333 | MYC |
| *HvDJC29* | 1584 | 1578 | MYC |
| *HvDJC29* | 1546 | 1537 | Zein metabolism regulation |
| *HvDJC30* | 353 | 358 | Abscisic acid responsive element |
| *HvDJC30* | 445 | 440 | Abscisic acid responsive element |
| *HvDJC30* | 562 | 568 | Abscisic acid responsive element |
| *HvDJC30* | 563 | 568 | Abscisic acid responsive element |
| *HvDJC30* | 1127 | 1132 | Abscisic acid responsive element |
| *HvDJC30* | 1389 | 1380 | Abscisic acid responsive element |
| *HvDJC30* | 352 | 358 | Abscisic acid responsive element |
| *HvDJC30* | 352 | 346 | Abscisic acid responsive element |
| *HvDJC30* | 115 | 108 | Auxin-responsive element |
| *HvDJC30* | 912 | 918 | Auxin-responsive element |
| *HvDJC30* | 1410 | 1416 | Auxin-responsive element |
| *HvDJC30* | 361 | 368 | Gibberellin-responsive element |
| *HvDJC30* | 445 | 451 | Light responsive element |
| *HvDJC30* | 562 | 568 | Light responsive element |
| *HvDJC30* | 352 | 358 | Light responsive element |
| *HvDJC30* | 562 | 568 | Light responsive element |
| *HvDJC30* | 711 | 717 | Light responsive element |
| *HvDJC30* | 1126 | 1120 | Light responsive element |
| *HvDJC30* | 185 | 191 | Light responsive element |
| *HvDJC30* | 1957 | 1951 | Light responsive element |
| *HvDJC30* | 462 | 469 | Light responsive element |
| *HvDJC30* | 1036 | 1042 | Light responsive element |
| *HvDJC30* | 1077 | 1083 | Light responsive element |
| *HvDJC30* | 1697 | 1702 | MeJA-responsive element |
| *HvDJC30* | 606 | 600 | MYB |
| *HvDJC30* | 1844 | 1838 | MYB |
| *HvDJC30* | 1905 | 1899 | MYB |
| *HvDJC30* | 345 | 351 | MYB |
| *HvDJC30* | 950 | 956 | MYB |
| *HvDJC30* | 259 | 265 | MYC |
| *HvDJC30* | 545 | 551 | MYC |
| *HvDJC30* | 818 | 824 | MYC |
| *HvDJC30* | 1391 | 1385 | MYC |
| *HvDJC30* | 1624 | 1618 | MYC |
| *HvDJC30* | 1924 | 1930 | MYC |
| *HvDJC31* | 450 | 445 | Abscisic acid responsive element |
| *HvDJC31* | 1422 | 1416 | Abscisic acid responsive element |
| *HvDJC31* | 1423 | 1428 | Abscisic acid responsive element |
| *HvDJC31* | 906 | 900 | Auxin-responsive element |
| *HvDJC31* | 1717 | 1711 | Auxin-responsive element |
| *HvDJC31* | 1603 | 1597 | Drought responsive element |
| *HvDJC31* | 1451 | 1444 | Endosperm |
| *HvDJC31* | 762 | 768 | Light responsive element |
| *HvDJC31* | 1455 | 1447 | Light responsive element |
| *HvDJC31* | 157 | 150 | Light responsive element |
| *HvDJC31* | 205 | 212 | Light responsive element |
| *HvDJC31* | 21 | 15 | Light responsive element |
| *HvDJC31* | 450 | 456 | Light responsive element |
| *HvDJC31* | 1422 | 1416 | Light responsive element |
| *HvDJC31* | 1422 | 1416 | Light responsive element |
| *HvDJC31* | 1350 | 1344 | Light responsive element |
| *HvDJC31* | 1800 | 1794 | Light responsive element |
| *HvDJC31* | 317 | 311 | Low-temperature responsive element |
| *HvDJC31* | 756 | 761 | MeJA-responsive element |
| *HvDJC31* | 1120 | 1115 | MeJA-responsive element |
| *HvDJC31* | 260 | 254 | MYB |
| *HvDJC31* | 1603 | 1597 | MYB |
| *HvDJC31* | 242 | 248 | MYB |
| *HvDJC31* | 270 | 264 | MYB |
| *HvDJC31* | 779 | 785 | MYB |
| *HvDJC31* | 963 | 969 | MYB |
| *HvDJC31* | 1447 | 1441 | MYB |
| *HvDJC31* | 1765 | 1771 | MYB |
| *HvDJC31* | 270 | 264 | MYB |
| *HvDJC31* | 779 | 785 | MYB |
| *HvDJC31* | 278 | 284 | MYC |
| *HvDJC31* | 611 | 605 | MYC |
| *HvDJC31* | 947 | 953 | MYC |
| *HvDJC31* | 1384 | 1378 | MYC |
| *HvDJC31* | 1440 | 1449 | Zein metabolism regulation |
| *HvDJC32* | 288 | 282 | Anaerobic responsive element |
| *HvDJC32* | 1987 | 1981 | Anaerobic responsive element |
| *HvDJC32* | 125 | 119 | Anoxic responsive element |
| *HvDJC32* | 255 | 261 | Anoxic responsive element |
| *HvDJC32* | 1697 | 1685 | Gibberellin-responsive element |
| *HvDJC32* | 1589 | 1597 | Light responsive element |
| *HvDJC32* | 1357 | 1351 | Light responsive element |
| *HvDJC32* | 1735 | 1729 | Light responsive element |
| *HvDJC32* | 1675 | 1667 | Light responsive element |
| *HvDJC32* | 1646 | 1657 | Light responsive element |
| *HvDJC32* | 1592 | 1582 | Light responsive element |
| *HvDJC32* | 1715 | 1708 | Light responsive element |
| *HvDJC32* | 418 | 424 | Light responsive element |
| *HvDJC32* | 645 | 651 | Light responsive element |
| *HvDJC32* | 1913 | 1906 | Light responsive element |
| *HvDJC32* | 1937 | 1943 | Light responsive element |
| *HvDJC32* | 187 | 192 | MeJA-responsive element |
| *HvDJC32* | 217 | 222 | MeJA-responsive element |
| *HvDJC32* | 1270 | 1275 | MeJA-responsive element |
| *HvDJC32* | 725 | 718 | MYB |
| *HvDJC32* | 996 | 1002 | MYB |
| *HvDJC32* | 441 | 435 | MYB |
| *HvDJC32* | 1660 | 1666 | MYB |
| *HvDJC32* | 1983 | 1977 | MYB |
| *HvDJC32* | 632 | 626 | MYB |
| *HvDJC32* | 1660 | 1666 | MYB |
| *HvDJC32* | 1266 | 1260 | MYC |
| *HvDJC32* | 1520 | 1514 | MYC |
| *HvDJC32* | 575 | 584 | Salicylic acid responsive element |
| *HvDJC32* | 655 | 664 | Zein metabolism regulation |
| *HvDJC33* | 452 | 447 | Abscisic acid responsive element |
| *HvDJC33* | 452 | 446 | Abscisic acid responsive element |
| *HvDJC33* | 452 | 458 | Abscisic acid responsive element |
| *HvDJC33* | 841 | 835 | Anaerobic responsive element |
| *HvDJC33* | 1053 | 1059 | Anaerobic responsive element |
| *HvDJC33* | 210 | 201 | Circadian |
| *HvDJC33* | 695 | 701 | Drought responsive element |
| *HvDJC33* | 207 | 200 | Gibberellin-responsive element |
| *HvDJC33* | 1770 | 1760 | Light responsive element |
| *HvDJC33* | 1587 | 1595 | Light responsive element |
| *HvDJC33* | 960 | 969 | Light responsive element |
| *HvDJC33* | 147 | 153 | Light responsive element |
| *HvDJC33* | 296 | 290 | Light responsive element |
| *HvDJC33* | 452 | 446 | Light responsive element |
| *HvDJC33* | 1681 | 1687 | Light responsive element |
| *HvDJC33* | 1582 | 1572 | Light responsive element |
| *HvDJC33* | 851 | 845 | Light responsive element |
| *HvDJC33* | 1619 | 1613 | Light responsive element |
| *HvDJC33* | 883 | 889 | Low-temperature responsive element |
| *HvDJC33* | 695 | 701 | MYB |
| *HvDJC33* | 1406 | 1412 | MYB |
| *HvDJC33* | 1406 | 1412 | MYB |
| *HvDJC33* | 1178 | 1172 | MYC |
| *HvDJC33* | 1203 | 1209 | MYC |
| *HvDJC33* | 2002 | 2011 | Salicylic acid responsive element |
| *HvDJC33* | 100 | 109 | Zein metabolism regulation |
| *HvDJC33* | 1124 | 1115 | Zein metabolism regulation |
| *HvDJC34* | 1704 | 1699 | Abscisic acid responsive element |
| *HvDJC34* | 2005 | 2014 | Abscisic acid responsive element |
| *HvDJC34* | 443 | 437 | Anaerobic responsive element |
| *HvDJC34* | 891 | 885 | Anaerobic responsive element |
| *HvDJC34* | 1133 | 1127 | Anoxic responsive element |
| *HvDJC34* | 1837 | 1843 | Anoxic responsive element |
| *HvDJC34* | 659 | 668 | Defence responsive element |
| *HvDJC34* | 1728 | 1737 | Defence responsive element |
| *HvDJC34* | 1787 | 1778 | Defence responsive element |
| *HvDJC34* | 434 | 440 | Drought responsive element |
| *HvDJC34* | 439 | 433 | Light responsive element |
| *HvDJC34* | 452 | 458 | Light responsive element |
| *HvDJC34* | 1704 | 1710 | Light responsive element |
| *HvDJC34* | 1937 | 1943 | Light responsive element |
| *HvDJC34* | 302 | 296 | Light responsive element |
| *HvDJC34* | 573 | 567 | Light responsive element |
| *HvDJC34* | 663 | 669 | Light responsive element |
| *HvDJC34* | 1712 | 1718 | Low-temperature responsive element |
| *HvDJC34* | 431 | 426 | MeJA-responsive element |
| *HvDJC34* | 491 | 486 | MeJA-responsive element |
| *HvDJC34* | 1752 | 1747 | MeJA-responsive element |
| *HvDJC34* | 644 | 651 | MYB |
| *HvDJC34* | 41 | 47 | MYB |
| *HvDJC34* | 504 | 498 | MYB |
| *HvDJC34* | 918 | 912 | MYB |
| *HvDJC34* | 1742 | 1748 | MYB |
| *HvDJC34* | 434 | 440 | MYB |
| *HvDJC34* | 79 | 85 | MYB |
| *HvDJC34* | 918 | 912 | MYB |
| *HvDJC34* | 199 | 193 | MYC |
| *HvDJC34* | 240 | 249 | Zein metabolism regulation |
| *HvDJC34* | 1396 | 1405 | Zein metabolism regulation |
| *HvDJC35* | 34 | 29 | Abscisic acid responsive element |
| *HvDJC35* | 667 | 673 | Abscisic acid responsive element |
| *HvDJC35* | 668 | 673 | Abscisic acid responsive element |
| *HvDJC35* | 137 | 143 | Anoxic responsive element |
| *HvDJC35* | 1495 | 1489 | Anoxic responsive element |
| *HvDJC35* | 193 | 199 | Auxin-responsive element |
| *HvDJC35* | 479 | 473 | Auxin-responsive element |
| *HvDJC35* | 662 | 669 | Gibberellin-responsive element |
| *HvDJC35* | 892 | 898 | Light responsive element |
| *HvDJC35* | 1149 | 1142 | Light responsive element |
| *HvDJC35* | 91 | 82 | Light responsive element |
| *HvDJC35* | 235 | 229 | Light responsive element |
| *HvDJC35* | 667 | 673 | Light responsive element |
| *HvDJC35* | 1219 | 1225 | Light responsive element |
| *HvDJC35* | 34 | 40 | Light responsive element |
| *HvDJC35* | 667 | 673 | Light responsive element |
| *HvDJC35* | 1596 | 1602 | Light responsive element |
| *HvDJC35* | 1473 | 1467 | Light responsive element |
| *HvDJC35* | 1934 | 1928 | Light responsive element |
| *HvDJC35* | 21 | 28 | MYB |
| *HvDJC35* | 122 | 128 | MYB |
| *HvDJC35* | 846 | 852 | MYB |
| *HvDJC35* | 1102 | 1108 | MYB |
| *HvDJC35* | 1329 | 1335 | MYB |
| *HvDJC35* | 1353 | 1359 | MYB |
| *HvDJC35* | 122 | 128 | MYB |
| *HvDJC35* | 846 | 852 | MYB |
| *HvDJC35* | 1102 | 1108 | MYB |
| *HvDJC35* | 350 | 356 | MYC |
| *HvDJC35* | 1060 | 1066 | MYC |
| *HvDJC35* | 1433 | 1439 | MYC |
| *HvDJC35* | 1647 | 1641 | MYC |
| *HvDJC35* | 1683 | 1689 | MYC |
| *HvDJC35* | 1807 | 1801 | MYC |
| *HvDJC35* | 549 | 540 | Salicylic acid responsive element |
| *HvDJC35* | 873 | 864 | Zein metabolism regulation |
| *HvDJC36* | 256 | 261 | Abscisic acid responsive element |
| *HvDJC36* | 459 | 454 | Abscisic acid responsive element |
| *HvDJC36* | 712 | 707 | Abscisic acid responsive element |
| *HvDJC36* | 1024 | 1018 | Anaerobic responsive element |
| *HvDJC36* | 109 | 103 | Auxin-responsive element |
| *HvDJC36* | 1835 | 1844 | Circadian |
| *HvDJC36* | 274 | 282 | Light responsive element |
| *HvDJC36* | 1517 | 1510 | Light responsive element |
| *HvDJC36* | 255 | 249 | Light responsive element |
| *HvDJC36* | 348 | 342 | Light responsive element |
| *HvDJC36* | 459 | 465 | Light responsive element |
| *HvDJC36* | 1523 | 1529 | Light responsive element |
| *HvDJC36* | 1932 | 1938 | Light responsive element |
| *HvDJC36* | 712 | 718 | Light responsive element |
| *HvDJC36* | 1009 | 1018 | Light responsive element |
| *HvDJC36* | 756 | 750 | Low-temperature responsive element |
| *HvDJC36* | 1882 | 1876 | Low-temperature responsive element |
| *HvDJC36* | 162 | 157 | MeJA-responsive element |
| *HvDJC36* | 298 | 292 | MYB |
| *HvDJC36* | 302 | 296 | MYB |
| *HvDJC36* | 318 | 312 | MYB |
| *HvDJC36* | 615 | 609 | MYB |
| *HvDJC36* | 1559 | 1565 | MYB |
| *HvDJC36* | 615 | 609 | MYB |
| *HvDJC36* | 143 | 137 | MYC |
| *HvDJC36* | 611 | 617 | MYC |
| *HvDJC36* | 795 | 789 | MYC |
| *HvDJC36* | 851 | 857 | MYC |
| *HvDJC36* | 878 | 884 | MYC |
| *HvDJC36* | 1159 | 1165 | MYC |
| *HvDJC36* | 1242 | 1236 | MYC |
| *HvDJC36* | 148 | 157 | Zein metabolism regulation |
| *HvDJC36* | 323 | 332 | Zein metabolism regulation |
| *HvDJB05* | 180 | 175 | Abscisic acid responsive element |
| *HvDJB05* | 1152 | 1146 | Abscisic acid responsive element |
| *HvDJB05* | 1153 | 1158 | Abscisic acid responsive element |
| *HvDJB05* | 1501 | 1506 | Abscisic acid responsive element |
| *HvDJB05* | 1613 | 1608 | Abscisic acid responsive element |
| *HvDJB05* | 1698 | 1703 | Abscisic acid responsive element |
| *HvDJB05* | 180 | 174 | Abscisic acid responsive element |
| *HvDJB05* | 1500 | 1506 | Abscisic acid responsive element |
| *HvDJB05* | 180 | 186 | Abscisic acid responsive element |
| *HvDJB05* | 1500 | 1494 | Abscisic acid responsive element |
| *HvDJB05* | 248 | 242 | Anaerobic responsive element |
| *HvDJB05* | 725 | 719 | Anaerobic responsive element |
| *HvDJB05* | 1974 | 1980 | Anoxic responsive element |
| *HvDJB05* | 1602 | 1608 | Auxin-responsive element |
| *HvDJB05* | 622 | 613 | Cell cycle regulation |
| *HvDJB05* | 445 | 451 | Drought responsive element |
| *HvDJB05* | 1581 | 1587 | Drought responsive element |
| *HvDJB05* | 1644 | 1638 | Drought responsive element |
| *HvDJB05* | 884 | 877 | Gibberellin-responsive element |
| *HvDJB05* | 178 | 187 | Light responsive element |
| *HvDJB05* | 1470 | 1462 | Light responsive element |
| *HvDJB05* | 1506 | 1498 | Light responsive element |
| *HvDJB05* | 159 | 165 | Light responsive element |
| *HvDJB05* | 490 | 499.5 | Light responsive element |
| *HvDJB05* | 1152 | 1146 | Light responsive element |
| *HvDJB05* | 168 | 174 | Light responsive element |
| *HvDJB05* | 180 | 174 | Light responsive element |
| *HvDJB05* | 976 | 982 | Light responsive element |
| *HvDJB05* | 1150 | 1159 | Light responsive element |
| *HvDJB05* | 1152 | 1146 | Light responsive element |
| *HvDJB05* | 1500 | 1506 | Light responsive element |
| *HvDJB05* | 1613 | 1619 | Light responsive element |
| *HvDJB05* | 1697 | 1691 | Light responsive element |
| *HvDJB05* | 450 | 440 | Light responsive element |
| *HvDJB05* | 675 | 666 | Light responsive element |
| *HvDJB05* | 703 | 709 | Light responsive element |
| *HvDJB05* | 320 | 326 | Low-temperature responsive element |
| *HvDJB05* | 782 | 787 | MeJA-responsive element |
| *HvDJB05* | 1006 | 1011 | MeJA-responsive element |
| *HvDJB05* | 1338 | 1343 | MeJA-responsive element |
| *HvDJB05* | 1641 | 1636 | MeJA-responsive element |
| *HvDJB05* | 418 | 411 | MYB |
| *HvDJB05* | 445 | 451 | MYB |
| *HvDJB05* | 1581 | 1587 | MYB |
| *HvDJB05* | 1644 | 1638 | MYB |
| *HvDJB05* | 40 | 46 | MYB |
| *HvDJB05* | 884 | 890 | MYB |
| *HvDJB05* | 1247 | 1253 | MYB |
| *HvDJB05* | 1913 | 1919 | MYB |
| *HvDJB05* | 623 | 629 | MYB |
| *HvDJB05* | 1813 | 1807 | MYB |
| *HvDJB05* | 884 | 890 | MYB |
| *HvDJB05* | 1913 | 1919 | MYB |
| *HvDJB05* | 27 | 33 | MYC |
| *HvDJB05* | 452 | 446 | MYC |
| *HvDJB05* | 490 | 484 | MYC |
| *HvDJB05* | 1677 | 1671 | MYC |
| *HvDJB05* | 1752 | 1746 | MYC |
| *HvDJB05* | 915 | 906 | Salicylic acid responsive element |
| *HvDJB05* | 1412 | 1421 | Salicylic acid responsive element |
| *HvDJB05* | 983 | 992 | Zein metabolism regulation |
| *HvDJC37* | 1706 | 1711 | Abscisic acid responsive element |
| *HvDJC37* | 93 | 87 | Drought responsive element |
| *HvDJC37* | 354 | 361 | Gibberellin-responsive element |
| *HvDJC37* | 1705 | 1699 | Light responsive element |
| *HvDJC37* | 143 | 133 | Light responsive element |
| *HvDJC37* | 905 | 912 | Light responsive element |
| *HvDJC37* | 1625 | 1620 | MeJA-responsive element |
| *HvDJC37* | 294 | 301 | MYB |
| *HvDJC37* | 447 | 453 | MYB |
| *HvDJC37* | 497 | 491 | MYB |
| *HvDJC37* | 548 | 542 | MYB |
| *HvDJC37* | 1078 | 1072 | MYB |
| *HvDJC37* | 1659 | 1665 | MYB |
| *HvDJC37* | 1709 | 1703 | MYB |
| *HvDJC37* | 93 | 87 | MYB |
| *HvDJC37* | 590 | 584 | MYB |
| *HvDJC37* | 548 | 542 | MYB |
| *HvDJC37* | 447 | 453 | MYB |
| *HvDJC37* | 1659 | 1665 | MYB |
| *HvDJC37* | 38 | 32 | MYC |
| *HvDJC37* | 171 | 165 | MYC |
| *HvDJC37* | 173 | 179 | MYC |
| *HvDJC37* | 562 | 568 | MYC |
| *HvDJC37* | 1590 | 1584 | MYC |
| *HvDJC37* | 1596 | 1590 | MYC |
| *HvDJC37* | 1801 | 1807 | MYC |
| *HvDJC37* | 1850 | 1856 | MYC |
| *HvDJC37* | 1734 | 1726 | Seed-specific regulation |
| *HvDJC38* | 331 | 336 | Abscisic acid responsive element |
| *HvDJC38* | 410 | 417 | Abscisic acid responsive element |
| *HvDJC38* | 437 | 428 | Abscisic acid responsive element |
| *HvDJC38* | 439 | 445 | Abscisic acid responsive element |
| *HvDJC38* | 440 | 445 | Abscisic acid responsive element |
| *HvDJC38* | 1040 | 1045 | Abscisic acid responsive element |
| *HvDJC38* | 1570 | 1575 | Abscisic acid responsive element |
| *HvDJC38* | 321 | 327 | Anaerobic responsive element |
| *HvDJC38* | 398 | 404 | Anaerobic responsive element |
| *HvDJC38* | 419 | 425 | Anaerobic responsive element |
| *HvDJC38* | 1425 | 1431 | Anaerobic responsive element |
| *HvDJC38* | 868 | 874 | Anoxic responsive element |
| *HvDJC38* | 1886 | 1892 | Anoxic responsive element |
| *HvDJC38* | 1588 | 1597 | Circadian |
| *HvDJC38* | 1627 | 1621 | Drought responsive element |
| *HvDJC38* | 459 | 469.5 | Drought responsive element |
| *HvDJC38* | 575 | 567 | Light responsive element |
| *HvDJC38* | 439 | 445 | Light responsive element |
| *HvDJC38* | 474 | 480 | Light responsive element |
| *HvDJC38* | 330 | 324 | Light responsive element |
| *HvDJC38* | 439 | 445 | Light responsive element |
| *HvDJC38* | 1039 | 1033 | Light responsive element |
| *HvDJC38* | 1569 | 1563 | Light responsive element |
| *HvDJC38* | 1730 | 1720 | Light responsive element |
| *HvDJC38* | 247 | 253 | Low-temperature responsive element |
| *HvDJC38* | 486 | 491 | MeJA-responsive element |
| *HvDJC38* | 506 | 511 | MeJA-responsive element |
| *HvDJC38* | 975 | 980 | MeJA-responsive element |
| *HvDJC38* | 1996 | 2001 | MeJA-responsive element |
| *HvDJC38* | 537 | 543 | MYB |
| *HvDJC38* | 1596 | 1602 | MYB |
| *HvDJC38* | 1627 | 1621 | MYB |
| *HvDJC38* | 201 | 207 | MYB |
| *HvDJC38* | 201 | 207 | MYB |
| *HvDJC38* | 288 | 295 | MYC |
| *HvDJC38* | 1117 | 1123 | MYC |
| *HvDJC38* | 1258 | 1264 | MYC |
| *HvDJB06* | 477 | 472 | Abscisic acid responsive element |
| *HvDJB06* | 1491 | 1486 | Abscisic acid responsive element |
| *HvDJB06* | 1491 | 1485 | Abscisic acid responsive element |
| *HvDJB06* | 1491 | 1497 | Abscisic acid responsive element |
| *HvDJB06* | 1080 | 1074 | Anoxic responsive element |
| *HvDJB06* | 983 | 976 | Auxin-responsive element |
| *HvDJB06* | 160 | 169 | Circadian |
| *HvDJB06* | 164 | 155 | Circadian |
| *HvDJB06* | 200 | 209 | Circadian |
| *HvDJB06* | 204 | 195 | Circadian |
| *HvDJB06* | 1211 | 1202 | Defence responsive element |
| *HvDJB06* | 642 | 636 | Drought responsive element |
| *HvDJB06* | 1888 | 1894 | Drought responsive element |
| *HvDJB06* | 82 | 75 | Gibberellin-responsive element |
| *HvDJB06* | 1289 | 1283 | Light responsive element |
| *HvDJB06* | 24 | 14 | Light responsive element |
| *HvDJB06* | 477 | 483 | Light responsive element |
| *HvDJB06* | 1491 | 1485 | Light responsive element |
| *HvDJB06* | 440 | 434 | Light responsive element |
| *HvDJB06* | 589 | 583 | Light responsive element |
| *HvDJB06* | 1051 | 1045 | Light responsive element |
| *HvDJB06* | 197 | 192 | MeJA-responsive element |
| *HvDJB06* | 639 | 634 | MeJA-responsive element |
| *HvDJB06* | 716 | 721 | MeJA-responsive element |
| *HvDJB06* | 1369 | 1364 | MeJA-responsive element |
| *HvDJB06* | 642 | 636 | MYB |
| *HvDJB06* | 1888 | 1894 | MYB |
| *HvDJB06* | 415 | 421 | MYB |
| *HvDJB06* | 766 | 760 | MYB |
| *HvDJB06* | 885 | 879 | MYB |
| *HvDJB06* | 1752 | 1746 | MYB |
| *HvDJB06* | 245 | 251 | MYB |
| *HvDJB06* | 415 | 421 | MYB |
| *HvDJB06* | 27 | 33 | MYC |
| *HvDJB06* | 1305 | 1311 | MYC |
| *HvDJB06* | 1579 | 1573 | MYC |
| *HvDJB06* | 1690 | 1684 | MYC |
| *HvDJB06* | 1778 | 1772 | MYC |
| *HvDJB06* | 1858 | 1849 | Zein metabolism regulation |
| *HvDJC39* | 611 | 604 | Abscisic acid responsive element |
| *HvDJC39* | 847 | 837 | Abscisic acid responsive element |
| *HvDJC39* | 965 | 970 | Abscisic acid responsive element |
| *HvDJC39* | 1448 | 1443 | Abscisic acid responsive element |
| *HvDJC39* | 1554 | 1559 | Abscisic acid responsive element |
| *HvDJC39* | 100 | 106 | Anaerobic responsive element |
| *HvDJC39* | 334 | 328 | Anaerobic responsive element |
| *HvDJC39* | 524 | 530 | Anaerobic responsive element |
| *HvDJC39* | 1459 | 1453 | Anaerobic responsive element |
| *HvDJC39* | 283 | 289 | Anoxic responsive element |
| *HvDJC39* | 440 | 446 | Anoxic responsive element |
| *HvDJC39* | 119 | 128 | Circadian |
| *HvDJC39* | 600 | 606 | Drought responsive element |
| *HvDJC39* | 671 | 665 | Drought responsive element |
| *HvDJC39* | 1290 | 1297 | Gibberellin-responsive element |
| *HvDJC39* | 1150 | 1158 | Light responsive element |
| *HvDJC39* | 964 | 958 | Light responsive element |
| *HvDJC39* | 1446 | 1455 | Light responsive element |
| *HvDJC39* | 1553 | 1547 | Light responsive element |
| *HvDJC39* | 1448 | 1454 | Light responsive element |
| *HvDJC39* | 44 | 50 | Light responsive element |
| *HvDJC39* | 1411 | 1417 | Light responsive element |
| *HvDJC39* | 1724 | 1718 | Light responsive element |
| *HvDJC39* | 202 | 196 | Light responsive element |
| *HvDJC39* | 207 | 201 | Light responsive element |
| *HvDJC39* | 211 | 205 | Light responsive element |
| *HvDJC39* | 215 | 209 | Light responsive element |
| *HvDJC39* | 219 | 213 | Light responsive element |
| *HvDJC39* | 313 | 307 | Light responsive element |
| *HvDJC39* | 437 | 431 | Light responsive element |
| *HvDJC39* | 324 | 318 | Low-temperature responsive element |
| *HvDJC39* | 520 | 526 | Low-temperature responsive element |
| *HvDJC39* | 963 | 968 | MeJA-responsive element |
| *HvDJC39* | 1099 | 1094 | MeJA-responsive element |
| *HvDJC39* | 1453 | 1458 | MeJA-responsive element |
| *HvDJC39* | 1552 | 1557 | MeJA-responsive element |
| *HvDJC39* | 1747 | 1742 | MeJA-responsive element |
| *HvDJC39* | 2012 | 2017 | MeJA-responsive element |
| *HvDJC39* | 41 | 34 | MYB |
| *HvDJC39* | 1408 | 1401 | MYB |
| *HvDJC39* | 25 | 31 | MYB |
| *HvDJC39* | 86 | 80 | MYB |
| *HvDJC39* | 374 | 368 | MYB |
| *HvDJC39* | 1040 | 1034 | MYB |
| *HvDJC39* | 1120 | 1114 | MYB |
| *HvDJC39* | 1258 | 1252 | MYB |
| *HvDJC39* | 1690 | 1684 | MYB |
| *HvDJC39* | 1830 | 1824 | MYB |
| *HvDJC39* | 600 | 606 | MYB |
| *HvDJC39* | 671 | 665 | MYB |
| *HvDJC39* | 1005 | 999 | MYB |
| *HvDJC39* | 1021 | 1015 | MYB |
| *HvDJC39* | 1587 | 1581 | MYB |
| *HvDJC39* | 1721 | 1715 | MYB |
| *HvDJC39* | 979 | 985 | MYB |
| *HvDJC39* | 1826 | 1832 | MYB |
| *HvDJC39* | 25 | 31 | MYB |
| *HvDJC39* | 1040 | 1034 | MYB |
| *HvDJC39* | 620 | 626 | MYC |
| *HvDJC39* | 949 | 955 | MYC |
| *HvDJC39* | 1281 | 1287 | MYC |
| *HvDJC39* | 1491 | 1485 | MYC |
| *HvDJC39* | 1853 | 1859 | MYC |
| *HvDJC39* | 65 | 58 | MYC |
| *HvDJC40* | 1298 | 1307 | Abscisic acid responsive element |
| *HvDJC40* | 1497 | 1506 | Abscisic acid responsive element |
| *HvDJC40* | 1762 | 1752 | Abscisic acid responsive element |
| *HvDJC40* | 1792 | 1797 | Abscisic acid responsive element |
| *HvDJC40* | 1791 | 1797 | Abscisic acid responsive element |
| *HvDJC40* | 1791 | 1785 | Abscisic acid responsive element |
| *HvDJC40* | 921 | 915 | Anaerobic responsive element |
| *HvDJC40* | 1086 | 1080 | Anaerobic responsive element |
| *HvDJC40* | 1710 | 1704 | Anaerobic responsive element |
| *HvDJC40* | 825 | 819 | Anoxic responsive element |
| *HvDJC40* | 1723 | 1717 | Anoxic responsive element |
| *HvDJC40* | 403 | 409 | Auxin-responsive element |
| *HvDJC40* | 42 | 51 | Defence responsive element |
| *HvDJC40* | 798 | 805 | Endosperm |
| *HvDJC40* | 753 | 746 | Gibberellin-responsive element |
| *HvDJC40* | 301 | 294 | Gibberellin-responsive element |
| *HvDJC40* | 122 | 131 | Light responsive element |
| *HvDJC40* | 1639 | 1630 | Light responsive element |
| *HvDJC40* | 1073 | 1065 | Light responsive element |
| *HvDJC40* | 1909 | 1918 | Light responsive element |
| *HvDJC40* | 1914 | 1923 | Light responsive element |
| *HvDJC40* | 1161 | 1155 | Light responsive element |
| *HvDJC40* | 1463 | 1457 | Light responsive element |
| *HvDJC40* | 690 | 681 | Light responsive element |
| *HvDJC40* | 1662 | 1655 | Light responsive element |
| *HvDJC40* | 1790 | 1781 | Light responsive element |
| *HvDJC40* | 1791 | 1797 | Light responsive element |
| *HvDJC40* | 552 | 545 | Light responsive element |
| *HvDJC40* | 553 | 547 | Light responsive element |
| *HvDJC40* | 410 | 420 | Light responsive element |
| *HvDJC40* | 1405 | 1413 | Light responsive element |
| *HvDJC40* | 1783 | 1789 | Light responsive element |
| *HvDJC40* | 2011 | 2017 | Light responsive element |
| *HvDJC40* | 250 | 245 | MeJA-responsive element |
| *HvDJC40* | 312 | 306 | MYB |
| *HvDJC40* | 474 | 468 | MYB |
| *HvDJC40* | 554 | 560 | MYB |
| *HvDJC40* | 574 | 568 | MYB |
| *HvDJC40* | 753 | 759 | MYB |
| *HvDJC40* | 1622 | 1616 | MYB |
| *HvDJC40* | 1960 | 1966 | MYB |
| *HvDJC40* | 753 | 759 | MYB |
| *HvDJC40* | 474 | 468 | MYB |
| *HvDJC40* | 554 | 560 | MYB |
| *HvDJC40* | 416 | 410 | MYC |
| *HvDJC40* | 445 | 439 | MYC |
| *HvDJC40* | 1587 | 1581 | MYC |
| *HvDJC40* | 1589 | 1595 | MYC |
| *HvDJC41* | 387 | 382 | Abscisic acid responsive element |
| *HvDJC41* | 479 | 488 | Abscisic acid responsive element |
| *HvDJC41* | 784 | 775 | Abscisic acid responsive element |
| *HvDJC41* | 786 | 792 | Abscisic acid responsive element |
| *HvDJC41* | 787 | 792 | Abscisic acid responsive element |
| *HvDJC41* | 805 | 814 | Abscisic acid responsive element |
| *HvDJC41* | 807 | 802 | Abscisic acid responsive element |
| *HvDJC41* | 1243 | 1237 | Abscisic acid responsive element |
| *HvDJC41* | 1244 | 1249 | Abscisic acid responsive element |
| *HvDJC41* | 1940 | 1934 | Anoxic responsive element |
| *HvDJC41* | 1970 | 1976 | Anoxic responsive element |
| *HvDJC41* | 861 | 867 | Auxin-responsive element |
| *HvDJC41* | 876 | 882 | Auxin-responsive element |
| *HvDJC41* | 912 | 921 | Defence responsive element |
| *HvDJC41* | 170 | 176 | Drought responsive element |
| *HvDJC41* | 253 | 262 | Light responsive element |
| *HvDJC41* | 980 | 988 | Light responsive element |
| *HvDJC41* | 816 | 806 | Light responsive element |
| *HvDJC41* | 1996 | 2003 | Light responsive element |
| *HvDJC41* | 387 | 393 | Light responsive element |
| *HvDJC41* | 758 | 764 | Light responsive element |
| *HvDJC41* | 785 | 794 | Light responsive element |
| *HvDJC41* | 786 | 792 | Light responsive element |
| *HvDJC41* | 807 | 813 | Light responsive element |
| *HvDJC41* | 1243 | 1237 | Light responsive element |
| *HvDJC41* | 786 | 792 | Light responsive element |
| *HvDJC41* | 1243 | 1237 | Light responsive element |
| *HvDJC41* | 638 | 645 | Light responsive element |
| *HvDJC41* | 70 | 76 | Light responsive element |
| *HvDJC41* | 1717 | 1711 | Light responsive element |
| *HvDJC41* | 1800 | 1794 | Light responsive element |
| *HvDJC41* | 1805 | 1799 | Light responsive element |
| *HvDJC41* | 1810 | 1804 | Light responsive element |
| *HvDJC41* | 585 | 591 | Light responsive element |
| *HvDJC41* | 129 | 135 | Low-temperature responsive element |
| *HvDJC41* | 389 | 384 | MeJA-responsive element |
| *HvDJC41* | 266 | 260 | MYB |
| *HvDJC41* | 637 | 631 | MYB |
| *HvDJC41* | 700 | 706 | MYB |
| *HvDJC41* | 799 | 805 | MYB |
| *HvDJC41* | 1460 | 1466 | MYB |
| *HvDJC41* | 170 | 176 | MYB |
| *HvDJC41* | 373 | 367 | MYB |
| *HvDJC41* | 480 | 474 | MYB |
| *HvDJC41* | 1577 | 1571 | MYB |
| *HvDJC41* | 1612 | 1606 | MYB |
| *HvDJC41* | 266 | 260 | MYB |
| *HvDJC41* | 637 | 631 | MYB |
| *HvDJC41* | 700 | 706 | MYB |
| *HvDJC41* | 799 | 805 | MYB |
| *HvDJC41* | 1460 | 1466 | MYB |
| *HvDJC41* | 343 | 349 | MYC |
| *HvDJC41* | 392 | 386 | MYC |
| *HvDJC41* | 693 | 699 | MYC |
| *HvDJC41* | 866 | 860 | MYC |
| *HvDJC41* | 997 | 1003 | MYC |
| *HvDJC41* | 1373 | 1379 | MYC |
| *HvDJC41* | 676 | 666 | Zein metabolism regulation |
| *HvDJC42* | 575 | 580 | Abscisic acid responsive element |
| *HvDJC42* | 1329 | 1334 | Abscisic acid responsive element |
| *HvDJC42* | 574 | 580 | Abscisic acid responsive element |
| *HvDJC42* | 574 | 568 | Abscisic acid responsive element |
| *HvDJC42* | 1772 | 1766 | Anaerobic responsive element |
| *HvDJC42* | 1010 | 1016 | Anoxic responsive element |
| *HvDJC42* | 1963 | 1969 | Anoxic responsive element |
| *HvDJC42* | 270 | 264 | Auxin-responsive element |
| *HvDJC42* | 643 | 634 | Light responsive element |
| *HvDJC42* | 650 | 656 | Light responsive element |
| *HvDJC42* | 574 | 580 | Light responsive element |
| *HvDJC42* | 1328 | 1322 | Light responsive element |
| *HvDJC42* | 1202 | 1192 | Light responsive element |
| *HvDJC42* | 283 | 277 | Light responsive element |
| *HvDJC42* | 150 | 157 | Light responsive element |
| *HvDJC42* | 825 | 819 | Low-temperature responsive element |
| *HvDJC42* | 173 | 168 | MeJA-responsive element |
| *HvDJC42* | 402 | 397 | MeJA-responsive element |
| *HvDJC42* | 239 | 233 | MYB |
| *HvDJC42* | 819 | 813 | MYB |
| *HvDJC42* | 515 | 521 | MYC |
| *HvDJC42* | 963 | 957 | MYC |
| *HvDJC42* | 1843 | 1849 | MYC |
| *HvDJC43* | 1120 | 1115 | Abscisic acid responsive element |
| *HvDJC43* | 1640 | 1634 | Abscisic acid responsive element |
| *HvDJC43* | 1641 | 1646 | Abscisic acid responsive element |
| *HvDJC43* | 1296 | 1290 | Anaerobic responsive element |
| *HvDJC43* | 952 | 958 | Auxin-responsive element |
| *HvDJC43* | 1885 | 1894 | Circadian |
| *HvDJC43* | 1298 | 1307 | Defence responsive element |
| *HvDJC43* | 1785 | 1779 | Drought responsive element |
| *HvDJC43* | 1808 | 1802 | Drought responsive element |
| *HvDJC43* | 143 | 136 | Gibberellin-responsive element |
| *HvDJC43* | 1220 | 1212 | Light responsive element |
| *HvDJC43* | 1930 | 1938 | Light responsive element |
| *HvDJC43* | 201 | 207 | Light responsive element |
| *HvDJC43* | 522 | 528 | Light responsive element |
| *HvDJC43* | 1592 | 1600 | Light responsive element |
| *HvDJC43* | 1640 | 1634 | Light responsive element |
| *HvDJC43* | 1120 | 1126 | Light responsive element |
| *HvDJC43* | 1640 | 1634 | Light responsive element |
| *HvDJC43* | 382 | 372 | Light responsive element |
| *HvDJC43* | 1697 | 1687 | Light responsive element |
| *HvDJC43* | 933 | 927 | Light responsive element |
| *HvDJC43* | 1912 | 1918 | Light responsive element |
| *HvDJC43* | 329 | 324 | MeJA-responsive element |
| *HvDJC43* | 1091 | 1096 | MeJA-responsive element |
| *HvDJC43* | 1122 | 1117 | MeJA-responsive element |
| *HvDJC43* | 1785 | 1779 | MYB |
| *HvDJC43* | 1808 | 1802 | MYB |
| *HvDJC43* | 128 | 134 | MYB |
| *HvDJC43* | 1066 | 1060 | MYB |
| *HvDJC43* | 1155 | 1161 | MYB |
| *HvDJC43* | 1505 | 1511 | MYB |
| *HvDJC43* | 1805 | 1811 | MYB |
| *HvDJC43* | 1155 | 1161 | MYB |
| *HvDJC43* | 1805 | 1811 | MYB |
| *HvDJC43* | 35 | 41 | MYC |
| *HvDJC43* | 49 | 55 | MYC |
| *HvDJC43* | 88 | 94 | MYC |
| *HvDJC43* | 121 | 115 | MYC |
| *HvDJC43* | 377 | 383 | MYC |
| *HvDJC43* | 639 | 633 | MYC |
| *HvDJC43* | 664 | 670 | MYC |
| *HvDJC43* | 799 | 793 | MYC |
| *HvDJC43* | 979 | 973 | MYC |
| *HvDJC43* | 468 | 459 | Zein metabolism regulation |
| *HvDJC44* | 1080 | 1085 | Abscisic acid responsive element |
| *HvDJC44* | 982 | 988 | Anaerobic responsive element |
| *HvDJC44* | 1609 | 1603 | Anaerobic responsive element |
| *HvDJC44* | 734 | 728 | Auxin-responsive element |
| *HvDJC44* | 115 | 106 | Light responsive element |
| *HvDJC44* | 1164 | 1156 | Light responsive element |
| *HvDJC44* | 1079 | 1073 | Light responsive element |
| *HvDJC44* | 111 | 118 | Light responsive element |
| *HvDJC44* | 657 | 663 | Light responsive element |
| *HvDJC44* | 1484 | 1478 | Low-temperature responsive element |
| *HvDJC44* | 514 | 509 | MeJA-responsive element |
| *HvDJC44* | 1078 | 1083 | MeJA-responsive element |
| *HvDJC44* | 546 | 540 | MYB |
| *HvDJC44* | 787 | 781 | MYB |
| *HvDJC44* | 1510 | 1516 | MYB |
| *HvDJC44* | 787 | 781 | MYB |
| *HvDJC44* | 1673 | 1679 | MYC |
| *HvDJC44* | 828 | 837 | Salicylic acid responsive element |
| *HvDJC44* | 1076 | 1085 | Zein metabolism regulation |
| *HvDJC45* | 1845 | 1839 | Abscisic acid responsive element |
| *HvDJC45* | 1846 | 1851 | Abscisic acid responsive element |
| *HvDJC45* | 1192 | 1185 | Auxin-responsive element |
| *HvDJC45* | 419 | 428 | Defence responsive element |
| *HvDJC45* | 624 | 612 | Light responsive element |
| *HvDJC45* | 72 | 64 | Light responsive element |
| *HvDJC45* | 148 | 140 | Light responsive element |
| *HvDJC45* | 189 | 181 | Light responsive element |
| *HvDJC45* | 231 | 223 | Light responsive element |
| *HvDJC45* | 1660 | 1669 | Light responsive element |
| *HvDJC45* | 416 | 406 | Light responsive element |
| *HvDJC45* | 565 | 571 | Light responsive element |
| *HvDJC45* | 894 | 900 | Light responsive element |
| *HvDJC45* | 1744 | 1738 | Light responsive element |
| *HvDJC45* | 1845 | 1839 | Light responsive element |
| *HvDJC45* | 1845 | 1839 | Light responsive element |
| *HvDJC45* | 1491 | 1484 | Light responsive element |
| *HvDJC45* | 423 | 429 | Light responsive element |
| *HvDJC45* | 246 | 240 | Low-temperature responsive element |
| *HvDJC45* | 1825 | 1819 | Low-temperature responsive element |
| *HvDJC45* | 1383 | 1388 | MeJA-responsive element |
| *HvDJC45* | 391 | 397 | MYB |
| *HvDJC45* | 905 | 899 | MYB |
| *HvDJC45* | 706 | 712 | MYB |
| *HvDJC45* | 391 | 397 | MYB |
| *HvDJC45* | 482 | 488 | MYC |
| *HvDJC45* | 756 | 762 | MYC |
| *HvDJC45* | 1189 | 1183 | MYC |
| *HvDJC45* | 1595 | 1589 | MYC |
| *HvDJC45* | 1859 | 1868 | Salicylic acid responsive element |
| *HvDJC45* | 366 | 357 | Zein metabolism regulation |
| *HvDJC45* | 1622 | 1614 | Zein metabolism regulation |
| *HvDJC46* | 1579 | 1574 | Abscisic acid responsive element |
| *HvDJC46* | 1579 | 1573 | Abscisic acid responsive element |
| *HvDJC46* | 1579 | 1585 | Abscisic acid responsive element |
| *HvDJC46* | 1147 | 1153 | Auxin-responsive element |
| *HvDJC46* | 177 | 186 | Circadian |
| *HvDJC46* | 309 | 318 | Defence responsive element |
| *HvDJC46* | 233 | 245 | Gibberellin-responsive element |
| *HvDJC46* | 143 | 135 | Light responsive element |
| *HvDJC46* | 1579 | 1573 | Light responsive element |
| *HvDJC46* | 1600 | 1606 | Light responsive element |
| *HvDJC46* | 1975 | 1981 | Light responsive element |
| *HvDJC46* | 1885 | 1876 | Light responsive element |
| *HvDJC46* | 313 | 319 | Light responsive element |
| *HvDJC46* | 1569 | 1563 | Light responsive element |
| *HvDJC46* | 589 | 583 | Low-temperature responsive element |
| *HvDJC46* | 717 | 723 | Low-temperature responsive element |
| *HvDJC46* | 191 | 186 | MeJA-responsive element |
| *HvDJC46* | 1166 | 1161 | MeJA-responsive element |
| *HvDJC46* | 1241 | 1246 | MeJA-responsive element |
| *HvDJC46* | 1605 | 1600 | MeJA-responsive element |
| *HvDJC46* | 466 | 460 | MYB |
| *HvDJC46* | 422 | 416 | MYB |
| *HvDJC46* | 492 | 486 | MYB |
| *HvDJC46* | 652 | 658 | MYB |
| *HvDJC46* | 1174 | 1180 | MYB |
| *HvDJC46* | 1202 | 1208 | MYB |
| *HvDJC46* | 1372 | 1366 | MYB |
| *HvDJC46* | 1563 | 1569 | MYB |
| *HvDJC46* | 1984 | 1990 | MYB |
| *HvDJC46* | 885 | 891 | MYB |
| *HvDJC46* | 1174 | 1180 | MYB |
| *HvDJC46* | 492 | 486 | MYB |
| *HvDJC46* | 652 | 658 | MYB |
| *HvDJC46* | 1372 | 1366 | MYB |
| *HvDJC46* | 132 | 126 | MYC |
| *HvDJC46* | 440 | 434 | MYC |
| *HvDJC46* | 473 | 467 | MYC |
| *HvDJC46* | 574 | 568 | MYC |
| *HvDJC46* | 829 | 823 | MYC |
| *HvDJC47* | 985 | 991 | Anaerobic responsive element |
| *HvDJC47* | 376 | 370 | Anoxic responsive element |
| *HvDJC47* | 235 | 241 | Auxin-responsive element |
| *HvDJC47* | 1004 | 1010 | Auxin-responsive element |
| *HvDJC47* | 1272 | 1263 | Defence responsive element |
| *HvDJC47* | 1423 | 1414 | Defence responsive element |
| *HvDJC47* | 814 | 820 | Drought responsive element |
| *HvDJC47* | 899 | 905 | Light responsive element |
| *HvDJC47* | 1301 | 1295 | Light responsive element |
| *HvDJC47* | 1684 | 1677 | Light responsive element |
| *HvDJC47* | 683 | 671 | Light responsive element |
| *HvDJC47* | 1286 | 1292 | Light responsive element |
| *HvDJC47* | 782 | 791 | Light responsive element |
| *HvDJC47* | 1249 | 1236 | Light responsive element |
| *HvDJC47* | 553 | 546 | Light responsive element |
| *HvDJC47* | 93 | 99 | Light responsive element |
| *HvDJC47* | 861 | 867 | Light responsive element |
| *HvDJC47* | 645 | 650 | MeJA-responsive element |
| *HvDJC47* | 654 | 659 | MeJA-responsive element |
| *HvDJC47* | 756 | 749 | MYB |
| *HvDJC47* | 814 | 820 | MYB |
| *HvDJC47* | 731 | 725 | MYB |
| *HvDJC47* | 1487 | 1481 | MYB |
| *HvDJC47* | 1648 | 1642 | MYB |
| *HvDJC47* | 1793 | 1799 | MYB |
| *HvDJC47* | 1982 | 1976 | MYB |
| *HvDJC47* | 635 | 629 | MYB |
| *HvDJC47* | 641 | 635 | MYC |
| *HvDJC47* | 722 | 728 | MYC |
| *HvDJC47* | 1393 | 1387 | MYC |
| *HvDJC47* | 1745 | 1751 | MYC |
| *HvDJC48* | 613 | 608 | Abscisic acid responsive element |
| *HvDJC48* | 810 | 815 | Abscisic acid responsive element |
| *HvDJC48* | 824 | 831 | Abscisic acid responsive element |
| *HvDJC48* | 1610 | 1615 | Abscisic acid responsive element |
| *HvDJC48* | 1649 | 1655 | Anaerobic responsive element |
| *HvDJC48* | 367 | 361 | Anoxic responsive element |
| *HvDJC48* | 1857 | 1863 | Anoxic responsive element |
| *HvDJC48* | 221 | 214 | Gibberellin-responsive element |
| *HvDJC48* | 566 | 559 | Gibberellin-responsive element |
| *HvDJC48* | 1153 | 1161 | Light responsive element |
| *HvDJC48* | 1001 | 1011 | Light responsive element |
| *HvDJC48* | 613 | 619 | Light responsive element |
| *HvDJC48* | 489 | 483 | Light responsive element |
| *HvDJC48* | 809 | 803 | Light responsive element |
| *HvDJC48* | 1609 | 1603 | Light responsive element |
| *HvDJC48* | 1749 | 1755 | Low-temperature responsive element |
| *HvDJC48* | 808 | 813 | MeJA-responsive element |
| *HvDJC48* | 585 | 579 | MYB |
| *HvDJC48* | 53 | 59 | MYC |
| *HvDJC48* | 770 | 764 | MYC |
| *HvDJC48* | 1296 | 1290 | MYC |
| *HvDJC48* | 806 | 815 | Zein metabolism regulation |
| *HvDJC49* | 1060 | 1069 | Abscisic acid responsive element |
| *HvDJC49* | 1062 | 1056 | Abscisic acid responsive element |
| *HvDJC49* | 1063 | 1068 | Abscisic acid responsive element |
| *HvDJC49* | 1090 | 1080 | Abscisic acid responsive element |
| *HvDJC49* | 1092 | 1086 | Abscisic acid responsive element |
| *HvDJC49* | 1093 | 1098 | Abscisic acid responsive element |
| *HvDJC49* | 1610 | 1615 | Abscisic acid responsive element |
| *HvDJC49* | 1803 | 1797 | Abscisic acid responsive element |
| *HvDJC49* | 1804 | 1809 | Abscisic acid responsive element |
| *HvDJC49* | 1802 | 1794 | Abscisic acid responsive element |
| *HvDJC49* | 1609 | 1615 | Abscisic acid responsive element |
| *HvDJC49* | 1609 | 1603 | Abscisic acid responsive element |
| *HvDJC49* | 1332 | 1338 | Anoxic responsive element |
| *HvDJC49* | 475 | 469 | Auxin-responsive element |
| *HvDJC49* | 1560 | 1554 | Auxin-responsive element |
| *HvDJC49* | 1801 | 1792 | Light responsive element |
| *HvDJC49* | 1141 | 1132 | Light responsive element |
| *HvDJC49* | 136 | 127 | Light responsive element |
| *HvDJC49* | 1061 | 1070 | Light responsive element |
| *HvDJC49* | 1062 | 1056 | Light responsive element |
| *HvDJC49* | 1092 | 1086 | Light responsive element |
| *HvDJC49* | 1444 | 1438 | Light responsive element |
| *HvDJC49* | 1609 | 1615 | Light responsive element |
| *HvDJC49* | 1801 | 1811 | Light responsive element |
| *HvDJC49* | 1803 | 1797 | Light responsive element |
| *HvDJC49* | 1062 | 1056 | Light responsive element |
| *HvDJC49* | 1092 | 1086 | Light responsive element |
| *HvDJC49* | 1803 | 1797 | Light responsive element |
| *HvDJC49* | 1965 | 1959 | Light responsive element |
| *HvDJC49* | 1139 | 1130 | Light responsive element |
| *HvDJC49* | 1141 | 1150 | Light responsive element |
| *HvDJC49* | 846 | 854 | Light responsive element |
| *HvDJC49* | 878 | 886 | Light responsive element |
| *HvDJC49* | 1318 | 1312 | Light responsive element |
| *HvDJC49* | 963 | 956 | Light responsive element |
| *HvDJC49* | 1229 | 1222 | Light responsive element |
| *HvDJC49* | 277 | 283 | Light responsive element |
| *HvDJC49* | 1606 | 1612 | Light responsive element |
| *HvDJC49* | 565 | 560 | MeJA-responsive element |
| *HvDJC49* | 1589 | 1594 | MeJA-responsive element |
| *HvDJC49* | 1573 | 1567 | MYB |
| *HvDJC49* | 127 | 133 | MYB |
| *HvDJC49* | 1212 | 1218 | MYC |
| *HvDJC49* | 1439 | 1433 | MYC |
| *HvDJC49* | 431 | 421 | Zein metabolism regulation |
| *HvDJC50* | 889 | 894 | Abscisic acid responsive element |
| *HvDJC50* | 932 | 937 | Abscisic acid responsive element |
| *HvDJC50* | 962 | 967 | Abscisic acid responsive element |
| *HvDJC50* | 1728 | 1723 | Abscisic acid responsive element |
| *HvDJC50* | 888 | 894 | Abscisic acid responsive element |
| *HvDJC50* | 961 | 967 | Abscisic acid responsive element |
| *HvDJC50* | 1728 | 1722 | Abscisic acid responsive element |
| *HvDJC50* | 888 | 882 | Abscisic acid responsive element |
| *HvDJC50* | 961 | 955 | Abscisic acid responsive element |
| *HvDJC50* | 1728 | 1734 | Abscisic acid responsive element |
| *HvDJC50* | 78 | 72 | Anaerobic responsive element |
| *HvDJC50* | 331 | 337 | Anaerobic responsive element |
| *HvDJC50* | 1409 | 1415 | Anaerobic responsive element |
| *HvDJC50* | 1658 | 1652 | Anoxic responsive element |
| *HvDJC50* | 1674 | 1668 | Anoxic responsive element |
| *HvDJC50* | 1466 | 1455 | Auxin-responsive element |
| *HvDJC50* | 385 | 391 | Auxin-responsive element |
| *HvDJC50* | 153 | 144 | Cell cycle regulation |
| *HvDJC50* | 342 | 348 | Light responsive element |
| *HvDJC50* | 758 | 764 | Light responsive element |
| *HvDJC50* | 888 | 894 | Light responsive element |
| *HvDJC50* | 931 | 925 | Light responsive element |
| *HvDJC50* | 961 | 967 | Light responsive element |
| *HvDJC50* | 1725 | 1734 | Light responsive element |
| *HvDJC50* | 1728 | 1722 | Light responsive element |
| *HvDJC50* | 1188 | 1195 | Light responsive element |
| *HvDJC50* | 1671 | 1677 | Light responsive element |
| *HvDJC50* | 1777 | 1783 | Light responsive element |
| *HvDJC50* | 1895 | 1902 | Light responsive element |
| *HvDJC50* | 1809 | 1815 | Low-temperature responsive element |
| *HvDJC50* | 47 | 52 | MeJA-responsive element |
| *HvDJC50* | 930 | 935 | MeJA-responsive element |
| *HvDJC50* | 1709 | 1714 | MeJA-responsive element |
| *HvDJC50* | 1187 | 1181 | MYB |
| *HvDJC50* | 156 | 162 | MYB |
| *HvDJC50* | 1187 | 1181 | MYB |
| *HvDJC50* | 1142 | 1149 | MYC |
| *HvDJC50* | 769 | 775 | MYC |
| *HvDJC50* | 953 | 959 | MYC |
| *HvDJC50* | 958 | 967 | Zein metabolism regulation |
| *HvDJC51* | 202 | 212 | Abscisic acid responsive element |
| *HvDJC51* | 300 | 295 | Abscisic acid responsive element |
| *HvDJC51* | 1023 | 1018 | Abscisic acid responsive element |
| *HvDJC51* | 300 | 294 | Abscisic acid responsive element |
| *HvDJC51* | 300 | 306 | Abscisic acid responsive element |
| *HvDJC51* | 371 | 377 | Anaerobic responsive element |
| *HvDJC51* | 503 | 497 | Anaerobic responsive element |
| *HvDJC51* | 1415 | 1421 | Anoxic responsive element |
| *HvDJC51* | 1298 | 1304 | Auxin-responsive element |
| *HvDJC51* | 355 | 364 | Defence responsive element |
| *HvDJC51* | 1325 | 1319 | Drought responsive element |
| *HvDJC51* | 552 | 562.5 | Drought responsive element |
| *HvDJC51* | 1142 | 1149 | Endosperm |
| *HvDJC51* | 1861 | 1854 | Gibberellin-responsive element |
| *HvDJC51* | 1734 | 1741 | Gibberellin-responsive element |
| *HvDJC51* | 230 | 239 | Light responsive element |
| *HvDJC51* | 1545 | 1537 | Light responsive element |
| *HvDJC51* | 34 | 40 | Light responsive element |
| *HvDJC51* | 1790 | 1797 | Light responsive element |
| *HvDJC51* | 1023 | 1029 | Light responsive element |
| *HvDJC51* | 300 | 294 | Light responsive element |
| *HvDJC51* | 221 | 227 | Light responsive element |
| *HvDJC51* | 248 | 256 | Light responsive element |
| *HvDJC51* | 249 | 258 | Light responsive element |
| *HvDJC51* | 1450 | 1443 | Light responsive element |
| *HvDJC51* | 64 | 59 | MeJA-responsive element |
| *HvDJC51* | 1320 | 1315 | MeJA-responsive element |
| *HvDJC51* | 1462 | 1467 | MeJA-responsive element |
| *HvDJC51* | 1928 | 1923 | MeJA-responsive element |
| *HvDJC51* | 1083 | 1089 | MYB |
| *HvDJC51* | 1325 | 1319 | MYB |
| *HvDJC51* | 220 | 214 | MYB |
| *HvDJC51* | 558 | 552 | MYB |
| *HvDJC51* | 1056 | 1062 | MYB |
| *HvDJC51* | 838 | 844 | MYB |
| *HvDJC51* | 1184 | 1190 | MYB |
| *HvDJC51* | 1931 | 1925 | MYB |
| *HvDJC51* | 220 | 214 | MYB |
| *HvDJC51* | 558 | 552 | MYB |
| *HvDJC51* | 1056 | 1062 | MYB |
| *HvDJC51* | 1043 | 1049 | MYC |
| *HvDJC51* | 1309 | 1303 | MYC |
| *HvDJC51* | 1384 | 1378 | MYC |
| *HvDJC51* | 1407 | 1401 | MYC |
| *HvDJC51* | 1942 | 1936 | MYC |
| *HvDJC51* | 493 | 484 | Salicylic acid responsive element |
| *HvDJC51* | 1040 | 1049 | Zein metabolism regulation |
| *HvDJA01* | 1748 | 1743 | Abscisic acid responsive element |
| *HvDJA01* | 181 | 188 | Gibberellin-responsive element |
| *HvDJA01* | 848 | 858 | Light responsive element |
| *HvDJA01* | 490 | 499 | Light responsive element |
| *HvDJA01* | 324 | 332 | Light responsive element |
| *HvDJA01* | 310 | 317 | Light responsive element |
| *HvDJA01* | 1748 | 1754 | Light responsive element |
| *HvDJA01* | 1188 | 1194 | Light responsive element |
| *HvDJA01* | 1240 | 1233 | Light responsive element |
| *HvDJA01* | 1241 | 1235 | Light responsive element |
| *HvDJA01* | 105 | 112 | Light responsive element |
| *HvDJA01* | 440 | 434 | Light responsive element |
| *HvDJA01* | 1466 | 1472 | Light responsive element |
| *HvDJA01* | 1849 | 1843 | Low-temperature responsive element |
| *HvDJA01* | 1534 | 1539 | MeJA-responsive element |
| *HvDJA01* | 1611 | 1606 | MeJA-responsive element |
| *HvDJA01* | 1704 | 1699 | MeJA-responsive element |
| *HvDJA01* | 54 | 48 | MYB |
| *HvDJA01* | 84 | 78 | MYB |
| *HvDJA01* | 182 | 176 | MYB |
| *HvDJA01* | 636 | 642 | MYB |
| *HvDJA01* | 770 | 776 | MYB |
| *HvDJA01* | 805 | 799 | MYB |
| *HvDJA01* | 1242 | 1248 | MYB |
| *HvDJA01* | 1362 | 1368 | MYB |
| *HvDJA01* | 1614 | 1620 | MYB |
| *HvDJA01* | 1681 | 1687 | MYB |
| *HvDJA01* | 1734 | 1728 | MYB |
| *HvDJA01* | 1741 | 1735 | MYB |
| *HvDJA01* | 54 | 48 | MYB |
| *HvDJA01* | 182 | 176 | MYB |
| *HvDJA01* | 770 | 776 | MYB |
| *HvDJA01* | 805 | 799 | MYB |
| *HvDJA01* | 1741 | 1735 | MYB |
| *HvDJA01* | 84 | 78 | MYB |
| *HvDJA01* | 1242 | 1248 | MYB |
| *HvDJA01* | 976 | 982 | MYC |
| *HvDJA01* | 1310 | 1304 | MYC |
| *HvDJA01* | 954 | 963 | Zein metabolism regulation |
| *HvDJC52* | 429 | 424 | Abscisic acid responsive element |
| *HvDJC52* | 1027 | 1021 | Abscisic acid responsive element |
| *HvDJC52* | 1028 | 1033 | Abscisic acid responsive element |
| *HvDJC52* | 1878 | 1883 | Abscisic acid responsive element |
| *HvDJC52* | 1172 | 1178 | Anaerobic responsive element |
| *HvDJC52* | 1571 | 1565 | Anoxic responsive element |
| *HvDJC52* | 1805 | 1811 | Auxin-responsive element |
| *HvDJC52* | 252 | 243 | Defence responsive element |
| *HvDJC52* | 1166 | 1157 | Defence responsive element |
| *HvDJC52* | 317 | 325 | Light responsive element |
| *HvDJC52* | 1505 | 1496 | Light responsive element |
| *HvDJC52* | 534 | 540 | Light responsive element |
| *HvDJC52* | 981 | 990 | Light responsive element |
| *HvDJC52* | 429 | 435 | Light responsive element |
| *HvDJC52* | 1027 | 1021 | Light responsive element |
| *HvDJC52* | 1877 | 1871 | Light responsive element |
| *HvDJC52* | 1027 | 1021 | Light responsive element |
| *HvDJC52* | 1362 | 1372 | Light responsive element |
| *HvDJC52* | 1383 | 1377 | Light responsive element |
| *HvDJC52* | 1833 | 1839 | Light responsive element |
| *HvDJC52* | 1246 | 1239 | Light responsive element |
| *HvDJC52* | 45 | 39 | Light responsive element |
| *HvDJC52* | 786 | 780 | Light responsive element |
| *HvDJC52* | 1419 | 1413 | Low-temperature responsive element |
| *HvDJC52* | 1681 | 1675 | Low-temperature responsive element |
| *HvDJC52* | 1723 | 1729 | Low-temperature responsive element |
| *HvDJC52* | 1404 | 1399 | MeJA-responsive element |
| *HvDJC52* | 694 | 701 | MYB |
| *HvDJC52* | 1676 | 1669 | MYB |
| *HvDJC52* | 543 | 537 | MYB |
| *HvDJC52* | 740 | 746 | MYB |
| *HvDJC52* | 1327 | 1333 | MYB |
| *HvDJC52* | 1616 | 1610 | MYB |
| *HvDJC52* | 1690 | 1684 | MYB |
| *HvDJC52* | 1845 | 1839 | MYB |
| *HvDJC52* | 1291 | 1284 | MYC |
| *HvDJC52* | 369 | 375 | MYC |
| *HvDJC52* | 435 | 429 | MYC |
| *HvDJC52* | 744 | 750 | MYC |
| *HvDJC52* | 934 | 940 | MYC |
| *HvDJC52* | 1781 | 1775 | MYC |
| *HvDJC52* | 851 | 842 | Salicylic acid responsive element |
| *HvDJC53* | 750 | 755 | Abscisic acid responsive element |
| *HvDJC53* | 749 | 755 | Abscisic acid responsive element |
| *HvDJC53* | 749 | 743 | Abscisic acid responsive element |
| *HvDJC53* | 1030 | 1036 | Anoxic responsive element |
| *HvDJC53* | 1095 | 1101 | Anoxic responsive element |
| *HvDJC53* | 145 | 136 | Defence responsive element |
| *HvDJC53* | 1709 | 1703 | Drought responsive element |
| *HvDJC53* | 1870 | 1877 | Gibberellin-responsive element |
| *HvDJC53* | 1614 | 1608 | Light responsive element |
| *HvDJC53* | 1655 | 1649 | Light responsive element |
| *HvDJC53* | 530 | 537 | Light responsive element |
| *HvDJC53* | 249 | 255 | Light responsive element |
| *HvDJC53* | 749 | 755 | Light responsive element |
| *HvDJC53* | 948 | 955 | Light responsive element |
| *HvDJC53* | 699 | 704 | MeJA-responsive element |
| *HvDJC53* | 1620 | 1625 | MeJA-responsive element |
| *HvDJC53* | 2012 | 2017 | MeJA-responsive element |
| *HvDJC53* | 1305 | 1299 | MYB |
| *HvDJC53* | 1538 | 1532 | MYB |
| *HvDJC53* | 1680 | 1674 | MYB |
| *HvDJC53* | 1709 | 1703 | MYB |
| *HvDJC53* | 1945 | 1951 | MYB |
| *HvDJC53* | 1305 | 1299 | MYB |
| *HvDJC53* | 43 | 49 | MYC |
| *HvDJC53* | 258 | 252 | MYC |
| *HvDJC53* | 400 | 394 | MYC |
| *HvDJC53* | 689 | 695 | MYC |
| *HvDJC53* | 830 | 836 | MYC |
| *HvDJC53* | 1325 | 1319 | MYC |
| *HvDJC53* | 1861 | 1867 | MYC |
| *HvDJC54* | 1835 | 1828 | Abscisic acid responsive element |
| *HvDJC54* | 1278 | 1284 | Anaerobic responsive element |
| *HvDJC54* | 1865 | 1859 | Anaerobic responsive element |
| *HvDJC54* | 1589 | 1583 | Anoxic responsive element |
| *HvDJC54* | 1615 | 1621 | Anoxic responsive element |
| *HvDJC54* | 1670 | 1676 | Anoxic responsive element |
| *HvDJC54* | 1804 | 1798 | Anoxic responsive element |
| *HvDJC54* | 1937 | 1931 | Anoxic responsive element |
| *HvDJC54* | 1716 | 1710 | Auxin-responsive element |
| *HvDJC54* | 938 | 945 | Gibberellin-responsive element |
| *HvDJC54* | 1455 | 1462 | Gibberellin-responsive element |
| *HvDJC54* | 762 | 768 | Light responsive element |
| *HvDJC54* | 1157 | 1151 | Light responsive element |
| *HvDJC54* | 1234 | 1228 | Light responsive element |
| *HvDJC54* | 1489 | 1483 | Light responsive element |
| *HvDJC54* | 1532 | 1526 | Light responsive element |
| *HvDJC54* | 1552 | 1558 | Light responsive element |
| *HvDJC54* | 1612 | 1606 | Light responsive element |
| *HvDJC54* | 1807 | 1813 | Light responsive element |
| *HvDJC54* | 1908 | 1914 | Light responsive element |
| *HvDJC54* | 1689 | 1696 | Light responsive element |
| *HvDJC54* | 1353 | 1348 | MeJA-responsive element |
| *HvDJC54* | 1085 | 1091 | MYB |
| *HvDJC54* | 1105 | 1111 | MYB |
| *HvDJC54* | 1169 | 1175 | MYB |
| *HvDJC54* | 1410 | 1404 | MYB |
| *HvDJC54* | 812 | 806 | MYC |
| *HvDJC54* | 838 | 844 | MYC |
| *HvDJC55* | 1504 | 1499 | Abscisic acid responsive element |
| *HvDJC55* | 1646 | 1641 | Abscisic acid responsive element |
| *HvDJC55* | 1697 | 1706 | Abscisic acid responsive element |
| *HvDJC55* | 1646 | 1640 | Abscisic acid responsive element |
| *HvDJC55* | 1646 | 1652 | Abscisic acid responsive element |
| *HvDJC55* | 178 | 184 | Anaerobic responsive element |
| *HvDJC55* | 184 | 190 | Anaerobic responsive element |
| *HvDJC55* | 776 | 782 | Anaerobic responsive element |
| *HvDJC55* | 1028 | 1022 | Anoxic responsive element |
| *HvDJC55* | 1092 | 1086 | Anoxic responsive element |
| *HvDJC55* | 1126 | 1120 | Anoxic responsive element |
| *HvDJC55* | 1167 | 1161 | Anoxic responsive element |
| *HvDJC55* | 1328 | 1322 | Auxin-responsive element |
| *HvDJC55* | 1637 | 1646 | Circadian |
| *HvDJC55* | 1180 | 1186 | Drought responsive element |
| *HvDJC55* | 1353 | 1363.5 | Drought responsive element |
| *HvDJC55* | 1504 | 1510 | Light responsive element |
| *HvDJC55* | 1643 | 1652 | Light responsive element |
| *HvDJC55* | 1646 | 1640 | Light responsive element |
| *HvDJC55* | 968 | 974 | Light responsive element |
| *HvDJC55* | 989 | 995 | Light responsive element |
| *HvDJC55* | 1010 | 1016 | Light responsive element |
| *HvDJC55* | 1031 | 1037 | Light responsive element |
| *HvDJC55* | 1053 | 1059 | Light responsive element |
| *HvDJC55* | 1083 | 1089 | Light responsive element |
| *HvDJC55* | 1109 | 1115 | Light responsive element |
| *HvDJC55* | 1138 | 1144 | Light responsive element |
| *HvDJC55* | 1170 | 1176 | Light responsive element |
| *HvDJC55* | 509 | 516 | Light responsive element |
| *HvDJC55* | 1867 | 1873 | Light responsive element |
| *HvDJC55* | 633 | 638 | MeJA-responsive element |
| *HvDJC55* | 1506 | 1501 | MeJA-responsive element |
| *HvDJC55* | 160 | 166 | MYB |
| *HvDJC55* | 1180 | 1186 | MYB |
| *HvDJC55* | 1100 | 1094 | MYB |
| *HvDJC55* | 71 | 77 | MYC |
| *HvDJC55* | 665 | 659 | MYC |
| *HvDJC55* | 1612 | 1606 | MYC |
| *HvDJC56* | 33 | 27 | Anaerobic responsive element |
| *HvDJC56* | 1775 | 1781 | Anaerobic responsive element |
| *HvDJC56* | 449 | 443 | Auxin-responsive element |
| *HvDJC56* | 1533 | 1539 | Auxin-responsive element |
| *HvDJC56* | 1591 | 1585 | Auxin-responsive element |
| *HvDJC56* | 916 | 923 | Gibberellin-responsive element |
| *HvDJC56* | 1472 | 1464 | Light responsive element |
| *HvDJC56* | 434 | 440 | Light responsive element |
| *HvDJC56* | 797 | 803 | Light responsive element |
| *HvDJC56* | 1577 | 1571 | Light responsive element |
| *HvDJC56* | 897 | 907 | Light responsive element |
| *HvDJC56* | 637 | 643 | Light responsive element |
| *HvDJC56* | 1481 | 1475 | Light responsive element |
| *HvDJC56* | 1416 | 1410 | Light responsive element |
| *HvDJC56* | 550 | 545 | MeJA-responsive element |
| *HvDJC56* | 588 | 583 | MeJA-responsive element |
| *HvDJC56* | 1256 | 1261 | MeJA-responsive element |
| *HvDJC56* | 1338 | 1343 | MeJA-responsive element |
| *HvDJC56* | 286 | 280 | MYB |
| *HvDJC56* | 477 | 483 | MYB |
| *HvDJC56* | 1123 | 1129 | MYB |
| *HvDJC56* | 1180 | 1174 | MYB |
| *HvDJC56* | 214 | 208 | MYB |
| *HvDJC56* | 286 | 280 | MYB |
| *HvDJC56* | 1123 | 1129 | MYB |
| *HvDJC56* | 1180 | 1174 | MYB |
| *HvDJC56* | 782 | 788 | MYC |
| *HvDJC56* | 1174 | 1168 | MYC |
| *HvDJC56* | 1564 | 1558 | MYC |
| *HvDJC56* | 1133 | 1142 | Salicylic acid responsive element |
| *HvDJC56* | 1801 | 1792 | Salicylic acid responsive element |
| *HvDJA02* | 196 | 201 | Abscisic acid responsive element |
| *HvDJA02* | 252 | 245 | Abscisic acid responsive element |
| *HvDJA02* | 359 | 364 | Abscisic acid responsive element |
| *HvDJA02* | 468 | 463 | Abscisic acid responsive element |
| *HvDJA02* | 1088 | 1079 | Abscisic acid responsive element |
| *HvDJA02* | 1690 | 1695 | Abscisic acid responsive element |
| *HvDJA02* | 1613 | 1619 | Anoxic responsive element |
| *HvDJA02* | 94 | 101 | Auxin-responsive element |
| *HvDJA02* | 1333 | 1327 | Auxin-responsive element |
| *HvDJA02* | 243 | 237 | Drought responsive element |
| *HvDJA02* | 1388 | 1382 | Drought responsive element |
| *HvDJA02* | 1689 | 1683 | Light responsive element |
| *HvDJA02* | 195 | 189 | Light responsive element |
| *HvDJA02* | 358 | 352 | Light responsive element |
| *HvDJA02* | 468 | 474 | Light responsive element |
| *HvDJA02* | 1204 | 1210 | Light responsive element |
| *HvDJA02* | 1543 | 1537 | Light responsive element |
| *HvDJA02* | 2011 | 2005 | Light responsive element |
| *HvDJA02* | 1081 | 1088 | Light responsive element |
| *HvDJA02* | 1855 | 1849 | Low-temperature responsive element |
| *HvDJA02* | 1924 | 1918 | Low-temperature responsive element |
| *HvDJA02* | 1303 | 1298 | MeJA-responsive element |
| *HvDJA02* | 1561 | 1556 | MeJA-responsive element |
| *HvDJA02* | 1956 | 1961 | MeJA-responsive element |
| *HvDJA02* | 243 | 237 | MYB |
| *HvDJA02* | 872 | 878 | MYB |
| *HvDJA02* | 1367 | 1361 | MYB |
| *HvDJA02* | 1388 | 1382 | MYB |
| *HvDJA02* | 1454 | 1448 | MYB |
| *HvDJA02* | 512 | 506 | MYB |
| *HvDJA02* | 1711 | 1718 | MYC |
| *HvDJA02* | 1163 | 1157 | MYC |
| *HvDJA02* | 1425 | 1419 | MYC |
| *HvDJA03* | 468 | 475 | Abscisic acid responsive element |
| *HvDJA03* | 1527 | 1532 | Abscisic acid responsive element |
| *HvDJA03* | 382 | 388 | Anaerobic responsive element |
| *HvDJA03* | 1153 | 1147 | Anaerobic responsive element |
| *HvDJA03* | 1467 | 1460 | Auxin-responsive element |
| *HvDJA03* | 1142 | 1148 | Auxin-responsive element |
| *HvDJA03* | 1471 | 1462 | Cell cycle regulation |
| *HvDJA03* | 167 | 158 | Circadian |
| *HvDJA03* | 529 | 538 | Defence responsive element |
| *HvDJA03* | 338 | 332 | Drought responsive element |
| *HvDJA03* | 1838 | 1845 | Gibberellin-responsive element |
| *HvDJA03* | 1230 | 1237 | Light responsive element |
| *HvDJA03* | 1885 | 1894 | Light responsive element |
| *HvDJA03* | 240 | 234 | Light responsive element |
| *HvDJA03* | 1165 | 1171 | Light responsive element |
| *HvDJA03* | 1526 | 1520 | Light responsive element |
| *HvDJA03* | 1839 | 1849 | Light responsive element |
| *HvDJA03* | 128 | 122 | Light responsive element |
| *HvDJA03* | 912 | 906 | Light responsive element |
| *HvDJA03* | 1525 | 1530 | MeJA-responsive element |
| *HvDJA03* | 670 | 664 | MYB |
| *HvDJA03* | 1544 | 1538 | MYB |
| *HvDJA03* | 338 | 332 | MYB |
| *HvDJA03* | 367 | 361 | MYB |
| *HvDJA03* | 85 | 91 | MYB |
| *HvDJA03* | 1331 | 1325 | MYB |
| *HvDJA03* | 1472 | 1478 | MYB |
| *HvDJA03* | 1544 | 1538 | MYB |
| *HvDJA03* | 670 | 664 | MYB |
| *HvDJA03* | 141 | 147 | MYC |
| *HvDJA03* | 883 | 889 | MYC |
| *HvDJA03* | 1622 | 1616 | MYC |
| *HvDJA03* | 1748 | 1739 | Salicylic acid responsive element |
| *HvDJA03* | 246 | 256 | Zein metabolism regulation |
| *HvDJA03* | 785 | 794 | Zein metabolism regulation |
| *HvDJA03* | 1523 | 1532 | Zein metabolism regulation |
| *HvDJC57* | 828 | 834 | Abscisic acid responsive element |
| *HvDJC57* | 829 | 834 | Abscisic acid responsive element |
| *HvDJC57* | 1414 | 1419 | Abscisic acid responsive element |
| *HvDJC57* | 1413 | 1419 | Abscisic acid responsive element |
| *HvDJC57* | 1413 | 1407 | Abscisic acid responsive element |
| *HvDJC57* | 132 | 138 | Anaerobic responsive element |
| *HvDJC57* | 202 | 196 | Anaerobic responsive element |
| *HvDJC57* | 234 | 240 | Anaerobic responsive element |
| *HvDJC57* | 1797 | 1803 | Anaerobic responsive element |
| *HvDJC57* | 1814 | 1805 | Defence responsive element |
| *HvDJC57* | 1373 | 1366 | Endosperm |
| *HvDJC57* | 1922 | 1930 | Light responsive element |
| *HvDJC57* | 651 | 659 | Light responsive element |
| *HvDJC57* | 1807 | 1814 | Light responsive element |
| *HvDJC57* | 828 | 834 | Light responsive element |
| *HvDJC57* | 445 | 451 | Light responsive element |
| *HvDJC57* | 828 | 834 | Light responsive element |
| *HvDJC57* | 1413 | 1419 | Light responsive element |
| *HvDJC57* | 869 | 877 | Light responsive element |
| *HvDJC57* | 870 | 879 | Light responsive element |
| *HvDJC57* | 1994 | 1988 | Light responsive element |
| *HvDJC57* | 231 | 237 | Low-temperature responsive element |
| *HvDJC57* | 269 | 262 | MYB |
| *HvDJC57* | 1642 | 1648 | MYB |
| *HvDJC57* | 1708 | 1714 | MYB |
| *HvDJC57* | 1761 | 1755 | MYB |
| *HvDJC57* | 452 | 458 | MYB |
| *HvDJC57* | 539 | 533 | MYB |
| *HvDJC57* | 1642 | 1648 | MYB |
| *HvDJC57* | 163 | 169 | MYC |
| *HvDJC57* | 523 | 529 | MYC |
| *HvDJC57* | 1088 | 1094 | MYC |
| *HvDJC57* | 695 | 686 | Zein metabolism regulation |
| *HvDJC58* | 1197 | 1202 | Abscisic acid responsive element |
| *HvDJC58* | 1599 | 1589 | Abscisic acid responsive element |
| *HvDJC58* | 1601 | 1596 | Abscisic acid responsive element |
| *HvDJC58* | 1602 | 1592 | Abscisic acid responsive element |
| *HvDJC58* | 1704 | 1709 | Abscisic acid responsive element |
| *HvDJC58* | 1196 | 1202 | Abscisic acid responsive element |
| *HvDJC58* | 1601 | 1595 | Abscisic acid responsive element |
| *HvDJC58* | 1703 | 1709 | Abscisic acid responsive element |
| *HvDJC58* | 1196 | 1190 | Abscisic acid responsive element |
| *HvDJC58* | 1601 | 1607 | Abscisic acid responsive element |
| *HvDJC58* | 1703 | 1697 | Abscisic acid responsive element |
| *HvDJC58* | 1005 | 1011 | Auxin-responsive element |
| *HvDJC58* | 583 | 591.5 | Cell cycle regulation |
| *HvDJC58* | 1065 | 1073.5 | Cell cycle regulation |
| *HvDJC58* | 1119 | 1128 | Defence responsive element |
| *HvDJC58* | 890 | 883 | Gibberellin-responsive element |
| *HvDJC58* | 1194 | 1185 | Light responsive element |
| *HvDJC58* | 1292 | 1301 | Light responsive element |
| *HvDJC58* | 1506 | 1514 | Light responsive element |
| *HvDJC58* | 1550 | 1544 | Light responsive element |
| *HvDJC58* | 1133 | 1140 | Light responsive element |
| *HvDJC58* | 1196 | 1202 | Light responsive element |
| *HvDJC58* | 1601 | 1595 | Light responsive element |
| *HvDJC58* | 1703 | 1709 | Light responsive element |
| *HvDJC58* | 880 | 886 | Light responsive element |
| *HvDJC58* | 557 | 564 | Light responsive element |
| *HvDJC58* | 702 | 696 | Low-temperature responsive element |
| *HvDJC58* | 824 | 829 | MeJA-responsive element |
| *HvDJC58* | 855 | 860 | MeJA-responsive element |
| *HvDJC58* | 1940 | 1935 | MeJA-responsive element |
| *HvDJC58* | 696 | 702 | MYB |
| *HvDJC58* | 807 | 801 | MYB |
| *HvDJC58* | 1353 | 1359 | MYB |
| *HvDJC58* | 45 | 51 | MYC |
| *HvDJC58* | 1454 | 1448 | MYC |
| *HvDJC58* | 1760 | 1754 | MYC |
| *HvDJC58* | 524 | 515 | Salicylic acid responsive element |
| *HvDJC59* | 284 | 279 | Abscisic acid responsive element |
| *HvDJC59* | 1161 | 1152 | Abscisic acid responsive element |
| *HvDJC59* | 711 | 717 | Anaerobic responsive element |
| *HvDJC59* | 1218 | 1212 | Anoxic responsive element |
| *HvDJC59* | 548 | 557 | Defence responsive element |
| *HvDJC59* | 977 | 971 | Drought responsive element |
| *HvDJC59* | 495 | 502 | Light responsive element |
| *HvDJC59* | 512 | 522 | Light responsive element |
| *HvDJC59* | 284 | 290 | Light responsive element |
| *HvDJC59* | 284 | 276 | Light responsive element |
| *HvDJC59* | 1160 | 1149 | Light responsive element |
| *HvDJC59* | 1617 | 1611 | Light responsive element |
| *HvDJC59* | 51 | 45 | Low-temperature responsive element |
| *HvDJC59* | 378 | 383 | MeJA-responsive element |
| *HvDJC59* | 1513 | 1508 | MeJA-responsive element |
| *HvDJC59* | 676 | 682 | MYB |
| *HvDJC59* | 1089 | 1083 | MYB |
| *HvDJC59* | 1768 | 1774 | MYB |
| *HvDJC59* | 977 | 971 | MYB |
| *HvDJC59* | 1772 | 1766 | MYB |
| *HvDJC59* | 676 | 682 | MYB |
| *HvDJC59* | 79 | 73 | MYC |
| *HvDJC59* | 85 | 91 | MYC |
| *HvDJC59* | 124 | 130 | MYC |
| *HvDJC59* | 602 | 608 | MYC |
| *HvDJC59* | 770 | 764 | MYC |
| *HvDJC59* | 873 | 867 | MYC |
| *HvDJC59* | 1163 | 1169 | MYC |
| *HvDJC59* | 1170 | 1176 | MYC |
| *HvDJC59* | 1228 | 1234 | MYC |
| *HvDJC59* | 81 | 89 | Seed-specific regulation |
| *HvDJC60* | 1433 | 1439 | Auxin-responsive element |
| *HvDJC60* | 87 | 81 | Drought responsive element |
| *HvDJC60* | 1456 | 1463 | Gibberellin-responsive element |
| *HvDJC60* | 1203 | 1211 | Light responsive element |
| *HvDJC60* | 541 | 547 | Light responsive element |
| *HvDJC60* | 1615 | 1609 | Light responsive element |
| *HvDJC60* | 474 | 467 | Light responsive element |
| *HvDJC60* | 475 | 469 | Light responsive element |
| *HvDJC60* | 532 | 526 | Light responsive element |
| *HvDJC60* | 1965 | 1959 | Light responsive element |
| *HvDJC60* | 1144 | 1151 | Light responsive element |
| *HvDJC60* | 234 | 228 | Light responsive element |
| *HvDJC60* | 987 | 993 | Light responsive element |
| *HvDJC60* | 1369 | 1375 | Light responsive element |
| *HvDJC60* | 1375 | 1381 | Light responsive element |
| *HvDJC60* | 626 | 632 | Low-temperature responsive element |
| *HvDJC60* | 852 | 846 | Low-temperature responsive element |
| *HvDJC60* | 1260 | 1254 | Low-temperature responsive element |
| *HvDJC60* | 87 | 81 | MYB |
| *HvDJC60* | 326 | 332 | MYB |
| *HvDJC60* | 533 | 539 | MYB |
| *HvDJC60* | 1019 | 1025 | MYB |
| *HvDJC60* | 1413 | 1407 | MYB |
| *HvDJC60* | 1636 | 1630 | MYB |
| *HvDJC60* | 326 | 332 | MYB |
| *HvDJC60* | 533 | 539 | MYB |
| *HvDJC60* | 1019 | 1025 | MYB |
| *HvDJC60* | 101 | 107 | MYC |
| *HvDJC60* | 151 | 157 | MYC |
| *HvDJC60* | 790 | 784 | MYC |
| *HvDJC60* | 1269 | 1263 | MYC |
| *HvDJC60* | 1402 | 1408 | MYC |
| *HvDJC60* | 1524 | 1518 | MYC |
| *HvDJC60* | 352 | 360.5 | Palisade mesophyll cells |
| *HvDJC60* | 30 | 39 | Salicylic acid responsive element |
| *HvDJC60* | 147 | 155 | Seed-specific regulation |
| *HvDJC60* | 1062 | 1054 | Seed-specific regulation |
| *HvDJC61* | 999 | 1006 | Abscisic acid responsive element |
| *HvDJC61* | 1268 | 1274 | Anoxic responsive element |
| *HvDJC61* | 1303 | 1309 | Anoxic responsive element |
| *HvDJC61* | 1421 | 1427 | Anoxic responsive element |
| *HvDJC61* | 311 | 304 | Auxin-responsive element |
| *HvDJC61* | 1478 | 1472 | Auxin-responsive element |
| *HvDJC61* | 754 | 747 | Gibberellin-responsive element |
| *HvDJC61* | 1646 | 1638 | Light responsive element |
| *HvDJC61* | 1065 | 1058 | Light responsive element |
| *HvDJC61* | 1066 | 1060 | Light responsive element |
| *HvDJC61* | 187 | 197 | Light responsive element |
| *HvDJC61* | 1150 | 1144 | Light responsive element |
| *HvDJC61* | 1185 | 1179 | Light responsive element |
| *HvDJC61* | 1265 | 1259 | Light responsive element |
| *HvDJC61* | 1355 | 1349 | Light responsive element |
| *HvDJC61* | 1388 | 1382 | Light responsive element |
| *HvDJC61* | 1418 | 1412 | Light responsive element |
| *HvDJC61* | 1977 | 1983 | Light responsive element |
| *HvDJC61* | 152 | 147 | MeJA-responsive element |
| *HvDJC61* | 1450 | 1445 | MeJA-responsive element |
| *HvDJC61* | 268 | 261 | MYB |
| *HvDJC61* | 373 | 367 | MYB |
| *HvDJC61* | 1793 | 1787 | MYB |
| *HvDJC61* | 226 | 220 | MYB |
| *HvDJC61* | 236 | 242 | MYB |
| *HvDJC61* | 607 | 601 | MYB |
| *HvDJC61* | 754 | 760 | MYB |
| *HvDJC61* | 1067 | 1073 | MYB |
| *HvDJC61* | 754 | 760 | MYB |
| *HvDJC61* | 226 | 220 | MYB |
| *HvDJC61* | 1067 | 1073 | MYB |
| *HvDJC61* | 181 | 187 | MYC |
| *HvDJC61* | 571 | 577 | MYC |
| *HvDJC61* | 1903 | 1897 | MYC |
| *HvDJC61* | 1458 | 1467 | Salicylic acid responsive element |
| *HvDJC61* | 1915 | 1924 | Zein metabolism regulation |
| *HvDJC62* | 1283 | 1277 | Abscisic acid responsive element |
| *HvDJC62* | 1284 | 1289 | Abscisic acid responsive element |
| *HvDJC62* | 1475 | 1470 | Abscisic acid responsive element |
| *HvDJC62* | 613 | 607 | Anaerobic responsive element |
| *HvDJC62* | 1273 | 1267 | Anaerobic responsive element |
| *HvDJC62* | 1865 | 1859 | Anaerobic responsive element |
| *HvDJC62* | 1218 | 1209 | Defence responsive element |
| *HvDJC62* | 1535 | 1529 | Drought responsive element |
| *HvDJC62* | 646 | 638 | Light responsive element |
| *HvDJC62* | 1283 | 1277 | Light responsive element |
| *HvDJC62* | 1475 | 1481 | Light responsive element |
| *HvDJC62* | 1282 | 1274 | Light responsive element |
| *HvDJC62* | 1283 | 1277 | Light responsive element |
| *HvDJC62* | 632 | 627 | MeJA-responsive element |
| *HvDJC62* | 1033 | 1038 | MeJA-responsive element |
| *HvDJC62* | 1410 | 1405 | MeJA-responsive element |
| *HvDJC62* | 1609 | 1614 | MeJA-responsive element |
| *HvDJC62* | 1841 | 1836 | MeJA-responsive element |
| *HvDJC62* | 1943 | 1938 | MeJA-responsive element |
| *HvDJC62* | 1432 | 1438 | MYB |
| *HvDJC62* | 1302 | 1296 | MYB |
| *HvDJC62* | 1535 | 1529 | MYB |
| *HvDJC62* | 1006 | 1000 | MYB |
| *HvDJC62* | 1861 | 1867 | MYB |
| *HvDJC62* | 78 | 84 | MYC |
| *HvDJC62* | 500 | 494 | MYC |
| *HvDJC62* | 1018 | 1012 | MYC |
| *HvDJC62* | 1418 | 1424 | MYC |
| *HvDJC62* | 1768 | 1774 | MYC |
| *HvDJC62* | 1844 | 1838 | MYC |
| *HvDJC63* | 48 | 43 | Abscisic acid responsive element |
| *HvDJC63* | 1574 | 1579 | Abscisic acid responsive element |
| *HvDJC63* | 1719 | 1724 | Abscisic acid responsive element |
| *HvDJC63* | 441 | 435 | Anaerobic responsive element |
| *HvDJC63* | 1475 | 1481 | Anaerobic responsive element |
| *HvDJC63* | 310 | 316 | Auxin-responsive element |
| *HvDJC63* | 1722 | 1715 | Endosperm |
| *HvDJC63* | 1366 | 1359 | Gibberellin-responsive element |
| *HvDJC63* | 1282 | 1290 | Light responsive element |
| *HvDJC63* | 959 | 965 | Light responsive element |
| *HvDJC63* | 1410 | 1402 | Light responsive element |
| *HvDJC63* | 1573 | 1567 | Light responsive element |
| *HvDJC63* | 1718 | 1712 | Light responsive element |
| *HvDJC63* | 48 | 54 | Light responsive element |
| *HvDJC63* | 1696 | 1702 | Light responsive element |
| *HvDJC63* | 1890 | 1884 | Light responsive element |
| *HvDJC63* | 50 | 45 | MeJA-responsive element |
| *HvDJC63* | 148 | 143 | MeJA-responsive element |
| *HvDJC63* | 726 | 732 | MYC |
| *HvDJC63* | 999 | 1007 | Palisade mesophyll cells |
| *HvDJC63* | 1297 | 1288 | Salicylic acid responsive element |
| *HvDJC63* | 1325 | 1316 | Salicylic acid responsive element |
| *HvDJC63* | 1711 | 1702 | Zein metabolism regulation |
| *HvDJA04* | 464 | 469 | Abscisic acid responsive element |
| *HvDJA04* | 1084 | 1075 | Abscisic acid responsive element |
| *HvDJA04* | 1646 | 1637 | Abscisic acid responsive element |
| *HvDJA04* | 1788 | 1779 | Abscisic acid responsive element |
| *HvDJA04* | 1830 | 1825 | Abscisic acid responsive element |
| *HvDJA04* | 1830 | 1824 | Abscisic acid responsive element |
| *HvDJA04* | 1830 | 1836 | Abscisic acid responsive element |
| *HvDJA04* | 177 | 170 | Auxin-responsive element |
| *HvDJA04* | 1363 | 1357 | Auxin-responsive element |
| *HvDJA04* | 1275 | 1267 | Light responsive element |
| *HvDJA04* | 1344 | 1336 | Light responsive element |
| *HvDJA04* | 653 | 661 | Light responsive element |
| *HvDJA04* | 1487 | 1481 | Light responsive element |
| *HvDJA04* | 1499 | 1493 | Light responsive element |
| *HvDJA04* | 1513 | 1507 | Light responsive element |
| *HvDJA04* | 385 | 391 | Light responsive element |
| *HvDJA04* | 463 | 457 | Light responsive element |
| *HvDJA04* | 1830 | 1824 | Light responsive element |
| *HvDJA04* | 604 | 613 | Light responsive element |
| *HvDJA04* | 360 | 366 | Light responsive element |
| *HvDJA04* | 988 | 982 | Light responsive element |
| *HvDJA04* | 1127 | 1121 | Light responsive element |
| *HvDJA04* | 1613 | 1619 | Light responsive element |
| *HvDJA04* | 1942 | 1936 | Light responsive element |
| *HvDJA04* | 1882 | 1889 | Light responsive element |
| *HvDJA04* | 399 | 404 | MeJA-responsive element |
| *HvDJA04* | 456 | 461 | MeJA-responsive element |
| *HvDJA04* | 823 | 828 | MeJA-responsive element |
| *HvDJA04* | 1635 | 1630 | MeJA-responsive element |
| *HvDJA04* | 258 | 264 | MYB |
| *HvDJA04* | 718 | 712 | MYB |
| *HvDJA04* | 1104 | 1110 | MYB |
| *HvDJA04* | 348 | 354 | MYB |
| *HvDJA04* | 390 | 384 | MYB |
| *HvDJA04* | 804 | 810 | MYB |
| *HvDJA04* | 970 | 976 | MYB |
| *HvDJA04* | 1292 | 1298 | MYB |
| *HvDJA04* | 258 | 264 | MYB |
| *HvDJA04* | 718 | 712 | MYB |
| *HvDJA04* | 70 | 64 | MYC |
| *HvDJA04* | 1272 | 1266 | MYC |
| *HvDJA04* | 641 | 649 | Zein metabolism regulation |
| *HvDJC64* | 114 | 109 | Abscisic acid responsive element |
| *HvDJC64* | 1211 | 1206 | Abscisic acid responsive element |
| *HvDJC64* | 114 | 108 | Abscisic acid responsive element |
| *HvDJC64* | 114 | 120 | Abscisic acid responsive element |
| *HvDJC64* | 605 | 599 | Anaerobic responsive element |
| *HvDJC64* | 1356 | 1362 | Anaerobic responsive element |
| *HvDJC64* | 1429 | 1435 | Anaerobic responsive element |
| *HvDJC64* | 22 | 28 | Auxin-responsive element |
| *HvDJC64* | 973 | 979 | Auxin-responsive element |
| *HvDJC64* | 1436 | 1442 | Auxin-responsive element |
| *HvDJC64* | 777 | 771 | Drought responsive element |
| *HvDJC64* | 826 | 832 | Drought responsive element |
| *HvDJC64* | 1535 | 1542 | Gibberellin-responsive element |
| *HvDJC64* | 1079 | 1086 | Gibberellin-responsive element |
| *HvDJC64* | 1793 | 1800 | Gibberellin-responsive element |
| *HvDJC64* | 59 | 68 | Light responsive element |
| *HvDJC64* | 1871 | 1863 | Light responsive element |
| *HvDJC64* | 114 | 108 | Light responsive element |
| *HvDJC64* | 415 | 409 | Light responsive element |
| *HvDJC64* | 1211 | 1217 | Light responsive element |
| *HvDJC64* | 420 | 427 | Light responsive element |
| *HvDJC64* | 1124 | 1134 | Light responsive element |
| *HvDJC64* | 866 | 872 | Light responsive element |
| *HvDJC64* | 1961 | 1955 | Light responsive element |
| *HvDJC64* | 1965 | 1959 | Light responsive element |
| *HvDJC64* | 1855 | 1862 | Light responsive element |
| *HvDJC64* | 855 | 861 | Light responsive element |
| *HvDJC64* | 814 | 819 | MeJA-responsive element |
| *HvDJC64* | 913 | 918 | MeJA-responsive element |
| *HvDJC64* | 1118 | 1123 | MeJA-responsive element |
| *HvDJC64* | 1847 | 1852 | MeJA-responsive element |
| *HvDJC64* | 1891 | 1886 | MeJA-responsive element |
| *HvDJC64* | 125 | 132 | MYB |
| *HvDJC64* | 1815 | 1808 | MYB |
| *HvDJC64* | 777 | 771 | MYB |
| *HvDJC64* | 826 | 832 | MYB |
| *HvDJC64* | 29 | 23 | MYB |
| *HvDJC64* | 388 | 382 | MYB |
| *HvDJC64* | 419 | 413 | MYB |
| *HvDJC64* | 429 | 435 | MYB |
| *HvDJC64* | 1080 | 1074 | MYB |
| *HvDJC64* | 1164 | 1158 | MYB |
| *HvDJC64* | 1138 | 1144 | MYB |
| *HvDJC64* | 29 | 23 | MYB |
| *HvDJC64* | 429 | 435 | MYB |
| *HvDJC64* | 1080 | 1074 | MYB |
| *HvDJC64* | 1164 | 1158 | MYB |
| *HvDJC64* | 388 | 382 | MYB |
| *HvDJC64* | 419 | 413 | MYB |
| *HvDJC64* | 707 | 713 | MYC |
| *HvDJC64* | 1344 | 1338 | MYC |
| *HvDJC64* | 492 | 501 | Salicylic acid responsive element |
| *HvDJC64* | 1021 | 1012 | Zein metabolism regulation |
| *HvDJC65* | 825 | 830 | Abscisic acid responsive element |
| *HvDJC65* | 836 | 841 | Abscisic acid responsive element |
| *HvDJC65* | 909 | 914 | Abscisic acid responsive element |
| *HvDJC65* | 824 | 830 | Abscisic acid responsive element |
| *HvDJC65* | 824 | 818 | Abscisic acid responsive element |
| *HvDJC65* | 494 | 500 | Anaerobic responsive element |
| *HvDJC65* | 1618 | 1612 | Anaerobic responsive element |
| *HvDJC65* | 323 | 317 | Anoxic responsive element |
| *HvDJC65* | 938 | 947 | Defence responsive element |
| *HvDJC65* | 1432 | 1441 | Defence responsive element |
| *HvDJC65* | 1259 | 1252 | Gibberellin-responsive element |
| *HvDJC65* | 1055 | 1064 | Light responsive element |
| *HvDJC65* | 1534 | 1528 | Light responsive element |
| *HvDJC65* | 1586 | 1576 | Light responsive element |
| *HvDJC65* | 835 | 829 | Light responsive element |
| *HvDJC65* | 824 | 830 | Light responsive element |
| *HvDJC65* | 908 | 902 | Light responsive element |
| *HvDJC65* | 1070 | 1063 | Light responsive element |
| *HvDJC65* | 1071 | 1065 | Light responsive element |
| *HvDJC65* | 2011 | 2018 | Light responsive element |
| *HvDJC65* | 41 | 31 | Light responsive element |
| *HvDJC65* | 895 | 901 | Light responsive element |
| *HvDJC65* | 269 | 263 | Low-temperature responsive element |
| *HvDJC65* | 1551 | 1557 | Low-temperature responsive element |
| *HvDJC65* | 194 | 189 | MeJA-responsive element |
| *HvDJC65* | 298 | 303 | MeJA-responsive element |
| *HvDJC65* | 747 | 742 | MeJA-responsive element |
| *HvDJC65* | 148 | 142 | MYB |
| *HvDJC65* | 152 | 146 | MYB |
| *HvDJC65* | 1072 | 1078 | MYB |
| *HvDJC65* | 152 | 146 | MYB |
| *HvDJC65* | 1072 | 1078 | MYB |
| *HvDJC65* | 250 | 256 | MYC |
| *HvDJC65* | 1832 | 1824 | Zein metabolism regulation |
| *HvDJC66* | 1036 | 1042 | Anaerobic responsive element |
| *HvDJC66* | 1900 | 1906 | Anoxic responsive element |
| *HvDJC66* | 1935 | 1941 | Anoxic responsive element |
| *HvDJC66* | 1750 | 1759 | Circadian |
| *HvDJC66* | 859 | 848.5 | Drought responsive element |
| *HvDJC66* | 597 | 589 | Light responsive element |
| *HvDJC66* | 1778 | 1769 | Light responsive element |
| *HvDJC66* | 1903 | 1897 | Light responsive element |
| *HvDJC66* | 506 | 512 | Low-temperature responsive element |
| *HvDJC66* | 248 | 243 | MeJA-responsive element |
| *HvDJC66* | 760 | 765 | MeJA-responsive element |
| *HvDJC66* | 706 | 712 | MYB |
| *HvDJC66* | 1523 | 1529 | MYB |
| *HvDJC66* | 1766 | 1772 | MYB |
| *HvDJC66* | 1893 | 1899 | MYB |
| *HvDJC66* | 457 | 463 | MYB |
| *HvDJC66* | 517 | 523 | MYB |
| *HvDJC66* | 614 | 620 | MYB |
| *HvDJC66* | 1523 | 1529 | MYB |
| *HvDJC66* | 706 | 712 | MYB |
| *HvDJC66* | 594 | 600 | MYC |
| *HvDJC66* | 1130 | 1136 | MYC |
| *HvDJA05* | 363 | 358 | Abscisic acid responsive element |
| *HvDJA05* | 951 | 956 | Abscisic acid responsive element |
| *HvDJA05* | 1077 | 1070 | Abscisic acid responsive element |
| *HvDJA05* | 1152 | 1145 | Abscisic acid responsive element |
| *HvDJA05* | 363 | 357 | Abscisic acid responsive element |
| *HvDJA05* | 363 | 369 | Abscisic acid responsive element |
| *HvDJA05* | 286 | 295 | Anoxic responsive element |
| *HvDJA05* | 334 | 340 | Anoxic responsive element |
| *HvDJA05* | 1810 | 1816 | Anoxic responsive element |
| *HvDJA05* | 776 | 770 | Auxin-responsive element |
| *HvDJA05* | 1253 | 1263.5 | Drought responsive element |
| *HvDJA05* | 925 | 918 | Gibberellin-responsive element |
| *HvDJA05* | 694 | 700 | Light responsive element |
| *HvDJA05* | 363 | 357 | Light responsive element |
| *HvDJA05* | 915 | 921 | Light responsive element |
| *HvDJA05* | 950 | 944 | Light responsive element |
| *HvDJA05* | 1080 | 1086 | Light responsive element |
| *HvDJA05* | 1260 | 1266 | Light responsive element |
| *HvDJA05* | 1177 | 1186 | Light responsive element |
| *HvDJA05* | 1760 | 1754 | Light responsive element |
| *HvDJA05* | 1772 | 1766 | Light responsive element |
| *HvDJA05* | 1801 | 1795 | Light responsive element |
| *HvDJA05* | 1880 | 1874 | Light responsive element |
| *HvDJA05* | 97 | 103 | Light responsive element |
| *HvDJA05* | 816 | 822 | Light responsive element |
| *HvDJA05* | 1220 | 1214 | Low-temperature responsive element |
| *HvDJA05* | 949 | 954 | MeJA-responsive element |
| *HvDJA05* | 1670 | 1665 | MeJA-responsive element |
| *HvDJA05* | 131 | 125 | MYB |
| *HvDJA05* | 159 | 153 | MYB |
| *HvDJA05* | 680 | 674 | MYB |
| *HvDJA05* | 925 | 931 | MYB |
| *HvDJA05* | 1064 | 1058 | MYB |
| *HvDJA05* | 1678 | 1684 | MYB |
| *HvDJA05* | 1945 | 1951 | MYB |
| *HvDJA05* | 925 | 931 | MYB |
| *HvDJA05* | 373 | 379 | MYC |
| *HvDJA05* | 1186 | 1192 | MYC |
| *HvDJA05* | 1448 | 1440 | Seed-specific regulation |
| *HvDJC67* | 49 | 42 | Abscisic acid responsive element |
| *HvDJC67* | 558 | 553 | Abscisic acid responsive element |
| *HvDJC67* | 558 | 552 | Abscisic acid responsive element |
| *HvDJC67* | 558 | 564 | Abscisic acid responsive element |
| *HvDJC67* | 1264 | 1258 | Anaerobic responsive element |
| *HvDJC67* | 1332 | 1326 | Anaerobic responsive element |
| *HvDJC67* | 1627 | 1621 | Anaerobic responsive element |
| *HvDJC67* | 1853 | 1847 | Anaerobic responsive element |
| *HvDJC67* | 805 | 811 | Anoxic responsive element |
| *HvDJC67* | 854 | 860 | Anoxic responsive element |
| *HvDJC67* | 1289 | 1283 | Anoxic responsive element |
| *HvDJC67* | 1658 | 1665 | Gibberellin-responsive element |
| *HvDJC67* | 1705 | 1697 | Light responsive element |
| *HvDJC67* | 1736 | 1728 | Light responsive element |
| *HvDJC67* | 1408 | 1399 | Light responsive element |
| *HvDJC67* | 558 | 552 | Light responsive element |
| *HvDJC67* | 782 | 791 | Light responsive element |
| *HvDJC67* | 1734 | 1740 | Light responsive element |
| *HvDJC67* | 687 | 681 | Light responsive element |
| *HvDJC67* | 1004 | 998 | Light responsive element |
| *HvDJC67* | 950 | 957 | Light responsive element |
| *HvDJC67* | 1246 | 1240 | Low-temperature responsive element |
| *HvDJC67* | 477 | 482 | MeJA-responsive element |
| *HvDJC67* | 542 | 548 | MYB |
| *HvDJC67* | 1445 | 1439 | MYB |
| *HvDJC67* | 1504 | 1498 | MYB |
| *HvDJC67* | 1733 | 1727 | MYB |
| *HvDJC67* | 1533 | 1539 | MYB |
| *HvDJC67* | 1914 | 1920 | MYB |
| *HvDJC67* | 1733 | 1727 | MYB |
| *HvDJC67* | 278 | 284 | MYC |
| *HvDJC67* | 329 | 323 | MYC |
| *HvDJC67* | 1011 | 1005 | MYC |
| *HvDJC67* | 1698 | 1704 | MYC |
| *HvDJC67* | 1835 | 1841 | MYC |
| *HvDJC67* | 56 | 49 | MYC |
| *HvDJC67* | 1825 | 1816 | Salicylic acid responsive element |
| *HvDJC67* | 1128 | 1137 | Zein metabolism regulation |
| *HvDJC68* | 1721 | 1712 | Abscisic acid responsive element |
| *HvDJC68* | 1985 | 1990 | Abscisic acid responsive element |
| *HvDJC68* | 1993 | 1984 | Abscisic acid responsive element |
| *HvDJC68* | 471 | 464 | Auxin-responsive element |
| *HvDJC68* | 1247 | 1253 | Auxin-responsive element |
| *HvDJC68* | 517 | 525.5 | Cell cycle regulation |
| *HvDJC68* | 268 | 277 | Defence responsive element |
| *HvDJC68* | 219 | 212 | Gibberellin-responsive element |
| *HvDJC68* | 1683 | 1677 | Light responsive element |
| *HvDJC68* | 38 | 32 | Light responsive element |
| *HvDJC68* | 376 | 370 | Light responsive element |
| *HvDJC68* | 1433 | 1439 | Light responsive element |
| *HvDJC68* | 1624 | 1618 | Light responsive element |
| *HvDJC68* | 1984 | 1978 | Light responsive element |
| *HvDJC68* | 736 | 728 | Light responsive element |
| *HvDJC68* | 1532 | 1525 | Light responsive element |
| *HvDJC68* | 573 | 567 | Light responsive element |
| *HvDJC68* | 1734 | 1740 | Low-temperature responsive element |
| *HvDJC68* | 194 | 189 | MeJA-responsive element |
| *HvDJC68* | 855 | 850 | MeJA-responsive element |
| *HvDJC68* | 1031 | 1036 | MeJA-responsive element |
| *HvDJC68* | 1161 | 1156 | MeJA-responsive element |
| *HvDJC68* | 1184 | 1179 | MeJA-responsive element |
| *HvDJC68* | 80 | 86 | MYB |
| *HvDJC68* | 219 | 225 | MYB |
| *HvDJC68* | 584 | 578 | MYB |
| *HvDJC68* | 1151 | 1157 | MYB |
| *HvDJC68* | 1341 | 1347 | MYB |
| *HvDJC68* | 1492 | 1498 | MYB |
| *HvDJC68* | 519 | 513 | MYB |
| *HvDJC68* | 1579 | 1573 | MYB |
| *HvDJC68* | 1939 | 1933 | MYB |
| *HvDJC68* | 1945 | 1951 | MYB |
| *HvDJC68* | 80 | 86 | MYB |
| *HvDJC68* | 219 | 225 | MYB |
| *HvDJC68* | 242 | 248 | MYC |
| *HvDJC68* | 419 | 425 | MYC |
| *HvDJC68* | 1164 | 1158 | MYC |
| *HvDJC69* | 478 | 483 | Abscisic acid responsive element |
| *HvDJC69* | 852 | 847 | Abscisic acid responsive element |
| *HvDJC69* | 912 | 921 | Abscisic acid responsive element |
| *HvDJC69* | 1594 | 1599 | Abscisic acid responsive element |
| *HvDJC69* | 477 | 483 | Abscisic acid responsive element |
| *HvDJC69* | 477 | 471 | Abscisic acid responsive element |
| *HvDJC69* | 150 | 156 | Anoxic responsive element |
| *HvDJC69* | 367 | 373 | Drought responsive element |
| *HvDJC69* | 901 | 910 | Light responsive element |
| *HvDJC69* | 852 | 858 | Light responsive element |
| *HvDJC69* | 1593 | 1587 | Light responsive element |
| *HvDJC69* | 477 | 483 | Light responsive element |
| *HvDJC69* | 1188 | 1182 | Low-temperature responsive element |
| *HvDJC69* | 1559 | 1565 | Low-temperature responsive element |
| *HvDJC69* | 367 | 373 | MYB |
| *HvDJC69* | 1297 | 1303 | MYB |
| *HvDJC69* | 1569 | 1575 | MYB |
| *HvDJC69* | 70 | 64 | MYB |
| *HvDJC69* | 81 | 75 | MYB |
| *HvDJC69* | 167 | 173 | MYB |
| *HvDJC69* | 670 | 664 | MYB |
| *HvDJC69* | 1307 | 1301 | MYB |
| *HvDJC69* | 1538 | 1544 | MYB |
| *HvDJC69* | 1476 | 1482 | MYB |
| *HvDJC69* | 1945 | 1951 | MYB |
| *HvDJC69* | 70 | 64 | MYB |
| *HvDJC69* | 81 | 75 | MYB |
| *HvDJC69* | 167 | 173 | MYB |
| *HvDJC69* | 670 | 664 | MYB |
| *HvDJC69* | 1307 | 1301 | MYB |
| *HvDJC69* | 118 | 124 | MYC |
| *HvDJC69* | 591 | 597 | MYC |
| *HvDJC69* | 887 | 881 | MYC |
| *HvDJC69* | 1753 | 1747 | MYC |
| *HvDJC70* | 599 | 594 | Abscisic acid responsive element |
| *HvDJC70* | 806 | 812 | Abscisic acid responsive element |
| *HvDJC70* | 807 | 812 | Abscisic acid responsive element |
| *HvDJC70* | 971 | 977 | Abscisic acid responsive element |
| *HvDJC70* | 972 | 977 | Abscisic acid responsive element |
| *HvDJC70* | 2001 | 1996 | Abscisic acid responsive element |
| *HvDJC70* | 970 | 978 | Abscisic acid responsive element |
| *HvDJC70* | 704 | 698 | Anaerobic responsive element |
| *HvDJC70* | 1200 | 1206 | Anoxic responsive element |
| *HvDJC70* | 1221 | 1227 | Anoxic responsive element |
| *HvDJC70* | 1850 | 1841 | Circadian |
| *HvDJC70* | 316 | 308 | Light responsive element |
| *HvDJC70* | 1745 | 1739 | Light responsive element |
| *HvDJC70* | 1774 | 1768 | Light responsive element |
| *HvDJC70* | 686 | 679 | Light responsive element |
| *HvDJC70* | 806 | 812 | Light responsive element |
| *HvDJC70* | 969 | 978 | Light responsive element |
| *HvDJC70* | 971 | 977 | Light responsive element |
| *HvDJC70* | 1830 | 1824 | Light responsive element |
| *HvDJC70* | 2001 | 2007 | Light responsive element |
| *HvDJC70* | 599 | 605 | Light responsive element |
| *HvDJC70* | 806 | 812 | Light responsive element |
| *HvDJC70* | 971 | 977 | Light responsive element |
| *HvDJC70* | 523 | 512 | Light responsive element |
| *HvDJC70* | 1128 | 1122 | Light responsive element |
| *HvDJC70* | 1149 | 1143 | Light responsive element |
| *HvDJC70* | 1286 | 1280 | Light responsive element |
| *HvDJC70* | 1056 | 1063 | Light responsive element |
| *HvDJC70* | 299 | 293 | Light responsive element |
| *HvDJC70* | 325 | 331 | Low-temperature responsive element |
| *HvDJC70* | 593 | 599 | Low-temperature responsive element |
| *HvDJC70* | 1307 | 1312 | MeJA-responsive element |
| *HvDJC70* | 1813 | 1807 | MYB |
| *HvDJC70* | 952 | 946 | MYB |
| *HvDJC70* | 425 | 431 | MYC |
| *HvDJC70* | 764 | 758 | MYC |
| *HvDJC70* | 870 | 864 | MYC |
| *HvDJC70* | 905 | 911 | MYC |
| *HvDJC71* | 897 | 902 | Abscisic acid responsive element |
| *HvDJC71* | 2001 | 2010 | Abscisic acid responsive element |
| *HvDJC71* | 2004 | 2009 | Abscisic acid responsive element |
| *HvDJC71* | 896 | 902 | Abscisic acid responsive element |
| *HvDJC71* | 2003 | 2009 | Abscisic acid responsive element |
| *HvDJC71* | 896 | 890 | Abscisic acid responsive element |
| *HvDJC71* | 2003 | 1997 | Abscisic acid responsive element |
| *HvDJC71* | 668 | 662 | Anaerobic responsive element |
| *HvDJC71* | 262 | 256 | Auxin-responsive element |
| *HvDJC71* | 1903 | 1897 | Auxin-responsive element |
| *HvDJC71* | 292 | 283 | Circadian |
| *HvDJC71* | 227 | 236 | Defence responsive element |
| *HvDJC71* | 1421 | 1412 | Defence responsive element |
| *HvDJC71* | 77 | 83 | Drought responsive element |
| *HvDJC71* | 1782 | 1775 | Gibberellin-responsive element |
| *HvDJC71* | 1245 | 1254 | Light responsive element |
| *HvDJC71* | 1218 | 1210 | Light responsive element |
| *HvDJC71* | 896 | 902 | Light responsive element |
| *HvDJC71* | 2003 | 2009 | Light responsive element |
| *HvDJC71* | 65 | 59 | Low-temperature responsive element |
| *HvDJC71* | 835 | 829 | Low-temperature responsive element |
| *HvDJC71* | 953 | 947 | Low-temperature responsive element |
| *HvDJC71* | 1908 | 1902 | Low-temperature responsive element |
| *HvDJC71* | 1775 | 1770 | MeJA-responsive element |
| *HvDJC71* | 31 | 25 | MYB |
| *HvDJC71* | 1782 | 1788 | MYB |
| *HvDJC71* | 77 | 83 | MYB |
| *HvDJC71* | 783 | 777 | MYB |
| *HvDJC71* | 31 | 25 | MYB |
| *HvDJC71* | 1782 | 1788 | MYB |
| *HvDJC71* | 207 | 201 | MYC |
| *HvDJC71* | 1053 | 1059 | MYC |
| *HvDJC72* | 1945 | 1940 | Abscisic acid responsive element |
| *HvDJC72* | 986 | 980 | Anaerobic responsive element |
| *HvDJC72* | 119 | 125 | Anoxic responsive element |
| *HvDJC72* | 509 | 503 | Auxin-responsive element |
| *HvDJC72* | 1246 | 1254 | Light responsive element |
| *HvDJC72* | 1770 | 1764 | Light responsive element |
| *HvDJC72* | 1945 | 1951 | Light responsive element |
| *HvDJC72* | 526 | 520 | Light responsive element |
| *HvDJC72* | 1593 | 1587 | Light responsive element |
| *HvDJC72* | 1167 | 1173 | Low-temperature responsive element |
| *HvDJC72* | 144 | 139 | MeJA-responsive element |
| *HvDJC72* | 1528 | 1533 | MeJA-responsive element |
| *HvDJC72* | 769 | 776 | MYB |
| *HvDJC72* | 1650 | 1644 | MYC |
| *HvDJB07* | 757 | 752 | Abscisic acid responsive element |
| *HvDJB07* | 1985 | 1979 | Anaerobic responsive element |
| *HvDJB07* | 443 | 434 | Anoxic responsive element |
| *HvDJB07* | 835 | 829 | Anoxic responsive element |
| *HvDJB07* | 1723 | 1729 | Anoxic responsive element |
| *HvDJB07* | 1321 | 1328 | Gibberellin-responsive element |
| *HvDJB07* | 875 | 882 | Gibberellin-responsive element |
| *HvDJB07* | 1864 | 1857 | Gibberellin-responsive element |
| *HvDJB07* | 1965 | 1954 | Light responsive element |
| *HvDJB07* | 1225 | 1234 | Light responsive element |
| *HvDJB07* | 1231 | 1240 | Light responsive element |
| *HvDJB07* | 757 | 763 | Light responsive element |
| *HvDJB07* | 757 | 749 | Light responsive element |
| *HvDJB07* | 1226 | 1217 | Light responsive element |
| *HvDJB07* | 1227 | 1236 | Light responsive element |
| *HvDJB07* | 1602 | 1594 | Light responsive element |
| *HvDJB07* | 454 | 460 | Light responsive element |
| *HvDJB07* | 495 | 501 | Light responsive element |
| *HvDJB07* | 505 | 511 | Light responsive element |
| *HvDJB07* | 519 | 525 | Light responsive element |
| *HvDJB07* | 529 | 535 | Light responsive element |
| *HvDJB07* | 543 | 549 | Light responsive element |
| *HvDJB07* | 553 | 559 | Light responsive element |
| *HvDJB07* | 567 | 573 | Light responsive element |
| *HvDJB07* | 581 | 587 | Light responsive element |
| *HvDJB07* | 609 | 615 | Light responsive element |
| *HvDJB07* | 681 | 675 | Light responsive element |
| *HvDJB07* | 1473 | 1479 | Light responsive element |
| *HvDJB07* | 1481 | 1475 | Light responsive element |
| *HvDJB07* | 1350 | 1356 | Low-temperature responsive element |
| *HvDJB07* | 1561 | 1555 | Low-temperature responsive element |
| *HvDJB07* | 1759 | 1764 | MeJA-responsive element |
| *HvDJB07* | 1096 | 1103 | MYB |
| *HvDJB07* | 26 | 32 | MYB |
| *HvDJB07* | 933 | 927 | MYB |
| *HvDJB07* | 978 | 984 | MYB |
| *HvDJB07* | 1685 | 1679 | MYB |
| *HvDJB07* | 26 | 32 | MYB |
| *HvDJB07* | 1193 | 1187 | MYC |
| *HvDJC73* | 50 | 59 | Abscisic acid responsive element |
| *HvDJC73* | 52 | 58 | Abscisic acid responsive element |
| *HvDJC73* | 53 | 58 | Abscisic acid responsive element |
| *HvDJC73* | 694 | 699 | Abscisic acid responsive element |
| *HvDJC73* | 880 | 886 | Abscisic acid responsive element |
| *HvDJC73* | 881 | 886 | Abscisic acid responsive element |
| *HvDJC73* | 1810 | 1815 | Abscisic acid responsive element |
| *HvDJC73* | 1844 | 1850 | Anoxic responsive element |
| *HvDJC73* | 954 | 947 | Auxin-responsive element |
| *HvDJC73* | 1653 | 1660 | Auxin-responsive element |
| *HvDJC73* | 534 | 540 | Drought responsive element |
| *HvDJC73* | 1867 | 1859 | Light responsive element |
| *HvDJC73* | 1474 | 1483 | Light responsive element |
| *HvDJC73* | 677 | 683 | Light responsive element |
| *HvDJC73* | 129 | 122 | Light responsive element |
| *HvDJC73* | 29 | 23 | Light responsive element |
| *HvDJC73* | 47 | 35 | Light responsive element |
| *HvDJC73* | 49 | 60 | Light responsive element |
| *HvDJC73* | 50 | 59 | Light responsive element |
| *HvDJC73* | 52 | 58 | Light responsive element |
| *HvDJC73* | 880 | 886 | Light responsive element |
| *HvDJC73* | 887 | 881 | Light responsive element |
| *HvDJC73* | 1809 | 1803 | Light responsive element |
| *HvDJC73* | 52 | 58 | Light responsive element |
| *HvDJC73* | 693 | 687 | Light responsive element |
| *HvDJC73* | 880 | 886 | Light responsive element |
| *HvDJC73* | 305 | 300 | MeJA-responsive element |
| *HvDJC73* | 995 | 990 | MeJA-responsive element |
| *HvDJC73* | 1131 | 1126 | MeJA-responsive element |
| *HvDJC73* | 1197 | 1202 | MeJA-responsive element |
| *HvDJC73* | 1236 | 1242 | MYB |
| *HvDJC73* | 1520 | 1514 | MYB |
| *HvDJC73* | 1693 | 1687 | MYB |
| *HvDJC73* | 1731 | 1725 | MYB |
| *HvDJC73* | 534 | 540 | MYB |
| *HvDJC73* | 578 | 572 | MYB |
| *HvDJC73* | 832 | 826 | MYB |
| *HvDJC73* | 1236 | 1242 | MYB |
| *HvDJC73* | 1520 | 1514 | MYB |
| *HvDJC73* | 1693 | 1687 | MYB |
| *HvDJC73* | 231 | 237 | MYC |
| *HvDJC73* | 972 | 978 | MYC |
| *HvDJC73* | 1134 | 1140 | MYC |
| *HvDJC73* | 1378 | 1384 | MYC |
| *HvDJC73* | 1444 | 1438 | MYC |
| *HvDJC73* | 1090 | 1099 | Salicylic acid responsive element |
| *HvDJC73* | 162 | 153 | Zein metabolism regulation |
| *HvDJC74* | 982 | 987 | Abscisic acid responsive element |
| *HvDJC74* | 981 | 987 | Abscisic acid responsive element |
| *HvDJC74* | 981 | 975 | Abscisic acid responsive element |
| *HvDJC74* | 414 | 420 | Anaerobic responsive element |
| *HvDJC74* | 1605 | 1611 | Anaerobic responsive element |
| *HvDJC74* | 737 | 731 | Anoxic responsive element |
| *HvDJC74* | 1368 | 1374 | Anoxic responsive element |
| *HvDJC74* | 353 | 360 | Auxin-responsive element |
| *HvDJC74* | 75 | 81 | Drought responsive element |
| *HvDJC74* | 304 | 298 | Drought responsive element |
| *HvDJC74* | 1046 | 1040 | Drought responsive element |
| *HvDJC74* | 1426 | 1432 | Drought responsive element |
| *HvDJC74* | 1508 | 1502 | Drought responsive element |
| *HvDJC74* | 1979 | 1973 | Drought responsive element |
| *HvDJC74* | 748 | 755 | Endosperm |
| *HvDJC74* | 806 | 813 | Gibberellin-responsive element |
| *HvDJC74* | 1036 | 1029 | Gibberellin-responsive element |
| *HvDJC74* | 1672 | 1679 | Gibberellin-responsive element |
| *HvDJC74* | 1059 | 1053 | Light responsive element |
| *HvDJC74* | 981 | 987 | Light responsive element |
| *HvDJC74* | 1735 | 1728 | Light responsive element |
| *HvDJC74* | 1736 | 1730 | Light responsive element |
| *HvDJC74* | 125 | 116 | Light responsive element |
| *HvDJC74* | 62 | 56 | MYB |
| *HvDJC74* | 815 | 809 | MYB |
| *HvDJC74* | 987 | 981 | MYB |
| *HvDJC74* | 1036 | 1042 | MYB |
| *HvDJC74* | 1294 | 1288 | MYB |
| *HvDJC74* | 1447 | 1441 | MYB |
| *HvDJC74* | 1673 | 1667 | MYB |
| *HvDJC74* | 1737 | 1743 | MYB |
| *HvDJC74* | 1846 | 1840 | MYB |
| *HvDJC74* | 75 | 81 | MYB |
| *HvDJC74* | 238 | 244 | MYB |
| *HvDJC74* | 304 | 298 | MYB |
| *HvDJC74* | 1046 | 1040 | MYB |
| *HvDJC74* | 1426 | 1432 | MYB |
| *HvDJC74* | 1508 | 1502 | MYB |
| *HvDJC74* | 1773 | 1767 | MYB |
| *HvDJC74* | 1868 | 1874 | MYB |
| *HvDJC74* | 1979 | 1973 | MYB |
| *HvDJC74* | 987 | 981 | MYB |
| *HvDJC74* | 1036 | 1042 | MYB |
| *HvDJC74* | 1673 | 1667 | MYB |
| *HvDJC74* | 62 | 56 | MYB |
| *HvDJC74* | 815 | 809 | MYB |
| *HvDJC74* | 1737 | 1743 | MYB |
| *HvDJC74* | 1846 | 1840 | MYB |
| *HvDJC74* | 429 | 423 | MYC |
| *HvDJC74* | 914 | 920 | MYC |
| *HvDJC74* | 1704 | 1713 | Salicylic acid responsive element |
| *HvDJA06* | 74 | 69 | Abscisic acid responsive element |
| *HvDJA06* | 749 | 754 | Abscisic acid responsive element |
| *HvDJA06* | 796 | 801 | Abscisic acid responsive element |
| *HvDJA06* | 1250 | 1243 | Abscisic acid responsive element |
| *HvDJA06* | 1455 | 1460 | Abscisic acid responsive element |
| *HvDJA06* | 835 | 829 | Anaerobic responsive element |
| *HvDJA06* | 892 | 886 | Anaerobic responsive element |
| *HvDJA06* | 1038 | 1032 | Anaerobic responsive element |
| *HvDJA06* | 1402 | 1408 | Anaerobic responsive element |
| *HvDJA06* | 1419 | 1413 | Anaerobic responsive element |
| *HvDJA06* | 1621 | 1615 | Anaerobic responsive element |
| *HvDJA06* | 495 | 502 | Auxin-responsive element |
| *HvDJA06* | 1626 | 1634 | Light responsive element |
| *HvDJA06* | 579 | 586 | Light responsive element |
| *HvDJA06* | 795 | 789 | Light responsive element |
| *HvDJA06* | 74 | 80 | Light responsive element |
| *HvDJA06* | 748 | 742 | Light responsive element |
| *HvDJA06* | 989 | 995 | Light responsive element |
| *HvDJA06* | 1454 | 1448 | Light responsive element |
| *HvDJA06* | 58 | 64 | Light responsive element |
| *HvDJA06* | 485 | 475 | Light responsive element |
| *HvDJA06* | 945 | 935 | Light responsive element |
| *HvDJA06* | 547 | 540 | Light responsive element |
| *HvDJA06* | 574 | 567 | Light responsive element |
| *HvDJA06* | 635 | 642 | Light responsive element |
| *HvDJA06* | 1293 | 1300 | Light responsive element |
| *HvDJA06* | 76 | 71 | MeJA-responsive element |
| *HvDJA06* | 57 | 51 | MYB |
| *HvDJA06* | 395 | 401 | MYB |
| *HvDJA06* | 732 | 738 | MYB |
| *HvDJA06* | 1838 | 1832 | MYB |
| *HvDJA06* | 413 | 419 | MYB |
| *HvDJA06* | 450 | 456 | MYB |
| *HvDJA06* | 480 | 486 | MYB |
| *HvDJA06* | 1838 | 1832 | MYB |
| *HvDJA06* | 57 | 51 | MYB |
| *HvDJA06* | 732 | 738 | MYB |
| *HvDJA06* | 1883 | 1889 | MYC |
| *HvDJA06* | 678 | 687 | Zein metabolism regulation |
| *HvDJC75* | 1777 | 1772 | Abscisic acid responsive element |
| *HvDJC75* | 839 | 833 | Anaerobic responsive element |
| *HvDJC75* | 1117 | 1111 | Anoxic responsive element |
| *HvDJC75* | 1896 | 1902 | Anoxic responsive element |
| *HvDJC75* | 1994 | 1988 | Anoxic responsive element |
| *HvDJC75* | 976 | 970 | Auxin-responsive element |
| *HvDJC75* | 929 | 920.5 | Cell cycle regulation |
| *HvDJC75* | 1015 | 1009 | Drought responsive element |
| *HvDJC75* | 21 | 14 | Gibberellin-responsive element |
| *HvDJC75* | 823 | 831 | Light responsive element |
| *HvDJC75* | 48 | 54 | Light responsive element |
| *HvDJC75* | 1777 | 1783 | Light responsive element |
| *HvDJC75* | 1784 | 1778 | Light responsive element |
| *HvDJC75* | 1973 | 1979 | Light responsive element |
| *HvDJC75* | 1977 | 1983 | Light responsive element |
| *HvDJC75* | 1508 | 1514 | Low-temperature responsive element |
| *HvDJC75* | 1525 | 1519 | Low-temperature responsive element |
| *HvDJC75* | 1199 | 1204 | MeJA-responsive element |
| *HvDJC75* | 1779 | 1774 | MeJA-responsive element |
| *HvDJC75* | 21 | 27 | MYB |
| *HvDJC75* | 1109 | 1103 | MYB |
| *HvDJC75* | 103 | 109 | MYB |
| *HvDJC75* | 1015 | 1009 | MYB |
| *HvDJC75* | 21 | 27 | MYB |
| *HvDJC75* | 1109 | 1103 | MYB |
| *HvDJC75* | 1321 | 1315 | MYC |
| *HvDJC76* | 1073 | 1078 | Abscisic acid responsive element |
| *HvDJC76* | 1072 | 1078 | Abscisic acid responsive element |
| *HvDJC76* | 1072 | 1066 | Abscisic acid responsive element |
| *HvDJC76* | 1807 | 1813 | Anoxic responsive element |
| *HvDJC76* | 1975 | 1969 | Anoxic responsive element |
| *HvDJC76* | 802 | 796 | Auxin-responsive element |
| *HvDJC76* | 1441 | 1447 | Auxin-responsive element |
| *HvDJC76* | 779 | 773 | Drought responsive element |
| *HvDJC76* | 1306 | 1313 | Gibberellin-responsive element |
| *HvDJC76* | 508 | 495 | Light responsive element |
| *HvDJC76* | 542 | 548 | Light responsive element |
| *HvDJC76* | 1692 | 1685 | Light responsive element |
| *HvDJC76* | 1072 | 1078 | Light responsive element |
| *HvDJC76* | 1709 | 1716 | Light responsive element |
| *HvDJC76* | 1936 | 1930 | Light responsive element |
| *HvDJC76* | 550 | 544 | Low-temperature responsive element |
| *HvDJC76* | 1398 | 1404 | Low-temperature responsive element |
| *HvDJC76* | 311 | 306 | MeJA-responsive element |
| *HvDJC76* | 486 | 481 | MeJA-responsive element |
| *HvDJC76* | 1484 | 1479 | MeJA-responsive element |
| *HvDJC76* | 779 | 773 | MYB |
| *HvDJC76* | 705 | 699 | MYB |
| *HvDJC76* | 711 | 717 | MYB |
| *HvDJC76* | 1503 | 1497 | MYB |
| *HvDJC76* | 1553 | 1547 | MYB |
| *HvDJC76* | 466 | 460 | MYC |
| *HvDJC76* | 475 | 469 | MYC |
| *HvDJC76* | 344 | 353 | Zein metabolism regulation |
| *HvDJC77* | 977 | 972 | Abscisic acid responsive element |
| *HvDJC77* | 1488 | 1493 | Abscisic acid responsive element |
| *HvDJC77* | 1779 | 1774 | Abscisic acid responsive element |
| *HvDJC77* | 977 | 971 | Abscisic acid responsive element |
| *HvDJC77* | 977 | 983 | Abscisic acid responsive element |
| *HvDJC77* | 65 | 71 | Anaerobic responsive element |
| *HvDJC77* | 1437 | 1431 | Anoxic responsive element |
| *HvDJC77* | 38 | 45 | Auxin-responsive element |
| *HvDJC77* | 1280 | 1286 | Auxin-responsive element |
| *HvDJC77* | 1677 | 1683 | Auxin-responsive element |
| *HvDJC77* | 1900 | 1906 | Auxin-responsive element |
| *HvDJC77* | 800 | 794 | Drought responsive element |
| *HvDJC77* | 714 | 724 | Light responsive element |
| *HvDJC77* | 310 | 316 | Light responsive element |
| *HvDJC77* | 1608 | 1600 | Light responsive element |
| *HvDJC77* | 977 | 971 | Light responsive element |
| *HvDJC77* | 1779 | 1785 | Light responsive element |
| *HvDJC77* | 1487 | 1481 | Light responsive element |
| *HvDJC77* | 1730 | 1740 | Light responsive element |
| *HvDJC77* | 960 | 953 | Light responsive element |
| *HvDJC77* | 961 | 955 | Light responsive element |
| *HvDJC77* | 1409 | 1415 | Light responsive element |
| *HvDJC77* | 1353 | 1358 | MeJA-responsive element |
| *HvDJC77* | 800 | 794 | MYB |
| *HvDJC77* | 742 | 748 | MYB |
| *HvDJC77* | 742 | 748 | MYB |
| *HvDJC77* | 620 | 627 | MYC |
| *HvDJC77* | 636 | 642 | MYC |
| *HvDJC77* | 1710 | 1704 | MYC |
| *HvDJC77* | 1732 | 1726 | MYC |
| *HvDJC77* | 749 | 757 | Seed-specific regulation |
| *HvDJC78* | 1407 | 1401 | Abscisic acid responsive element |
| *HvDJC78* | 1408 | 1413 | Abscisic acid responsive element |
| *HvDJC78* | 1736 | 1730 | Anaerobic responsive element |
| *HvDJC78* | 1983 | 1989 | Anoxic responsive element |
| *HvDJC78* | 1681 | 1674 | Auxin-responsive element |
| *HvDJC78* | 167 | 161 | Drought responsive element |
| *HvDJC78* | 417 | 411 | Drought responsive element |
| *HvDJC78* | 667 | 661 | Drought responsive element |
| *HvDJC78* | 917 | 911 | Drought responsive element |
| *HvDJC78* | 1167 | 1161 | Drought responsive element |
| *HvDJC78* | 351 | 358 | Gibberellin-responsive element |
| *HvDJC78* | 601 | 608 | Gibberellin-responsive element |
| *HvDJC78* | 851 | 858 | Gibberellin-responsive element |
| *HvDJC78* | 1769 | 1776 | Gibberellin-responsive element |
| *HvDJC78* | 55 | 61 | Light responsive element |
| *HvDJC78* | 83 | 89 | Light responsive element |
| *HvDJC78* | 147 | 153 | Light responsive element |
| *HvDJC78* | 305 | 311 | Light responsive element |
| *HvDJC78* | 333 | 339 | Light responsive element |
| *HvDJC78* | 397 | 403 | Light responsive element |
| *HvDJC78* | 555 | 561 | Light responsive element |
| *HvDJC78* | 583 | 589 | Light responsive element |
| *HvDJC78* | 647 | 653 | Light responsive element |
| *HvDJC78* | 805 | 811 | Light responsive element |
| *HvDJC78* | 833 | 839 | Light responsive element |
| *HvDJC78* | 897 | 903 | Light responsive element |
| *HvDJC78* | 1055 | 1049 | Light responsive element |
| *HvDJC78* | 1083 | 1077 | Light responsive element |
| *HvDJC78* | 1147 | 1141 | Light responsive element |
| *HvDJC78* | 1305 | 1299 | Light responsive element |
| *HvDJC78* | 1332 | 1326 | Light responsive element |
| *HvDJC78* | 1407 | 1401 | Light responsive element |
| *HvDJC78* | 1406 | 1398 | Light responsive element |
| *HvDJC78* | 1407 | 1401 | Light responsive element |
| *HvDJC78* | 46 | 40 | Low-temperature responsive element |
| *HvDJC78* | 296 | 290 | Low-temperature responsive element |
| *HvDJC78* | 546 | 540 | Low-temperature responsive element |
| *HvDJC78* | 796 | 790 | Low-temperature responsive element |
| *HvDJC78* | 1046 | 1040 | Low-temperature responsive element |
| *HvDJC78* | 1384 | 1379 | MeJA-responsive element |
| *HvDJC78* | 1422 | 1417 | MeJA-responsive element |
| *HvDJC78* | 1546 | 1551 | MeJA-responsive element |
| *HvDJC78* | 167 | 161 | MYB |
| *HvDJC78* | 417 | 411 | MYB |
| *HvDJC78* | 667 | 661 | MYB |
| *HvDJC78* | 917 | 911 | MYB |
| *HvDJC78* | 1167 | 1161 | MYB |
| *HvDJC78* | 209 | 215 | MYB |
| *HvDJC78* | 459 | 465 | MYB |
| *HvDJC78* | 709 | 715 | MYB |
| *HvDJC78* | 959 | 965 | MYB |
| *HvDJC78* | 1209 | 1215 | MYB |
| *HvDJC78* | 1560 | 1554 | MYC |
| *HvDJC78* | 614 | 622 | Seed-specific regulation |
| *HvDJC78* | 864 | 872 | Seed-specific regulation |
| *HvDJC78* | 1114 | 1106 | Seed-specific regulation |
| *HvDJA07* | 237 | 242 | Abscisic acid responsive element |
| *HvDJA07* | 1963 | 1954 | Abscisic acid responsive element |
| *HvDJA07* | 236 | 242 | Abscisic acid responsive element |
| *HvDJA07* | 236 | 230 | Abscisic acid responsive element |
| *HvDJA07* | 1958 | 1964 | Anoxic responsive element |
| *HvDJA07* | 841 | 847 | Auxin-responsive element |
| *HvDJA07* | 115 | 124 | Circadian |
| *HvDJA07* | 1055 | 1046 | Defence responsive element |
| *HvDJA07* | 1417 | 1408 | Defence responsive element |
| *HvDJA07* | 1908 | 1914 | Drought responsive element |
| *HvDJA07* | 907 | 898 | Light responsive element |
| *HvDJA07* | 1469 | 1477 | Light responsive element |
| *HvDJA07* | 234 | 225 | Light responsive element |
| *HvDJA07* | 236 | 242 | Light responsive element |
| *HvDJA07* | 1922 | 1928 | Light responsive element |
| *HvDJA07* | 1961 | 1955 | Light responsive element |
| *HvDJA07* | 125 | 119 | Low-temperature responsive element |
| *HvDJA07* | 1857 | 1862 | MeJA-responsive element |
| *HvDJA07* | 1143 | 1150 | MYB |
| *HvDJA07* | 670 | 676 | MYB |
| *HvDJA07* | 778 | 784 | MYB |
| *HvDJA07* | 1287 | 1281 | MYB |
| *HvDJA07* | 1155 | 1149 | MYB |
| *HvDJA07* | 1908 | 1914 | MYB |
| *HvDJA07* | 1287 | 1281 | MYB |
| *HvDJA07* | 778 | 784 | MYB |
| *HvDJA07* | 226 | 220 | MYC |
| *HvDJA07* | 627 | 621 | MYC |
| *HvDJA07* | 1438 | 1432 | MYC |
| *HvDJA07* | 1556 | 1550 | MYC |
| *HvDJA07* | 1568 | 1574 | MYC |
| *HvDJA07* | 1594 | 1600 | MYC |
| *HvDJA07* | 652 | 644 | Zein metabolism regulation |
| *HvDJA08* | 264 | 259 | Abscisic acid responsive element |
| *HvDJA08* | 744 | 735 | Abscisic acid responsive element |
| *HvDJA08* | 753 | 748 | Abscisic acid responsive element |
| *HvDJA08* | 924 | 933 | Abscisic acid responsive element |
| *HvDJA08* | 1120 | 1125 | Abscisic acid responsive element |
| *HvDJA08* | 1133 | 1138 | Abscisic acid responsive element |
| *HvDJA08* | 264 | 258 | Abscisic acid responsive element |
| *HvDJA08* | 264 | 270 | Abscisic acid responsive element |
| *HvDJA08* | 71 | 65 | Anaerobic responsive element |
| *HvDJA08* | 1391 | 1385 | Anaerobic responsive element |
| *HvDJA08* | 1788 | 1782 | Anoxic responsive element |
| *HvDJA08* | 596 | 603 | Auxin-responsive element |
| *HvDJA08* | 1091 | 1097 | Auxin-responsive element |
| *HvDJA08* | 1526 | 1514 | Gibberellin-responsive element |
| *HvDJA08* | 1335 | 1329 | Light responsive element |
| *HvDJA08* | 264 | 258 | Light responsive element |
| *HvDJA08* | 753 | 759 | Light responsive element |
| *HvDJA08* | 807 | 813 | Light responsive element |
| *HvDJA08* | 1119 | 1113 | Light responsive element |
| *HvDJA08* | 1132 | 1126 | Light responsive element |
| *HvDJA08* | 1146 | 1140 | Light responsive element |
| *HvDJA08* | 1282 | 1288 | Light responsive element |
| *HvDJA08* | 2012 | 2018 | Light responsive element |
| *HvDJA08* | 1656 | 1663 | Light responsive element |
| *HvDJA08* | 1946 | 1940 | Light responsive element |
| *HvDJA08* | 1017 | 1026 | Light responsive element |
| *HvDJA08* | 326 | 332 | Light responsive element |
| *HvDJA08* | 539 | 545 | Light responsive element |
| *HvDJA08* | 930 | 936 | Light responsive element |
| *HvDJA08* | 984 | 990 | Light responsive element |
| *HvDJA08* | 1808 | 1802 | Low-temperature responsive element |
| *HvDJA08* | 191 | 196 | MeJA-responsive element |
| *HvDJA08* | 320 | 315 | MeJA-responsive element |
| *HvDJA08* | 370 | 375 | MeJA-responsive element |
| *HvDJA08* | 856 | 861 | MeJA-responsive element |
| *HvDJA08* | 958 | 963 | MeJA-responsive element |
| *HvDJA08* | 419 | 425 | MYB |
| *HvDJA08* | 1270 | 1264 | MYB |
| *HvDJA08* | 1855 | 1849 | MYB |
| *HvDJA08* | 652 | 646 | MYC |
| *HvDJA08* | 1074 | 1065 | Salicylic acid responsive element |
| *HvDJC79* | 1261 | 1255 | Abscisic acid responsive element |
| *HvDJC79* | 1262 | 1267 | Abscisic acid responsive element |
| *HvDJC79* | 1568 | 1563 | Abscisic acid responsive element |
| *HvDJC79* | 1985 | 1978 | Abscisic acid responsive element |
| *HvDJC79* | 366 | 372 | Anaerobic responsive element |
| *HvDJC79* | 1459 | 1465 | Anaerobic responsive element |
| *HvDJC79* | 1508 | 1502 | Anaerobic responsive element |
| *HvDJC79* | 1541 | 1535 | Anaerobic responsive element |
| *HvDJC79* | 1078 | 1071 | Auxin-responsive element |
| *HvDJC79* | 1069 | 1076 | Endosperm |
| *HvDJC79* | 1345 | 1337 | Light responsive element |
| *HvDJC79* | 1310 | 1318 | Light responsive element |
| *HvDJC79* | 378 | 385 | Light responsive element |
| *HvDJC79* | 817 | 823 | Light responsive element |
| *HvDJC79* | 967 | 973 | Light responsive element |
| *HvDJC79* | 1259 | 1250 | Light responsive element |
| *HvDJC79* | 1261 | 1255 | Light responsive element |
| *HvDJC79* | 1859 | 1865 | Light responsive element |
| *HvDJC79* | 1261 | 1255 | Light responsive element |
| *HvDJC79* | 1568 | 1574 | Light responsive element |
| *HvDJC79* | 308 | 302 | Light responsive element |
| *HvDJC79* | 599 | 593 | Light responsive element |
| *HvDJC79* | 1446 | 1452 | Low-temperature responsive element |
| *HvDJC79* | 152 | 157 | MeJA-responsive element |
| *HvDJC79* | 1052 | 1057 | MeJA-responsive element |
| *HvDJC79* | 1237 | 1242 | MeJA-responsive element |
| *HvDJC79* | 1430 | 1425 | MeJA-responsive element |
| *HvDJC79* | 1413 | 1407 | MYB |
| *HvDJC79* | 322 | 328 | MYB |
| *HvDJC79* | 370 | 376 | MYC |
| *HvDJA09* | 1159 | 1168 | Abscisic acid responsive element |
| *HvDJA09* | 1161 | 1155 | Abscisic acid responsive element |
| *HvDJA09* | 1162 | 1167 | Abscisic acid responsive element |
| *HvDJA09* | 1453 | 1448 | Abscisic acid responsive element |
| *HvDJA09* | 1520 | 1514 | Abscisic acid responsive element |
| *HvDJA09* | 1521 | 1526 | Abscisic acid responsive element |
| *HvDJA09* | 1525 | 1516 | Abscisic acid responsive element |
| *HvDJA09* | 1528 | 1533 | Abscisic acid responsive element |
| *HvDJA09* | 1538 | 1532 | Abscisic acid responsive element |
| *HvDJA09* | 1539 | 1544 | Abscisic acid responsive element |
| *HvDJA09* | 1893 | 1888 | Abscisic acid responsive element |
| *HvDJA09* | 1527 | 1533 | Abscisic acid responsive element |
| *HvDJA09* | 1527 | 1521 | Abscisic acid responsive element |
| *HvDJA09* | 1007 | 1000 | Auxin-responsive element |
| *HvDJA09* | 330 | 321 | Defence responsive element |
| *HvDJA09* | 222 | 215 | Gibberellin-responsive element |
| *HvDJA09* | 311 | 318 | Gibberellin-responsive element |
| *HvDJA09* | 1293 | 1286 | Gibberellin-responsive element |
| *HvDJA09* | 1329 | 1321 | Light responsive element |
| *HvDJA09* | 1161 | 1155 | Light responsive element |
| *HvDJA09* | 1453 | 1459 | Light responsive element |
| *HvDJA09* | 1520 | 1514 | Light responsive element |
| *HvDJA09* | 1538 | 1532 | Light responsive element |
| *HvDJA09* | 1893 | 1899 | Light responsive element |
| *HvDJA09* | 1069 | 1063 | Light responsive element |
| *HvDJA09* | 1161 | 1155 | Light responsive element |
| *HvDJA09* | 1453 | 1445 | Light responsive element |
| *HvDJA09* | 1520 | 1514 | Light responsive element |
| *HvDJA09* | 1527 | 1533 | Light responsive element |
| *HvDJA09* | 1538 | 1532 | Light responsive element |
| *HvDJA09* | 285 | 278 | Light responsive element |
| *HvDJA09* | 286 | 280 | Light responsive element |
| *HvDJA09* | 531 | 540 | Light responsive element |
| *HvDJA09* | 595 | 589 | Light responsive element |
| *HvDJA09* | 741 | 747 | Light responsive element |
| *HvDJA09* | 762 | 768 | Light responsive element |
| *HvDJA09* | 831 | 837 | Light responsive element |
| *HvDJA09* | 996 | 1002 | Light responsive element |
| *HvDJA09* | 1798 | 1792 | Light responsive element |
| *HvDJA09* | 1836 | 1830 | Light responsive element |
| *HvDJA09* | 1888 | 1882 | Light responsive element |
| *HvDJA09* | 1082 | 1075 | Light responsive element |
| *HvDJA09* | 330 | 324 | Light responsive element |
| *HvDJA09* | 1410 | 1404 | Low-temperature responsive element |
| *HvDJA09* | 1580 | 1574 | Low-temperature responsive element |
| *HvDJA09* | 638 | 633 | MeJA-responsive element |
| *HvDJA09* | 721 | 716 | MeJA-responsive element |
| *HvDJA09* | 1191 | 1185 | MYB |
| *HvDJA09* | 1656 | 1662 | MYB |
| *HvDJA09* | 1752 | 1758 | MYB |
| *HvDJA09* | 1656 | 1662 | MYB |
| *HvDJA09* | 373 | 379 | MYC |
| *HvDJA09* | 1101 | 1095 | MYC |
| *HvDJA09* | 1103 | 1109 | MYC |
| *HvDJA09* | 1440 | 1446 | MYC |
| *HvDJA09* | 337 | 328 | Salicylic acid responsive element |
| *HvDJC80* | 41 | 46 | Abscisic acid responsive element |
| *HvDJC80* | 134 | 139 | Abscisic acid responsive element |
| *HvDJC80* | 356 | 361 | Abscisic acid responsive element |
| *HvDJC80* | 804 | 810 | Abscisic acid responsive element |
| *HvDJC80* | 805 | 810 | Abscisic acid responsive element |
| *HvDJC80* | 1283 | 1278 | Abscisic acid responsive element |
| *HvDJC80* | 1535 | 1529 | Abscisic acid responsive element |
| *HvDJC80* | 1536 | 1541 | Abscisic acid responsive element |
| *HvDJC80* | 1911 | 1906 | Abscisic acid responsive element |
| *HvDJC80* | 803 | 811 | Abscisic acid responsive element |
| *HvDJC80* | 40 | 46 | Abscisic acid responsive element |
| *HvDJC80* | 40 | 34 | Abscisic acid responsive element |
| *HvDJC80* | 699 | 705 | Anaerobic responsive element |
| *HvDJC80* | 1138 | 1144 | Anaerobic responsive element |
| *HvDJC80* | 856 | 862 | Anoxic responsive element |
| *HvDJC80* | 928 | 922 | Anoxic responsive element |
| *HvDJC80* | 1920 | 1914 | Anoxic responsive element |
| *HvDJC80* | 1964 | 1958 | Anoxic responsive element |
| *HvDJC80* | 685 | 691 | Drought responsive element |
| *HvDJC80* | 1524 | 1530 | Drought responsive element |
| *HvDJC80* | 427 | 434 | Gibberellin-responsive element |
| *HvDJC80* | 803 | 812 | Light responsive element |
| *HvDJC80* | 1833 | 1826 | Light responsive element |
| *HvDJC80* | 40 | 46 | Light responsive element |
| *HvDJC80* | 133 | 127 | Light responsive element |
| *HvDJC80* | 355 | 349 | Light responsive element |
| *HvDJC80* | 690 | 684 | Light responsive element |
| *HvDJC80* | 703 | 709 | Light responsive element |
| *HvDJC80* | 802 | 793 | Light responsive element |
| *HvDJC80* | 804 | 810 | Light responsive element |
| *HvDJC80* | 867 | 873 | Light responsive element |
| *HvDJC80* | 1535 | 1529 | Light responsive element |
| *HvDJC80* | 804 | 810 | Light responsive element |
| *HvDJC80* | 1283 | 1289 | Light responsive element |
| *HvDJC80* | 1535 | 1529 | Light responsive element |
| *HvDJC80* | 1911 | 1917 | Light responsive element |
| *HvDJC80* | 1579 | 1585 | Light responsive element |
| *HvDJC80* | 1581 | 1575 | Light responsive element |
| *HvDJC80* | 925 | 931 | Light responsive element |
| *HvDJC80* | 1892 | 1898 | Light responsive element |
| *HvDJC80* | 1936 | 1930 | Light responsive element |
| *HvDJC80* | 366 | 373 | Light responsive element |
| *HvDJC80* | 1305 | 1311 | Low-temperature responsive element |
| *HvDJC80* | 27 | 22 | MeJA-responsive element |
| *HvDJC80* | 1083 | 1078 | MeJA-responsive element |
| *HvDJC80* | 1686 | 1691 | MeJA-responsive element |
| *HvDJC80* | 685 | 691 | MYB |
| *HvDJC80* | 1524 | 1530 | MYB |
| *HvDJC80* | 309 | 315 | MYB |
| *HvDJC80* | 428 | 422 | MYB |
| *HvDJC80* | 766 | 760 | MYB |
| *HvDJC80* | 1005 | 999 | MYB |
| *HvDJC80* | 309 | 315 | MYB |
| *HvDJC80* | 428 | 422 | MYB |
| *HvDJC80* | 766 | 760 | MYB |
| *HvDJC80* | 328 | 334 | MYC |
| *HvDJC80* | 479 | 485 | MYC |
| *HvDJC80* | 892 | 898 | MYC |
| *HvDJC80* | 1199 | 1193 | MYC |
| *HvDJC80* | 1271 | 1265 | MYC |
| *HvDJC80* | 1394 | 1403 | Salicylic acid responsive element |
| *HvDJC81* | 1092 | 1086 | Abscisic acid responsive element |
| *HvDJC81* | 1093 | 1098 | Abscisic acid responsive element |
| *HvDJC81* | 1091 | 1083 | Abscisic acid responsive element |
| *HvDJC81* | 578 | 584 | Anaerobic responsive element |
| *HvDJC81* | 752 | 759 | Auxin-responsive element |
| *HvDJC81* | 1206 | 1215 | Defence responsive element |
| *HvDJC81* | 1438 | 1432 | Drought responsive element |
| *HvDJC81* | 1553 | 1547 | Drought responsive element |
| *HvDJC81* | 698 | 704 | Light responsive element |
| *HvDJC81* | 1519 | 1513 | Light responsive element |
| *HvDJC81* | 1091 | 1100 | Light responsive element |
| *HvDJC81* | 1092 | 1086 | Light responsive element |
| *HvDJC81* | 908 | 914 | Light responsive element |
| *HvDJC81* | 1090 | 1081 | Light responsive element |
| *HvDJC81* | 1092 | 1086 | Light responsive element |
| *HvDJC81* | 1154 | 1148 | Light responsive element |
| *HvDJC81* | 190 | 199 | Light responsive element |
| *HvDJC81* | 1615 | 1607 | Light responsive element |
| *HvDJC81* | 778 | 784 | Light responsive element |
| *HvDJC81* | 1102 | 1096 | Light responsive element |
| *HvDJC81* | 1210 | 1216 | Light responsive element |
| *HvDJC81* | 1870 | 1876 | Light responsive element |
| *HvDJC81* | 1126 | 1131 | MeJA-responsive element |
| *HvDJC81* | 1438 | 1432 | MYB |
| *HvDJC81* | 1553 | 1547 | MYB |
| *HvDJC81* | 129 | 123 | MYB |
| *HvDJC81* | 1711 | 1705 | MYB |
| *HvDJC81* | 1940 | 1946 | MYB |
| *HvDJC81* | 515 | 522 | MYC |
| *HvDJC81* | 200 | 194 | MYC |
| *HvDJC81* | 742 | 736 | MYC |
| *HvDJC81* | 1545 | 1539 | MYC |
| *HvDJC81* | 1658 | 1652 | MYC |
| *HvDJC82* | 508 | 501 | Abscisic acid responsive element |
| *HvDJC82* | 1161 | 1156 | Abscisic acid responsive element |
| *HvDJC82* | 1161 | 1155 | Abscisic acid responsive element |
| *HvDJC82* | 1161 | 1167 | Abscisic acid responsive element |
| *HvDJC82* | 1747 | 1753 | Anaerobic responsive element |
| *HvDJC82* | 532 | 538 | Anoxic responsive element |
| *HvDJC82* | 691 | 697 | Anoxic responsive element |
| *HvDJC82* | 1665 | 1659 | Auxin-responsive element |
| *HvDJC82* | 1114 | 1123 | Defence responsive element |
| *HvDJC82* | 1362 | 1369 | Gibberellin-responsive element |
| *HvDJC82* | 1380 | 1372 | Light responsive element |
| *HvDJC82* | 1253 | 1260 | Light responsive element |
| *HvDJC82* | 1161 | 1155 | Light responsive element |
| *HvDJC82* | 1628 | 1622 | Light responsive element |
| *HvDJC82* | 1989 | 1995 | Light responsive element |
| *HvDJC82* | 1484 | 1474 | Light responsive element |
| *HvDJC82* | 1756 | 1750 | Light responsive element |
| *HvDJC82* | 1919 | 1913 | Light responsive element |
| *HvDJC82* | 134 | 140 | Light responsive element |
| *HvDJC82* | 1049 | 1043 | Light responsive element |
| *HvDJC82* | 697 | 691 | Low-temperature responsive element |
| *HvDJC82* | 23 | 28 | MeJA-responsive element |
| *HvDJC82* | 1572 | 1577 | MeJA-responsive element |
| *HvDJC82* | 1790 | 1795 | MeJA-responsive element |
| *HvDJC82* | 1805 | 1812 | MYB |
| *HvDJC82* | 627 | 633 | MYB |
| *HvDJC82* | 636 | 630 | MYB |
| *HvDJC82* | 975 | 981 | MYB |
| *HvDJC82* | 535 | 541 | MYB |
| *HvDJC82* | 865 | 859 | MYB |
| *HvDJC82* | 1344 | 1350 | MYB |
| *HvDJC82* | 1740 | 1734 | MYB |
| *HvDJC82* | 636 | 630 | MYB |
| *HvDJC82* | 946 | 952 | MYC |
| *HvDJC82* | 979 | 985 | MYC |
| *HvDJC82* | 1659 | 1668 | Zein metabolism regulation |
| *HvDJC83* | 1300 | 1294 | Anaerobic responsive element |
| *HvDJC83* | 1057 | 1048 | Defence responsive element |
| *HvDJC83* | 277 | 271 | Light responsive element |
| *HvDJC83* | 1057 | 1051 | Light responsive element |
| *HvDJC83* | 182 | 187 | MeJA-responsive element |
| *HvDJC83* | 958 | 953 | MeJA-responsive element |
| *HvDJC83* | 815 | 808 | MYB |
| *HvDJC83* | 136 | 142 | MYB |
| *HvDJC83* | 1866 | 1872 | MYB |
| *HvDJC83* | 1866 | 1872 | MYB |
| *HvDJC83* | 136 | 142 | MYB |
| *HvDJC83* | 451 | 457 | MYC |
| *HvDJC83* | 691 | 697 | MYC |
| *HvDJC83* | 738 | 744 | MYC |
| *HvDJC83* | 1116 | 1110 | MYC |
| *HvDJC83* | 1181 | 1175 | MYC |
| *HvDJC83* | 1804 | 1810 | MYC |
| *HvDJC84* | 923 | 928 | Abscisic acid responsive element |
| *HvDJC84* | 1354 | 1359 | Abscisic acid responsive element |
| *HvDJC84* | 1983 | 1977 | Abscisic acid responsive element |
| *HvDJC84* | 1984 | 1989 | Abscisic acid responsive element |
| *HvDJC84* | 922 | 928 | Abscisic acid responsive element |
| *HvDJC84* | 922 | 916 | Abscisic acid responsive element |
| *HvDJC84* | 334 | 328 | Anaerobic responsive element |
| *HvDJC84* | 226 | 235 | Defence responsive element |
| *HvDJC84* | 1946 | 1953 | Endosperm |
| *HvDJC84* | 1347 | 1354 | Gibberellin-responsive element |
| *HvDJC84* | 512 | 521 | Light responsive element |
| *HvDJC84* | 1068 | 1060 | Light responsive element |
| *HvDJC84* | 760 | 766 | Light responsive element |
| *HvDJC84* | 182 | 174 | Light responsive element |
| *HvDJC84* | 486 | 494 | Light responsive element |
| *HvDJC84* | 1060 | 1052 | Light responsive element |
| *HvDJC84* | 922 | 928 | Light responsive element |
| *HvDJC84* | 1353 | 1347 | Light responsive element |
| *HvDJC84* | 1983 | 1977 | Light responsive element |
| *HvDJC84* | 1983 | 1977 | Light responsive element |
| *HvDJC84* | 567 | 561 | Light responsive element |
| *HvDJC84* | 230 | 236 | Light responsive element |
| *HvDJC84* | 789 | 795 | Light responsive element |
| *HvDJC84* | 861 | 867 | Light responsive element |
| *HvDJC84* | 1465 | 1471 | Light responsive element |
| *HvDJC84* | 951 | 945 | Low-temperature responsive element |
| *HvDJC84* | 1352 | 1357 | MeJA-responsive element |
| *HvDJC84* | 53 | 47 | MYB |
| *HvDJC84* | 112 | 106 | MYB |
| *HvDJC84* | 568 | 574 | MYB |
| *HvDJC84* | 43 | 37 | MYB |
| *HvDJC84* | 564 | 558 | MYB |
| *HvDJC84* | 1053 | 1047 | MYB |
| *HvDJC84* | 1476 | 1482 | MYB |
| *HvDJC84* | 1827 | 1821 | MYB |
| *HvDJC84* | 112 | 106 | MYB |
| *HvDJC84* | 568 | 574 | MYB |
| *HvDJC84* | 420 | 414 | MYC |
| *HvDJC84* | 552 | 546 | MYC |
| *HvDJC84* | 558 | 552 | MYC |
| *HvDJC84* | 976 | 970 | MYC |
| *HvDJC84* | 984 | 978 | MYC |
| *HvDJC84* | 1796 | 1790 | MYC |
| *HvDJC84* | 777 | 784 | MYC |
| *HvDJC85* | 108 | 114 | Abscisic acid responsive element |
| *HvDJC85* | 109 | 114 | Abscisic acid responsive element |
| *HvDJC85* | 1642 | 1649 | Abscisic acid responsive element |
| *HvDJC85* | 335 | 341 | Anaerobic responsive element |
| *HvDJC85* | 1562 | 1568 | Anaerobic responsive element |
| *HvDJC85* | 264 | 258 | Anoxic responsive element |
| *HvDJC85* | 379 | 385 | Auxin-responsive element |
| *HvDJC85* | 1300 | 1306 | Auxin-responsive element |
| *HvDJC85* | 1928 | 1922 | Drought responsive element |
| *HvDJC85* | 1936 | 1930 | Drought responsive element |
| *HvDJC85* | 1230 | 1223 | Light responsive element |
| *HvDJC85* | 108 | 114 | Light responsive element |
| *HvDJC85* | 108 | 114 | Light responsive element |
| *HvDJC85* | 594 | 588 | Light responsive element |
| *HvDJC85* | 1293 | 1287 | Light responsive element |
| *HvDJC85* | 1548 | 1554 | Light responsive element |
| *HvDJC85* | 852 | 858 | Light responsive element |
| *HvDJC85* | 468 | 461 | Light responsive element |
| *HvDJC85* | 1347 | 1340 | Light responsive element |
| *HvDJC85* | 1267 | 1261 | Low-temperature responsive element |
| *HvDJC85* | 1461 | 1455 | Low-temperature responsive element |
| *HvDJC85* | 985 | 990 | MeJA-responsive element |
| *HvDJC85* | 1379 | 1384 | MeJA-responsive element |
| *HvDJC85* | 1591 | 1596 | MeJA-responsive element |
| *HvDJC85* | 1168 | 1162 | MYB |
| *HvDJC85* | 1928 | 1922 | MYB |
| *HvDJC85* | 1936 | 1930 | MYB |
| *HvDJC85* | 1175 | 1169 | MYB |
| *HvDJC85* | 1442 | 1436 | MYB |
| *HvDJC85* | 384 | 390 | MYB |
| *HvDJC85* | 188 | 181 | MYC |
| *HvDJC85* | 1477 | 1484 | MYC |
| *HvDJC85* | 1598 | 1604 | MYC |
| *HvDJC85* | 1595 | 1604 | Zein metabolism regulation |
| *HvDJC86* | 945 | 954 | Abscisic acid responsive element |
| *HvDJC86* | 1321 | 1314 | Abscisic acid responsive element |
| *HvDJC86* | 1432 | 1439 | Abscisic acid responsive element |
| *HvDJC86* | 1721 | 1726 | Abscisic acid responsive element |
| *HvDJC86* | 1780 | 1785 | Abscisic acid responsive element |
| *HvDJC86* | 1907 | 1914 | Abscisic acid responsive element |
| *HvDJC86* | 551 | 545 | Anaerobic responsive element |
| *HvDJC86* | 718 | 712 | Anaerobic responsive element |
| *HvDJC86* | 1684 | 1690 | Anaerobic responsive element |
| *HvDJC86* | 1832 | 1825 | Endosperm |
| *HvDJC86* | 392 | 385 | Gibberellin-responsive element |
| *HvDJC86* | 705 | 697 | Light responsive element |
| *HvDJC86* | 471 | 479 | Light responsive element |
| *HvDJC86* | 1135 | 1129 | Light responsive element |
| *HvDJC86* | 1145 | 1139 | Light responsive element |
| *HvDJC86* | 1157 | 1151 | Light responsive element |
| *HvDJC86* | 281 | 272 | Light responsive element |
| *HvDJC86* | 1720 | 1714 | Light responsive element |
| *HvDJC86* | 1779 | 1773 | Light responsive element |
| *HvDJC86* | 192 | 185 | Light responsive element |
| *HvDJC86* | 193 | 187 | Light responsive element |
| *HvDJC86* | 1192 | 1186 | Light responsive element |
| *HvDJC86* | 1956 | 1963 | Light responsive element |
| *HvDJC86* | 1711 | 1717 | Low-temperature responsive element |
| *HvDJC86* | 23 | 28 | MeJA-responsive element |
| *HvDJC86* | 1208 | 1203 | MeJA-responsive element |
| *HvDJC86* | 1772 | 1777 | MeJA-responsive element |
| *HvDJC86* | 1388 | 1381 | MYB |
| *HvDJC86* | 91 | 85 | MYB |
| *HvDJC86* | 194 | 200 | MYB |
| *HvDJC86* | 214 | 208 | MYB |
| *HvDJC86* | 392 | 398 | MYB |
| *HvDJC86* | 1870 | 1876 | MYB |
| *HvDJC86* | 1922 | 1928 | MYB |
| *HvDJC86* | 392 | 398 | MYB |
| *HvDJC86* | 91 | 85 | MYB |
| *HvDJC86* | 194 | 200 | MYB |
| *HvDJC86* | 62 | 56 | MYC |
| *HvDJC86* | 210 | 216 | MYC |
| *HvDJC86* | 670 | 676 | MYC |
| *HvDJC86* | 863 | 857 | MYC |
| *HvDJC86* | 1350 | 1356 | MYC |
| *HvDJC86* | 1768 | 1762 | MYC |
| *HvDJC86* | 1082 | 1072 | Salicylic acid responsive element |
| *HvDJB08* | 1203 | 1198 | Abscisic acid responsive element |
| *HvDJB08* | 1458 | 1452 | Abscisic acid responsive element |
| *HvDJB08* | 1459 | 1464 | Abscisic acid responsive element |
| *HvDJB08* | 1763 | 1757 | Anaerobic responsive element |
| *HvDJB08* | 1035 | 1029 | Anoxic responsive element |
| *HvDJB08* | 1225 | 1234 | Anoxic responsive element |
| *HvDJB08* | 1206 | 1200 | Auxin-responsive element |
| *HvDJB08* | 1395 | 1404 | Defence responsive element |
| *HvDJB08* | 1203 | 1209 | Light responsive element |
| *HvDJB08* | 1458 | 1452 | Light responsive element |
| *HvDJB08* | 1640 | 1634 | Light responsive element |
| *HvDJB08* | 1458 | 1452 | Light responsive element |
| *HvDJB08* | 136 | 130 | Light responsive element |
| *HvDJB08* | 336 | 346 | Light responsive element |
| *HvDJB08* | 801 | 795 | Light responsive element |
| *HvDJB08* | 1154 | 1160 | Light responsive element |
| *HvDJB08* | 1337 | 1331 | Light responsive element |
| *HvDJB08* | 1531 | 1537 | Light responsive element |
| *HvDJB08* | 1814 | 1808 | Light responsive element |
| *HvDJB08* | 1904 | 1910 | Light responsive element |
| *HvDJB08* | 1362 | 1355 | Light responsive element |
| *HvDJB08* | 1609 | 1616 | Light responsive element |
| *HvDJB08* | 1718 | 1725 | Light responsive element |
| *HvDJB08* | 853 | 858 | MeJA-responsive element |
| *HvDJB08* | 1449 | 1454 | MeJA-responsive element |
| *HvDJB08* | 1374 | 1368 | MYB |
| *HvDJB08* | 1435 | 1429 | MYB |
| *HvDJB08* | 1222 | 1216 | MYB |
| *HvDJB08* | 1374 | 1368 | MYB |
| *HvDJB08* | 59 | 53 | MYC |
| *HvDJB08* | 251 | 257 | MYC |
| *HvDJB08* | 320 | 314 | MYC |
| *HvDJB08* | 438 | 432 | MYC |
| *HvDJB08* | 1855 | 1849 | MYC |
| *HvDJB08* | 1894 | 1900 | MYC |
| *HvDJB08* | 1347 | 1338 | Salicylic acid responsive element |
| *HvDJB08* | 1136 | 1145 | Zein metabolism regulation |
| *HvDJC87* | 1051 | 1056 | Abscisic acid responsive element |
| *HvDJC87* | 1530 | 1523 | Abscisic acid responsive element |
| *HvDJC87* | 1729 | 1724 | Abscisic acid responsive element |
| *HvDJC87* | 1050 | 1056 | Abscisic acid responsive element |
| *HvDJC87* | 1050 | 1044 | Abscisic acid responsive element |
| *HvDJC87* | 207 | 201 | Anaerobic responsive element |
| *HvDJC87* | 356 | 350 | Anaerobic responsive element |
| *HvDJC87* | 1964 | 1958 | Anaerobic responsive element |
| *HvDJC87* | 1777 | 1783 | Anoxic responsive element |
| *HvDJC87* | 891 | 898 | Auxin-responsive element |
| *HvDJC87* | 682 | 688 | Drought responsive element |
| *HvDJC87* | 1100 | 1107 | Gibberellin-responsive element |
| *HvDJC87* | 1048 | 1039 | Light responsive element |
| *HvDJC87* | 632 | 622 | Light responsive element |
| *HvDJC87* | 634 | 627 | Light responsive element |
| *HvDJC87* | 1050 | 1056 | Light responsive element |
| *HvDJC87* | 1160 | 1166 | Light responsive element |
| *HvDJC87* | 1930 | 1924 | Light responsive element |
| *HvDJC87* | 1729 | 1735 | Light responsive element |
| *HvDJC87* | 1752 | 1746 | Light responsive element |
| *HvDJC87* | 1001 | 995 | Light responsive element |
| *HvDJC87* | 1570 | 1576 | Low-temperature responsive element |
| *HvDJC87* | 447 | 442 | MeJA-responsive element |
| *HvDJC87* | 1799 | 1794 | MeJA-responsive element |
| *HvDJC87* | 1353 | 1347 | MYB |
| *HvDJC87* | 1485 | 1479 | MYB |
| *HvDJC87* | 682 | 688 | MYB |
| *HvDJC87* | 1625 | 1631 | MYB |
| *HvDJC87* | 1083 | 1089 | MYB |
| *HvDJC87* | 1679 | 1685 | MYB |
| *HvDJC87* | 1353 | 1347 | MYB |
| *HvDJC87* | 1485 | 1479 | MYB |
| *HvDJC87* | 911 | 905 | MYC |
| *HvDJC87* | 956 | 962 | MYC |
| *HvDJC87* | 1574 | 1565 | Salicylic acid responsive element |
| *HvDJC87* | 1813 | 1804 | Salicylic acid responsive element |
| *HvDJC87* | 1240 | 1232 | Seed-specific regulation |
| *HvDJC87* | 1873 | 1882 | Zein metabolism regulation |
| *HvDJC88* | 496 | 501 | Abscisic acid responsive element |
| *HvDJC88* | 1547 | 1541 | Abscisic acid responsive element |
| *HvDJC88* | 1548 | 1553 | Abscisic acid responsive element |
| *HvDJC88* | 404 | 410 | Anaerobic responsive element |
| *HvDJC88* | 1910 | 1916 | Anoxic responsive element |
| *HvDJC88* | 1916 | 1922 | Anoxic responsive element |
| *HvDJC88* | 1947 | 1953 | Anoxic responsive element |
| *HvDJC88* | 1974 | 1980 | Anoxic responsive element |
| *HvDJC88* | 451 | 444 | Auxin-responsive element |
| *HvDJC88* | 962 | 968 | Auxin-responsive element |
| *HvDJC88* | 167 | 175 | Light responsive element |
| *HvDJC88* | 565 | 556 | Light responsive element |
| *HvDJC88* | 1831 | 1838 | Light responsive element |
| *HvDJC88* | 325 | 331 | Light responsive element |
| *HvDJC88* | 495 | 489 | Light responsive element |
| *HvDJC88* | 1547 | 1541 | Light responsive element |
| *HvDJC88* | 1547 | 1541 | Light responsive element |
| *HvDJC88* | 200 | 193 | Light responsive element |
| *HvDJC88* | 201 | 195 | Light responsive element |
| *HvDJC88* | 578 | 572 | Light responsive element |
| *HvDJC88* | 807 | 813 | Light responsive element |
| *HvDJC88* | 1058 | 1048 | Light responsive element |
| *HvDJC88* | 1139 | 1145 | Light responsive element |
| *HvDJC88* | 1208 | 1214 | Light responsive element |
| *HvDJC88* | 1488 | 1494 | Light responsive element |
| *HvDJC88* | 1502 | 1508 | Light responsive element |
| *HvDJC88* | 1907 | 1901 | Light responsive element |
| *HvDJC88* | 1913 | 1907 | Light responsive element |
| *HvDJC88* | 1919 | 1913 | Light responsive element |
| *HvDJC88* | 1944 | 1938 | Light responsive element |
| *HvDJC88* | 1950 | 1944 | Light responsive element |
| *HvDJC88* | 669 | 675 | Low-temperature responsive element |
| *HvDJC88* | 798 | 804 | Low-temperature responsive element |
| *HvDJC88* | 494 | 499 | MeJA-responsive element |
| *HvDJC88* | 1521 | 1516 | MeJA-responsive element |
| *HvDJC88* | 202 | 208 | MYB |
| *HvDJC88* | 579 | 585 | MYB |
| *HvDJC88* | 806 | 800 | MYB |
| *HvDJC88* | 883 | 889 | MYB |
| *HvDJC88* | 1438 | 1432 | MYB |
| *HvDJC88* | 1551 | 1545 | MYB |
| *HvDJC88* | 117 | 111 | MYB |
| *HvDJC88* | 851 | 857 | MYB |
| *HvDJC88* | 883 | 889 | MYB |
| *HvDJC88* | 202 | 208 | MYB |
| *HvDJC88* | 579 | 585 | MYB |
| *HvDJC88* | 806 | 800 | MYB |
| *HvDJC88* | 256 | 262 | MYC |
| *HvDJC88* | 299 | 293 | MYC |
| *HvDJC88* | 598 | 604 | MYC |
| *HvDJC88* | 630 | 621 | Zein metabolism regulation |
| *HvDJC88* | 1255 | 1246 | Zein metabolism regulation |
| *HvDJC89* | 146 | 141 | Abscisic acid responsive element |
| *HvDJC89* | 674 | 668 | Anaerobic responsive element |
| *HvDJC89* | 1620 | 1614 | Anoxic responsive element |
| *HvDJC89* | 1419 | 1426 | Auxin-responsive element |
| *HvDJC89* | 318 | 328 | Defence responsive element |
| *HvDJC89* | 1191 | 1182 | Defence responsive element |
| *HvDJC89* | 612 | 620 | Light responsive element |
| *HvDJC89* | 979 | 971 | Light responsive element |
| *HvDJC89* | 146 | 152 | Light responsive element |
| *HvDJC89* | 1486 | 1492 | Light responsive element |
| *HvDJC89* | 1488 | 1482 | Light responsive element |
| *HvDJC89* | 1599 | 1606 | Light responsive element |
| *HvDJC89* | 666 | 660 | MYB |
| *HvDJC89* | 1038 | 1032 | MYB |
| *HvDJC89* | 1485 | 1479 | MYB |
| *HvDJC89* | 1489 | 1495 | MYB |
| *HvDJC89* | 1805 | 1811 | MYB |
| *HvDJC89* | 666 | 660 | MYB |
| *HvDJC89* | 1038 | 1032 | MYB |
| *HvDJC89* | 1485 | 1479 | MYB |
| *HvDJC89* | 1489 | 1495 | MYB |
| *HvDJC89* | 555 | 561 | MYC |
| *HvDJC89* | 803 | 797 | MYC |
| *HvDJC89* | 1349 | 1355 | MYC |
| *HvDJC89* | 1560 | 1566 | MYC |
| *HvDJC89* | 1611 | 1617 | MYC |
| *HvDJC89* | 1726 | 1732 | MYC |
| *HvDJC89* | 1103 | 1094 | Salicylic acid responsive element |
| *HvDJC90* | 1691 | 1696 | Abscisic acid responsive element |
| *HvDJC90* | 1708 | 1713 | Abscisic acid responsive element |
| *HvDJC90* | 1733 | 1738 | Abscisic acid responsive element |
| *HvDJC90* | 1809 | 1800 | Abscisic acid responsive element |
| *HvDJC90* | 1224 | 1218 | Anaerobic responsive element |
| *HvDJC90* | 1638 | 1632 | Anoxic responsive element |
| *HvDJC90* | 1003 | 994 | Defence responsive element |
| *HvDJC90* | 1307 | 1313 | Drought responsive element |
| *HvDJC90* | 853 | 846 | Endosperm |
| *HvDJC90* | 386 | 392 | Light responsive element |
| *HvDJC90* | 614 | 620 | Light responsive element |
| *HvDJC90* | 1241 | 1235 | Light responsive element |
| *HvDJC90* | 1690 | 1684 | Light responsive element |
| *HvDJC90* | 1707 | 1701 | Light responsive element |
| *HvDJC90* | 1732 | 1726 | Light responsive element |
| *HvDJC90* | 1160 | 1166 | Light responsive element |
| *HvDJC90* | 1791 | 1800 | Light responsive element |
| *HvDJC90* | 445 | 439 | Low-temperature responsive element |
| *HvDJC90* | 1180 | 1185 | MeJA-responsive element |
| *HvDJC90* | 177 | 171 | MYB |
| *HvDJC90* | 1001 | 995 | MYB |
| *HvDJC90* | 1884 | 1890 | MYB |
| *HvDJC90* | 888 | 882 | MYB |
| *HvDJC90* | 924 | 918 | MYB |
| *HvDJC90* | 1307 | 1313 | MYB |
| *HvDJC90* | 1884 | 1890 | MYB |
| *HvDJC90* | 1001 | 995 | MYB |
| *HvDJC90* | 968 | 962 | MYC |
| *HvDJC90* | 95 | 104 | Salicylic acid responsive element |
| *HvDJC90* | 160 | 169 | Salicylic acid responsive element |
| *HvDJC90* | 329 | 337 | Seed-specific regulation |
| *HvDJC90* | 1015 | 1024 | Zein metabolism regulation |
| *HvDJC91* | 1405 | 1400 | Abscisic acid responsive element |
| *HvDJC91* | 1706 | 1711 | Abscisic acid responsive element |
| *HvDJC91* | 1723 | 1728 | Abscisic acid responsive element |
| *HvDJC91* | 1748 | 1753 | Abscisic acid responsive element |
| *HvDJC91* | 1824 | 1815 | Abscisic acid responsive element |
| *HvDJC91* | 366 | 360 | Anaerobic responsive element |
| *HvDJC91* | 1425 | 1431 | Auxin-responsive element |
| *HvDJC91* | 1549 | 1555 | Drought responsive element |
| *HvDJC91* | 250 | 243 | Gibberellin-responsive element |
| *HvDJC91* | 715 | 721 | Light responsive element |
| *HvDJC91* | 780 | 770 | Light responsive element |
| *HvDJC91* | 1405 | 1411 | Light responsive element |
| *HvDJC91* | 1705 | 1699 | Light responsive element |
| *HvDJC91* | 1722 | 1716 | Light responsive element |
| *HvDJC91* | 1747 | 1741 | Light responsive element |
| *HvDJC91* | 1806 | 1815 | Light responsive element |
| *HvDJC91* | 135 | 140 | MeJA-responsive element |
| *HvDJC91* | 1907 | 1913 | MYB |
| *HvDJC91* | 1549 | 1555 | MYB |
| *HvDJC91* | 1659 | 1653 | MYB |
| *HvDJC91* | 1907 | 1913 | MYB |
| *HvDJC91* | 940 | 946 | MYC |
| *HvDJC91* | 1002 | 1008 | MYC |
| *HvDJC91* | 1387 | 1381 | MYC |
| *HvDJC91* | 206 | 215 | Salicylic acid responsive element |
| *HvDJC91* | 763 | 772 | Salicylic acid responsive element |
| *HvDJC91* | 589 | 580 | Zein metabolism regulation |
| *HvDJC92* | 677 | 672 | Abscisic acid responsive element |
| *HvDJC92* | 819 | 824 | Abscisic acid responsive element |
| *HvDJC92* | 993 | 983 | Abscisic acid responsive element |
| *HvDJC92* | 994 | 1003 | Abscisic acid responsive element |
| *HvDJC92* | 996 | 1004 | Abscisic acid responsive element |
| *HvDJC92* | 997 | 1002 | Abscisic acid responsive element |
| *HvDJC92* | 1035 | 1040 | Abscisic acid responsive element |
| *HvDJC92* | 1650 | 1644 | Abscisic acid responsive element |
| *HvDJC92* | 1651 | 1656 | Abscisic acid responsive element |
| *HvDJC92* | 677 | 671 | Abscisic acid responsive element |
| *HvDJC92* | 818 | 824 | Abscisic acid responsive element |
| *HvDJC92* | 996 | 1002 | Abscisic acid responsive element |
| *HvDJC92* | 677 | 683 | Abscisic acid responsive element |
| *HvDJC92* | 818 | 812 | Abscisic acid responsive element |
| *HvDJC92* | 996 | 990 | Abscisic acid responsive element |
| *HvDJC92* | 996 | 1004 | Abscisic acid responsive element |
| *HvDJC92* | 1777 | 1783 | Anaerobic responsive element |
| *HvDJC92* | 1095 | 1101 | Auxin-responsive element |
| *HvDJC92* | 994 | 985 | Light responsive element |
| *HvDJC92* | 922 | 930 | Light responsive element |
| *HvDJC92* | 167 | 158 | Light responsive element |
| *HvDJC92* | 674 | 683 | Light responsive element |
| *HvDJC92* | 677 | 671 | Light responsive element |
| *HvDJC92* | 763 | 769 | Light responsive element |
| *HvDJC92* | 818 | 824 | Light responsive element |
| *HvDJC92* | 996 | 1002 | Light responsive element |
| *HvDJC92* | 1034 | 1028 | Light responsive element |
| *HvDJC92* | 1650 | 1644 | Light responsive element |
| *HvDJC92* | 1650 | 1644 | Light responsive element |
| *HvDJC92* | 486 | 492 | Light responsive element |
| *HvDJC92* | 639 | 633 | Light responsive element |
| *HvDJC92* | 1084 | 1090 | Low-temperature responsive element |
| *HvDJC92* | 1283 | 1277 | Low-temperature responsive element |
| *HvDJC92* | 257 | 263 | MYB |
| *HvDJC92* | 1053 | 1059 | MYB |
| *HvDJC92* | 1949 | 1955 | MYB |
| *HvDJC92* | 752 | 758 | MYB |
| *HvDJC92* | 1949 | 1955 | MYB |
| *HvDJC92* | 1053 | 1059 | MYB |
| *HvDJC92* | 450 | 444 | MYC |
| *HvDJC92* | 653 | 659 | MYC |
| *HvDJC92* | 1174 | 1180 | MYC |
| *HvDJC92* | 1256 | 1250 | MYC |
| *HvDJC92* | 1556 | 1562 | MYC |
| *HvDJC92* | 1835 | 1844 | Salicylic acid responsive element |
| *HvDJC92* | 609 | 617 | Seed-specific regulation |
| *HvDJC92* | 1610 | 1602 | Seed-specific regulation |

| \| **Table S4. Ka/Ks ratios of tandem duplication and segmental duplication gene pairs in barley.** \| \| \| \| \| \| \| \| \| \| --- \| --- \| --- \| --- \| --- \| --- \| --- \| --- \| --- \| \| **Gene pairs** \| \| **Ka** \| **Ks** \| **Ka/Ks** \| **Duplication Tpye** \|  \| **Selective pressure** \| \| *HvDJC28* \| *HvDJC29* \| 0.38 \| 0.76 \| 0.50 \| Tandem duplication \|  \| purifying selection \| \| *HvDJC67* \| *HvDJC68* \| 0.30 \| 0.41 \| 0.74 \| Tandem duplication \|  \| purifying selection \| \| *HvDJC68* \| *HvDJC69* \| 0.18 \| 0.24 \| 0.75 \| Tandem duplication \|  \| purifying selection \| \| *HvDJC01* \| *HvDJC23* \| 0.60 \| 2.06 \| 0.29 \| Segmental duplication \|  \| purifying selection \| \| *HvDJB01* \| *HvDJB02* \| 0.66 \| 1.38 \| 0.48 \| Segmental duplication \|  \| purifying selection \| \| *HvDJB01* \| *HvDJC49* \| 0.98 \| NaN \| NaN \| Segmental duplication \|  \|  \| \| *HvDJB01* \| *HvDJA04* \| 0.54 \| 3.50 \| 0.15 \| Segmental duplication \|  \| purifying selection \| \| *HvDJB02* \| *HvDJB05* \| 0.23 \| 0.55 \| 0.43 \| Segmental duplication \|  \| purifying selection \| \| *HvDJC10* \| *HvDJC35* \| 0.13 \| 0.78 \| 0.16 \| Segmental duplication \|  \| purifying selection \| \| *HvDJC11* \| *HvDJC36* \| 0.23 \| 1.12 \| 0.21 \| Segmental duplication \|  \| purifying selection \| \| *HvDJC11* \| *HvDJC83* \| 0.75 \| NaN \| NaN \| Segmental duplication \|  \|  \| \| *HvDJC12* \| *HvDJC34* \| 0.38 \| 0.99 \| 0.39 \| Segmental duplication \|  \| purifying selection \| \| *HvDJC48* \| *HvDJA03* \| 0.64 \| NaN \| NaN \| Segmental duplication \|  \|  \| \| *HvDJC49* \| *HvDJA02* \| 0.81 \| 2.54 \| 0.32 \| Segmental duplication \|  \| purifying selection \| \| *HvDJC49* \| *HvDJA06* \| 0.73 \| NaN \| NaN \| Segmental duplication \|  \|  \| \| *HvDJA02* \| *HvDJB08* \| 0.74 \| NaN \| NaN \| Segmental duplication \|  \|  \| \| *HvDJA03* \| *HvDJC80* \| 0.88 \| NaN \| NaN \| Segmental duplication \|  \|  \| \| *HvDJC63* \| *HvDJC85* \| 0.14 \| 0.96 \| 0.14 \| Segmental duplication \|  \| purifying selection \| \| *HvDJC73* \| *HvDJA06* \| 0.86 \| NaN \| NaN \| Segmental duplication \|  \|  \| \| *HvDJC73* \| *HvDJC76* \| 0.75 \| NaN \| NaN \| Segmental duplication \|  \|  \| \| *HvDJA06* \| *HvDJC80* \| 0.88 \| NaN \| NaN \| Segmental duplication \|  \|  \| \| *HvDJA06* \| *HvDJC87* \| 0.85 \| NaN \| NaN \| Segmental duplication \|  \|  \| \| *HvDJC76* \| *HvDJC80* \| 0.12 \| 1.18 \| 0.10 \| Segmental duplication \|  \| purifying selection \| \| *HvDJC85* \| *HvDJC87* \| 0.37 \| NaN \| NaN \| Segmental duplication \|  \|  \| |
| --- | --- | --- | --- | --- | --- | --- | --- | --- | --- | --- | --- | --- | --- | --- | --- | --- | --- | --- | --- | --- | --- | --- | --- | --- | --- | --- | --- | --- | --- | --- | --- | --- | --- | --- | --- | --- | --- | --- | --- | --- | --- | --- | --- | --- | --- | --- | --- | --- | --- | --- | --- | --- | --- | --- | --- | --- | --- | --- | --- | --- | --- | --- | --- | --- | --- | --- | --- | --- | --- | --- | --- | --- | --- | --- | --- | --- | --- | --- | --- | --- | --- | --- | --- | --- | --- | --- | --- | --- | --- | --- | --- | --- | --- | --- | --- | --- | --- | --- | --- | --- | --- | --- | --- | --- | --- | --- | --- | --- | --- | --- | --- | --- | --- | --- | --- | --- | --- | --- | --- | --- | --- | --- | --- | --- | --- | --- | --- | --- | --- | --- | --- | --- | --- | --- | --- | --- | --- | --- | --- | --- | --- | --- | --- | --- | --- | --- | --- | --- | --- | --- | --- | --- | --- | --- | --- | --- | --- | --- | --- | --- | --- | --- | --- | --- | --- | --- | --- | --- | --- | --- | --- | --- | --- | --- | --- | --- | --- | --- | --- | --- | --- | --- | --- | --- | --- | --- | --- | --- | --- | --- | --- | --- | --- | --- | --- | --- | --- | --- | --- | --- | --- | --- | --- | --- | --- | --- | --- | --- | --- |

| \| **Table S5. The expression profiles of HvDnaJs under salt stress.** \| \| \| \| \| \| --- \| --- \| --- \| --- \| --- \| \| **GeneID** \| **CK** \| **1h** \| **6h** \| **24h** \| \| *HvDJC01* \| 5.78 \| 8.23 \| 6.57 \| 2.80 \| \| *HvDJC02* \| 1.15 \| 0.97 \| 0.62 \| 0.39 \| \| *HvDJB02* \| 0.98 \| 1.85 \| 0.15 \| 0.34 \| \| *HvDJC09* \| 0.39 \| 1.18 \| 1.85 \| 4.91 \| \| *HvDJC10* \| 62.02 \| 61.81 \| 35.23 \| 27.81 \| \| *HvDJC11* \| 3.58 \| 0.80 \| 0.98 \| 8.17 \| \| *HvDJB03* \| 10.90 \| 32.07 \| 50.63 \| 28.68 \| \| *HvDJC12* \| 31.35 \| 30.97 \| 22.72 \| 13.55 \| \| *HvDJC14* \| 2.60 \| 1.05 \| 1.45 \| 3.67 \| \| *HvDJC29* \| 0.06 \| 0.05 \| 0.44 \| 0.93 \| \| *HvDJC33* \| 3.46 \| 17.61 \| 28.61 \| 23.32 \| \| *HvDJB05* \| 9.80 \| 9.48 \| 2.61 \| 2.00 \| \| *HvDJB06* \| 0.09 \| 0.00 \| 0.00 \| 0.00 \| \| *HvDJC40* \| 1.52 \| 0.53 \| 0.12 \| 1.07 \| \| *HvDJC43* \| 0.23 \| 0.25 \| 0.09 \| 0.17 \| \| *HvDJC46* \| 0.00 \| 2.86 \| 3.43 \| 2.21 \| \| *HvDJC47* \| 19.52 \| 25.09 \| 8.45 \| 4.76 \| \| *HvDJC48* \| 2.79 \| 3.76 \| 11.48 \| 5.53 \| \| *HvDJC49* \| 6.13 \| 3.45 \| 4.73 \| 10.41 \| \| *HvDJC51* \| 6.10 \| 6.54 \| 2.73 \| 2.97 \| \| *HvDJC52* \| 5.85 \| 4.90 \| 2.53 \| 2.78 \| \| *HvDJC54* \| 58.55 \| 38.15 \| 26.63 \| 30.64 \| \| *HvDJC58* \| 4.01 \| 1.84 \| 1.42 \| 1.13 \| \| *HvDJC59* \| 30.19 \| 7.65 \| 8.48 \| 2.36 \| \| *HvDJC63* \| 58.22 \| 43.75 \| 23.25 \| 30.65 \| \| *HvDJC66* \| 3.78 \| 2.66 \| 13.30 \| 8.54 \| \| *HvDJB07* \| 2.23 \| 3.00 \| 3.21 \| 5.15 \| \| *HvDJC76* \| 21.13 \| 19.45 \| 12.00 \| 9.99 \| \| *HvDJC79* \| 9.14 \| 19.03 \| 47.12 \| 61.65 \| \| *HvDJC83* \| 3.91 \| 6.04 \| 8.24 \| 4.97 \| \| *HvDJB08* \| 17.75 \| 16.75 \| 6.29 \| 5.86 \| \| *HvDJC87* \| 17.28 \| 28.22 \| 9.39 \| 7.16 \| \| *HvDJC88* \| 3.61 \| 3.98 \| 1.16 \| 0.98 \| \| *HvDJC89* \| 1.83 \| 1.80 \| 0.79 \| 0.72 \| \| *HvDJC90* \| 7.09 \| 5.02 \| 1.05 \| 2.41 \| \| *HvDJC91* \| 6.61 \| 4.71 \| 1.26 \| 2.18 \| \| *HvDJC92* \| 1.08 \| 1.76 \| 0.67 \| 2.83 \| |
| --- | --- | --- | --- | --- | --- | --- | --- | --- | --- | --- | --- | --- | --- | --- | --- | --- | --- | --- | --- | --- | --- | --- | --- | --- | --- | --- | --- | --- | --- | --- | --- | --- | --- | --- | --- | --- | --- | --- | --- | --- | --- | --- | --- | --- | --- | --- | --- | --- | --- | --- | --- | --- | --- | --- | --- | --- | --- | --- | --- | --- | --- | --- | --- | --- | --- | --- | --- | --- | --- | --- | --- | --- | --- | --- | --- | --- | --- | --- | --- | --- | --- | --- | --- | --- | --- | --- | --- | --- | --- | --- | --- | --- | --- | --- | --- | --- | --- | --- | --- | --- | --- | --- | --- | --- | --- | --- | --- | --- | --- | --- | --- | --- | --- | --- | --- | --- | --- | --- | --- | --- | --- | --- | --- | --- | --- | --- | --- | --- | --- | --- | --- | --- | --- | --- | --- | --- | --- | --- | --- | --- | --- | --- | --- | --- | --- | --- | --- | --- | --- | --- | --- | --- | --- | --- | --- | --- | --- | --- | --- | --- | --- | --- | --- | --- | --- | --- | --- | --- | --- | --- | --- | --- | --- | --- | --- | --- | --- | --- | --- | --- | --- | --- | --- | --- | --- | --- | --- | --- | --- | --- | --- | --- | --- | --- | --- |

| **Table S6. Primers used for RT-PCR and qRT-PCR.** | | |
| --- | --- | --- |
| **Primer name** | **Primer sequence (5'-3')** | **Product length(bp)** |
| qHvDJC09-F | GACACCTCCACAGGAAGACG | 137 |
| qHvDJC09-R | TGCTGACCCTTGTCTTGGAC |  |
| qHvDJB03-F | GTGGAAGAAGGGCACGAAGA | 189 |
| qHvDJB03-R | GTCAGGTGTACCGTGTAGCC |  |
| qHvDJC33-F | CAACAAGAGGTCGAGGGCTT | 128 |
| qHvDJC33-R | TCCTGCATCATCCCGTCAAG |  |
| qHvDJC46-F | ATGTACGATGTTCTGGCAGT | 188 |
| qHvDJC46-R | CGCAGCTGGATGTCGTA |  |
| qHvDJC59-F | AGGGGCGACTACTACAAGGT | 199 |
| qHvDJC59-R | GCACTTTGGTTGCAGGGTC |  |
| q_Tublin F | AGTGTCCTGTCCACCCACTC | 120 |
| q_Tublin R | AGCATGAAGTGGATCCTTGG |  |
